# Genome-based reclassification of the family *Stappiaceae* and assessment of environmental forcing with the report of two novel taxa, *Flexibacterium corallicola* gen. nov., sp. nov., and *Nesiotobacter zosterae* sp. nov., isolated from coral and seagrass

**DOI:** 10.1371/journal.pone.0322500

**Published:** 2025-05-15

**Authors:** Mi-Jeong Park, Jinnam Kim, Yun Jae Kim, Jihyun Yu, Hyein Jin, Seonok Woo, Young-Gun Zo, Kae Kyoung Kwon

**Affiliations:** 1 Marine Biotechnology and Bioresource Research Department, Korea Institute of Ocean Science and Technology, Busan, Republic of Korea; 2 Department of Biology, Kyungsung University, Busan, Republic of Korea; 3 Marine Technology and Convergence Engineering, KIOST School, University of Science and Technology, Daejeon, Republic of Korea.; King Abdulaziz University, SAUDI ARABIA

## Abstract

Two novel strains, MaLMAid0302^T^ and SPO723^T^, isolated from coral and eelgrass, respectively, were distinguished from other *Stappiaceae* species based on phenotypic, biochemical, phylogenetic, and chemotaxonomic traits. Taxonomic challenges within the family *Stappiaceae* were addressed using a taxogenomic approach with iterative clustering, establishing an optimal average amino acid identity (AAI) threshold (71.92–72.88%) for genus delineation. This analysis led to major taxonomic revisions, including the establishment of new genera—*Parapolycladidibacter*, *Astericibacter*, *Flexibacterium*, *Aliiroseibium*, *Laciiroseibium*, *Soliroseibium*, *Novilabrenzia*, *Litoriroseibium*, and *Algilabrenzia*—as well as the reassignment of several species: *Hongsoonwoonella albiluteola* comb. nov., *Parapolycladidibacter stylochi* gen. nov., comb. nov., *Astericibacter flavus* gen. nov., comb. nov., *Nesiotobacter exalbescens* comb. nov., *Aliiroseibium hamelinense* gen. nov., comb. nov., *Laciiroseibium aquae* gen. nov., comb. nov., *Soliroseibium sediminis* gen. nov., comb. nov., *Novilabrenzia suaedae* gen. nov., comb. nov., *Novilabrenzia litorale* gen. nov., comb. nov., *Litoriroseibium aestuarii* gen. nov., comb. nov., *Litoriroseibium limicola* gen. nov., comb. nov., and *Algilabrenzia polysiphoniae* gen. nov., comb. nov. Given this extensive taxonomic reclassification of the family *Stappiaceae*, strain SPO723^T^ (=KCCM 42324^T^ = JCM 14066^T^) was classified as *Nesiotobacter zosterae* sp. nov., and *Flexibacterium corallicola* MaLMAid0302^T^ (=KCTC 92348^T^ = JCM 35474^T^) was designated as the type species of the newly established genus *Flexibacterium*. Close phylogenetic ties to *Pseudovibrio*, known for symbiosis, prompted analysis of niche-specific genetic compositions. Canonical Correspondence Analysis attributed 64% of genomic variation to phylogenetic forcing and 36% to environmental forcing. Functional adaptations included pectin and aromatic compound degradation in sediment strains, nitrogen reduction in flatworm strains, and sulfur metabolism in coral strains. The eelgrass strain exhibited dTDP-L-rhamnose synthesis, potentially aiding biofilm formation for adhesion in dynamic environments. These findings emphasize the roles of both environmental and phylogenetic forcing in shaping genomic diversity and highlight the ecological importance of the family *Stappiaceae* in marine habitat-associated niches.

## Introduction

The family *Stappiaceae* was added to the class *Alphaproteobacteria* after a noteworthy reclassification in 2020 [1]. The process leading to the proposal of the type genus *Stappia* within *Stappiaceae* was lengthy, spanning approximately 50 years. In 2024, the genus *Polycladidibacter* was validly published under ICNP nomenclatural status [2]. As a result, the family *Stappiaceae* now comprises six genera that are both validly published under the ICNP and recognized as correct names: *Hongsoonwoonella* [3], *Pannonibacter* [4], *Polycladidibacter* [5], *Pseudovibrio* [6], *Roseibium* [7], and *Stappia* [8]. Microorganisms belonging to the genus *Pseudovibrio* have predominantly been isolated from marine invertebrates, such as sponges, corals, and tunicates. Genomic analysis has revealed their metabolic versatility in supplying essential cofactors to their host organisms [9,10]. These bacteria have also gained a competitive advantage over others by harnessing biosynthetic gene clusters and producing key compounds like tropodithietic acid, bacteriocin, and terpenes [11]. Consequently, research into these compounds as natural antibacterial agents has garnered substantial attention [12]. Given *Pseudovibrio*’s potential as a source of secondary metabolites and natural products, researchers are increasingly interested in exploring its diversity and bioactive compounds [13]. The rich repertoire of secondary metabolites in *Pseudovibrio* suggests it could serve as a valuable resource for discovering novel natural products with various biotechnological applications. Given that many species within the family *Stappiaceae* and the genus *Pseudovibrio* exhibit biogenic properties [14–16], understanding the niche-specific functions of the family *Stappiaceae* can offer valuable insights into microbial ecology, biotechnology, medicine, and other fields. This knowledge paves the way for developing bioactive compounds with diverse applications. Despite growing recognition of *Stappiaceae*’s ecological importance, its taxonomic classification and functional diversity remain relatively underexplored. Traditional taxonomy, relying on phenotypic traits and 16S rRNA sequences, often falls short in discerning niche-specific functions. A strain’s genome comprises genes inherited from ancestors and those acquired from the environment. The former process, known as phylogenetic forcing, reflects evolutionary history, while the latter, termed environmental forcing, is shaped by adaptation. Under the genome economy hypothesis, which suggests the continuous purging of non-contributory genes, genes acquired from the environment likely serve essential roles in survival within specific niches [17,18]. In this context, taxogenomics—a powerful approach combining genomics and taxonomy—emerges as a critical tool for unraveling taxonomic complexities and functional adaptations, surpassing traditional methods [19–21]. Our study began with the taxonomic analysis of two additional *Stappiaceae* species, isolated from coral (*Scleronephthya gracillimum*) and eelgrass (*Zostera marina*). Through taxogenomic analysis, we sought to determine their taxonomic identities at both the species and genus levels. To resolve taxonomic issues within the family *Stappiaceae*, we introduced a repetitive clustering and evaluation method to propose an optimal AAI threshold for genus classification, enabling reclassification [22]. This effort marks the first comprehensive taxogenomic analysis of the entire *Stappiaceae* species. Subsequently, we compared the genome contents of newly classified *Pseudovibrio* relatives to assess the roles of phylogenetic and environmental forcing in shaping their metabolic versatility. By elucidating niche-specific metabolisms across habitats, we aim to establish a foundation for future metagenomic research and interpretations of their ecological roles.

## Materials and methods

### Isolation and culture

Strain MaLMAid0302^T^ was isolated in July 2021 from the coral species *Scleronephthya gracillimum* collected near Jeju Island, Republic of Korea (33°13′40″N, 126°34′07″E). Strain SPO723^T^ was isolated in November 2008 from a leaf of *Zostera marina* found on Weno Island, Chuuk State, Micronesia (50°03′N, 73°03′E). For isolation, the substratum of the coral sample or a piece of the leaf sample was homogenized and diluted with sterilized seawater. The diluted solution was spread onto Reasoner’s 2A (R2A) agar supplemented with 1.5% (w/v) sodium chloride (MR2A) for coral samples and incubated at 25°C for 7 days. For plant leaf samples, the solution was spread onto marine agar 2216 (MA; Difco) and incubated at 30°C for 2–3 days. Individual colonies were picked from MR2A or MA, and the morphologically distinct strains MaLMAid0302^T^ and SPO723^T^ were selected for further characterization. Purified strains were cultivated on MA at 25°C or 30°C and stored at –80°C in a medium supplemented with 20% (v/v) glycerol. Strain MaLMAid0302^T^ has been deposited in the Korean Collection for Type Cultures (KCTC 92348^T^) and the Japan Collection of Microorganisms (JCM 35474^T^). Strain SPO723^T^ is deposited in the Korean Culture Center of Microorganisms (KCCM 42324^T^) and the Japan Collection of Microorganisms (JCM 14066^T^). For comparative phenotypic and biochemical analyses, type strains of the *Pseudovibrio* species were obtained from the Leibniz Institute DSMZ and KCTC. These strains were cultivated on MA at 28°C. As the bacterial strains were isolated from publicly accessible marine environments and the study did not involve human or animal subjects, no ethical approval was required.

### Phylogenetic analysis based on 16S rRNA gene sequences

Genomic DNA was extracted from the isolated strains using a Qiagen genomic DNA extraction kit following the manufacturer’s protocol. The 16S rRNA gene was amplified using universal bacterial primers 27F and 1492R, and the PCR product was purified using a MinElute PCR Purification Kit (Qiagen). Sequencing was performed by Macrogen (Seoul, Republic of Korea) on a 3730XL DNA Analyzer (Applied Biosystems), and the sequences were assembled using MEGA version X software. To determine phylogenetic relationships, 16S rRNA gene sequences of closely related taxa were retrieved from the GenBank [23] and EzBioCloud databases [24]. Sequence alignment was carried out using CLUSTAL_W in MEGA X [25]. Phylogenetic trees were constructed with 1,000 bootstrap replicates using three methods: the neighbor-joining method (NJ) [26] with the Kimura 2 parameter model [27], the maximum-likelihood (ML) method [28], and the maximum-parsimony (MP) method [29]. The partial deletion option (<80%) was applied during tree construction. *Martellela mediterranea* MACL11^T^ (AY649762) and *Hyphomicrobium vulgare* ATCC 27500^T^ (Y14302) were used as outgroup taxa. The 16S rRNA gene sequences of strains MaLMAid0302^T^ and SPO723^T^ have been deposited in GenBank under the accession numbers OP606461 and DQ660386, respectively.

### Phenotypic and chemotaxonomic characteristics of strains

All strains used for phenotypic and chemotaxonomic testing were routinely cultivated on marine agar at 28°C for 3 days. The morphological characteristics of strains MaLMAid0302^T^ and SPO723^T^ were observed using transmission electron microscopy (HT-7800, Hitachi Ltd.) after negative staining with 2% (w/v) uranyl acetate. Anaerobic growth was assessed using the BD GasPak EZ pouch system on marine agar (MA; BD Difco) at 28°C for 7 days. Temperature growth ranges (4–40°C, at intervals of 5°C), pH tolerance (4.0–11.0, at increments of 1.0), and NaCl tolerance (0–8.0%, w/v, in 0.5% increments) were evaluated in marine broth (MB; BD) or modified ZoBell 2216 broth over a 7-day period. pH adjustments were made using specific buffers: MES (pH 4–6), HEPES (pH 6–8), AMPSO (pH 8–10), or sodium bicarbonate/sodium carbonate (pH 11). Growth was monitored by measuring absorbance (OD600) every 12 hours using a spectrophotometer. Catalase activity was tested by bubble formation in 3% (v/v) hydrogen peroxide, and oxidase activity by oxidation of 1% (w/v) tetramethyl-p-phenylenediamine. Enzyme activity, carbon source utilization, and other biochemical properties were analyzed using API 20E, API 20NE, and API ZYM kits with sodium chloride concentration adjusted to 3%. Results were recorded after cultivation at 28°C for several days. For chemotaxonomic analysis, strains were grown on marine agar for 3 days at 25°C. Cellular fatty acid methyl esters were prepared following the Sherlock Microbial Identification System (MIDI) version 6.1 protocol. Fatty acid profiles were separated and identified using an Agilent 6890N network GC system (Agilent Technologies) with the TSBA6 library.

### Information on genomic sequences

Strains MaLMAid0302^T^ and SPO723^T^ were sequenced using PacBio 10K (Pacific Biosciences) and Illumina TruSeq technology, respectively, by CJ Bioscience and Macrogen in Seoul, Republic of Korea. For the strain MaLMAid0302^T^, the raw reads were assembled into a 4.96 Mb genome with 967X coverage. For the strain SPO723^T^, the assembly was performed using the SPAdes v3.15.0 assembly tool [30], resulting in a 4.29 Mb genome with 145X coverage. Both genome sequences have been deposited in the NCBI GenBank under accession numbers JARRCW00000000.1 and JAWXVZ000000000.1. To facilitate a comparative taxogenomic analysis, genomes from other species within the family *Stappiaceae* were selected as reference targets. Genome assembly statistics, including the number of contigs, total bases, N50, and G + C content, were gathered using the GAAS-toolkit. The quality of the genome, completeness, and contamination were evaluated using CheckM v1.2.3 [31]. Genome annotations were performed using Prokka version 1.14.6 [32], which identified key features such as coding DNA sequences (CDS), rRNA, CRISPR repeat regions, tRNA, and tmRNA.

### Genome-based phylogenetic analysis

For the genome-based phylogenetic analysis, a core gene phylogeny was constructed for each genome based on Prokka annotations [32]. To provide a comprehensive comparison, two additional species from the order *Rhizobiales*—*Martelella mediterranea* DSM17316^T^ and *Hyphomicrobium nitrativorans* NL23^T^—were included as outgroups. The genome-based phylogenetic analysis was performed using PhyloPhlAn v3.1.68 [33], and the detailed steps were carried out using its built-in tools as follows. The core gene phylogeny was established using 400 universal protein markers extracted from the genomes, which were then sorted using USEARCH v5.2.32 [34]. The selected universal proteins underwent multiple sequence alignment using MUSCLE v3.6 [35]. A phylogenetic tree was constructed from this alignment using FastTree v2.1.10 [36], with 1,000 bootstrap replicates to ensure robust statistical support for the tree’s topology [37]. The constructed genome-based phylogenetic tree was evaluated using clustering trees based on ANI and AAI distance matrices. The ANI and AAI score matrices were obtained using the genomic index calculation methods described later. Tree-building was performed from the distance matrices using the R package ape. A tree was constructed using the balanced minimum evolution algorithm implemented in the *FastMe* [38] function of *ape* [39], with the nearest neighbor interchange and subtree pruning and regrafting parameters enabled.

### Genus and species delineation based on genomic indices

In genus and species delineation, genomic indices such as Average Amino Acid Identity (AAI), Percentage of Conserved Proteins (POCP), *digital* DNA-DNA Hybridization (*d*DDH), and Average Nucleotide Identity (ANI) are widely used standards in taxogenomics. In this study, ANI and *d*DDH were employed to delineate species, with ANI calculated using the OrthoANI-usearch tool [40] and *d*DDH computed with the Genome-to-Genome Distance Calculator (GGDC) v3.0 [41]. For genus delineation, AAI and POCP were utilized. POCP was assessed by comparing genomes using BLASTP, where proteins with an E-value below 10^-5 and an identity of 50% or higher were considered conserved proteins, and the ratio of conserved proteins was calculated [42]. AAI was calculated using the AAI calculator from the Kostas Lab [43], with repetitive clustering to determine a clear reference value for genus delineation. These metrics collectively provided a robust approach for evaluating genetic relatedness and facilitating accurate taxonomic classification at both the genus and species levels [22].

### Comparative and functional genomics analysis

GFF file outputs generated by Prokka were used as inputs for the pan-genome pipeline Roary software [44] to determine coding sequence (CDS) clusters. A clustering criterion of > 71.92% average amino acid identity (AAI) was applied, as the genomes were relatively distantly related and classified into several distinct genera. The distribution of CDS clusters across bacterial genomes was visualized using Phandango [45]. To identify genes specific to bacteria inhabiting particular ecological niches, canonical correspondence analysis (CCA) was conducted using the vegan package in R. CDS clusters served as species data, while habitat types, such as sponge, coral, flatworm, eelgrass, and sediment, were used as environmental variables. CCA identified CDS associated with specific habitat types. Additionally, CDS exclusively present in a taxon from a specific habitat and absent in all related taxa were considered habitat-specific genes. Functions of CDS were mapped to KEGG metabolic pathways using BlastKOALA [46] with a criterion of allowing a maximum of one missing component. For CDS associated with specific niches by CCA, gene-set enrichment analysis was performed using ShinyGO [47] to infer metabolic pathways responsive to ecological niches.

## Results

### Isolation and general genomic features of the strains MaLMAid0302^T^ and SPO723^T^

The coral species *Scleronephthya gracillimum* from which strain MaLMAid0302^T^ was isolated and the eelgrass species *Zostera marina* from which strain SPO723^T^ was isolated represent different hosts, yet both strains share the commonality of originating from marine organisms. When cultured on marine agar, strain MaLMAid0302^T^ formed circular, smooth, convex, non-transparent, creamy-white colonies, while strain SPO723^T^ produced light pink to cream-colored circular, convex, and erose margin colonies with an average diameter of 1.0 mm. The genomes of strains MaLMAid0302^T^ and SPO723^T^, sequenced using PacBio 10K (Pacific Biosciences) and Illumina TruSeq technology, respectively, revealed that strain MaLMAid0302^T^ has a 4.96 Mb genome, while strain SPO723^T^ has a 4.29 Mb genome. Both genomes showed high quality, with completeness exceeding 95% and contamination below 1%. Additionally, strain MaLMAid0302^T^ contains seven rRNA sets and has a G + C content of 49.4%, whereas strain SPO723^T^ has a G + C content of 55.5%. The genomic data confirmed that these strains belong to the family *Stappiaceae*, prompting further in-depth analysis to explore their novelty, characteristics, and taxonomic significance.

### 16S rRNA gene sequence-based phylogenetic analysis

**Fig 1:**
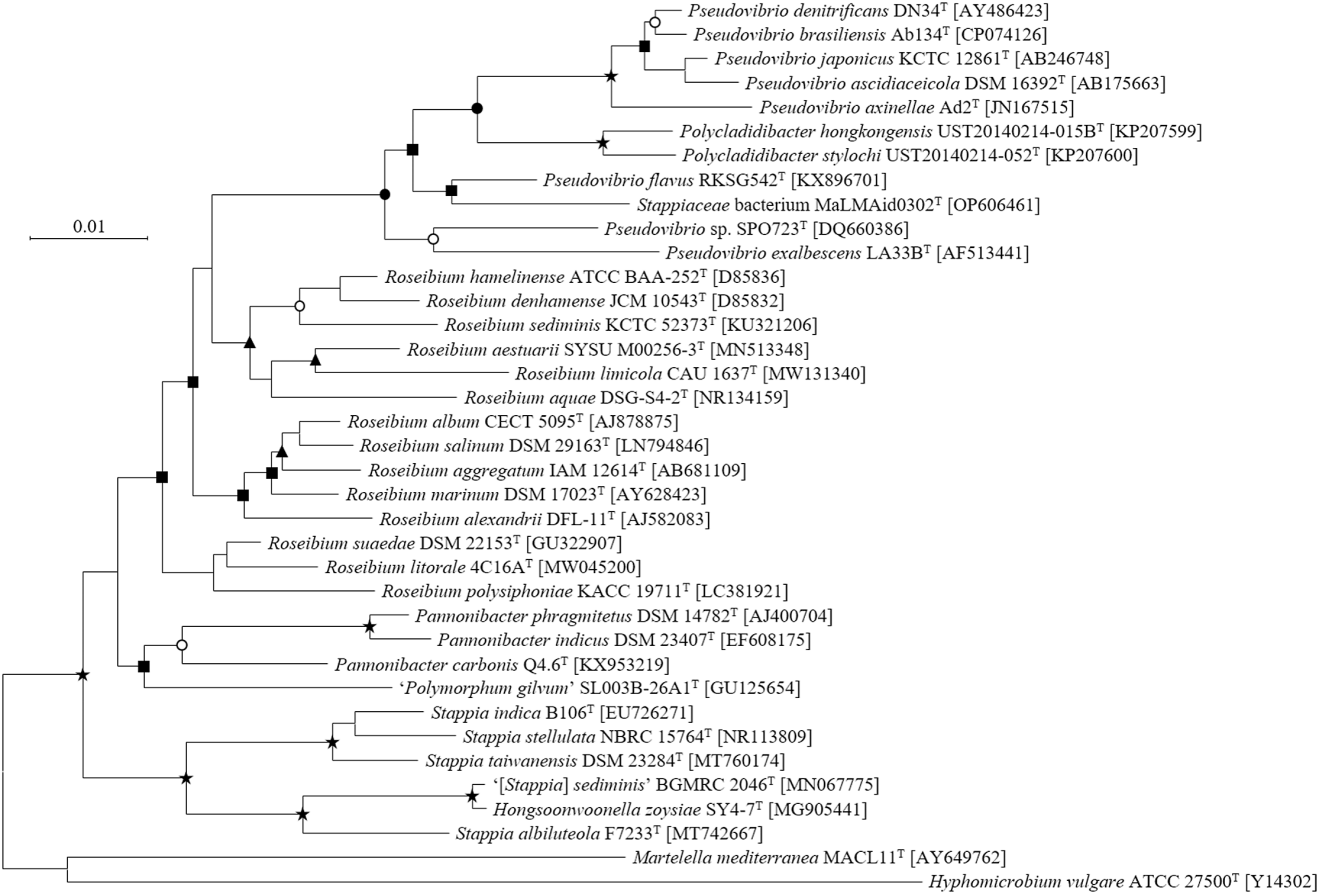
Phylogenetic relationships based on 16S rRNA gene sequences of members in the family *Stappiaceae.* The tree was reconstructed using the neighbor-joining method inferred by the Kimura2 parameter distance model with the bootstrap values of 1000 replicates. Nodes recovered by three (N-J, MP, and ML) methods with >90% (★), > 70% (●), and > 50% (ϒ), at least one lower than 50% (▲), and recovered by two methods (■) are designated by symbols. Bar, 0.01 substitutions per nucleotide position.

Further investigation using 16S rRNA gene sequence-based phylogenetic analysis of strains MaLMAid0302^T^ and SPO723^T^ confirmed their affiliation within the genus *Pseudovibrio*. Both strains exhibited high sequence similarity to various *Pseudovibrio* species, with strain MaLMAid0302^T^ showing 97.79% similarity to *Pseudovibrio flavus* RKSG542^T^ and strain SPO723^T^ showing 97.21% similarity to the same strain. Both strains also exhibited relatively high similarity to *Roseibium* species, indicating potential classification challenges within these genera. Phylogenetic analysis revealed that both strains clustered with members of *Pseudovibrio*, though strain MaLMAid0302^T^ did not form a strong clade with *P. flavus*, and strain SPO723^T^ instead clustered with *P. exalbescens* (Fig 1). Additionally, *Roseibium* species did not form a monophyletic group, and certain *Stappia* species shared a clade with *Hongsoonwoonella zoysiae*. These findings suggest that the classification of the family *Stappiaceae* may need further revision to account for these phylogenetic nuances. The analysis emphasized the complexity of taxonomic classification within the family *Stappiaceae*, highlighting the need for refined taxonomic approaches, particularly when species exhibit high levels of similarity yet do not always form distinct clades.

### Phenotypic and chemotaxonomic characteristics

Strains MaLMAid0302^T^ and SPO723^T^ are Gram-stain negative bacteria, with strain MaLMAid0302^T^ being a strict aerobe and strain SPO723^T^ being a facultative anaerobe. Under transmission electron microscopy (TEM), strain MaLMAid0302^T^ displayed peritrichous flagella (S2 FigA), whereas strain SPO723^T^ lacked flagella under scanning electron microscopy (SEM) (S2 FigB), emphasizing their structural differences. Both strains thrived in mesophilic and neutrophilic conditions and required NaCl for growth, consistent with other members of the family *Stappiaceae.* The polar lipid composition of both strains included PE, PG, DPG, AL, PL, and L, aligning with that of their closest relatives (S3 Fig). Phenotypic and chemotaxonomic tests were conducted and compared with eight closely related species (*Pseudovibrio ascidiaceicola*, *Pseudovibrio denitrificans*, *Pseudovibrio japonicus*, *Pseudovibrio axinellae*, *Polycladidibacter stylochi*, *Polycladidibacter hongkongensis*, *Pseudovibrio flavus*, and *Pseudovibrio exalbescens*) (S1 Table), except for the type strain of *Pseudovibrio brasiliensis*, which was unavailable. All strains, including strains MaLMAid0302^T^ and SPO723^T^, showed no activity for urease, tryptophan deaminase, α-galactosidase, β-glucuronidase, α-mannosidase, and α-fucosidase but exhibited activity for alkaline phosphatase, esterase (C4), leucine arylamidase, and naphthol-AS-BI-phosphohydrolase. None of the strains metabolized L-arabinose, D-mannitol, capric acid, adipic acid, trisodium citrate, L-lysine, L-ornithine, sodium thiosulfate, inositol, D-sorbitol, L-rhamnose, or amygdalin, but all hydrolyzed esculin ferric citrate. Strain MaLMAid0302^T^ demonstrated clear distinctions from its closest relative, *Pseudovibrio flavus*, particularly in its inability to produce indole, reduce nitrate to nitrite, or exhibit N-acetyl-β-glucosaminidase activity. It standed out by displaying unique activities of esterase lipase (C8), trypsin, and acid phosphatase, differentiating it functionally from *P. flavus.* When compared to its closest relative *Pseudovibrio exalbescens*, strain SPO723^T^ showed notable differences in its metabolic profile. Strain SPO723^T^ metabolized L-arginine, gelatin, D-glucose, 4-nitrophenyl-β-D-galactopyranoside, D-mannose, N-acetyl-glucosamine, D-maltose, potassium gluconate, malic acid, phenylacetic acid, 2-nitrophenyl-β-D-galactopyranoside, sodium pyruvate, and gelatin, while notably failing to metabolize D-sucrose. Furthermore, it is distinguished by its unique enzymatic activities of esterase lipase (C8), lipase (C14), valine arylamidase, cysteine arylamidase, α-chymotrypsin, acid phosphatase, and β-galactosidase, setting it apart from *P. exalbescens*. These results suggest the novelty of strains MaLMAid0302^T^ and SPO723^T^ beyond the species level. In the previous 16S rRNA sequence-based phylogenetic analyses, a cluster comprising *Pseudovibrio denitrificans, Pseudovibrio japonicus, Pseudovibrio ascidiaceicola*, and *Pseudovibrio axinellae* consistently grouped with a cluster of *Polycladidibacter hongkongensis* and *Polycladidibacter stylochi* with over 90% bootstrap recovery across three methods (ML, MP, and NJ). However, no consistent phenotypic differences were observed between these clusters. This highlighted the need for a more refined and precise classification system beyond the current 16S rRNA-based framework. Chemotaxonomically, the fatty acid composition of the two strains was dominated by summed feature 8 (C18:1ω6c and/or C18:1ω7c), a characteristic common in the family *Stappiaceae* (S2 Table). Strain MaLMAid0302^T^ showed a higher diversity of fatty acids compared to *Pseudovibrio flavus*, with a notable difference in the higher proportion of C16:0 and a lower proportion of cyclo C19:0ω8c, C17:0. Some differences were also observed between strain SPO723^T^ and other *Pseudovibrio* species, particularly strain SPO723^T^ had the higher proportion of C17:0, C18:0, but lower proportion of cyclo C19:0ω8c than *P. exalbescens.* The distinctive fatty acid profile of *Pseudovibrio axinellae* further differentiated it from other *Pseudovibrio* species. These findings emphasized the need for continued taxonomic revisions, particularly for the family *Stappiaceae*, based on both phenotypic and chemotaxonomic data.

### Genome information and phylogenomic analysis

S3 Table provides a comprehensive list of 38 genomes collected for future analysis, including strains MaLMAid0302^T^ and SPO723^T^. In terms of genome characteristics, the size of the genomes ranged from 3.68 to 6.90 Mb, displaying a wide spectrum of genetic diversity. Additionally, the G + C content ratio exhibited considerable variation, spanning from 47.0 to 67.1%. The number of coding DNA sequences (CDSs) possessed by each genome also displayed considerable variability, ranging from a minimum of 3,349 to a maximum of 6,402. Although no unique genomic features were observed specific to each genus under the current classification system, the phylogenomic analysis revealed new trends worth exploring.

**Fig 2:**
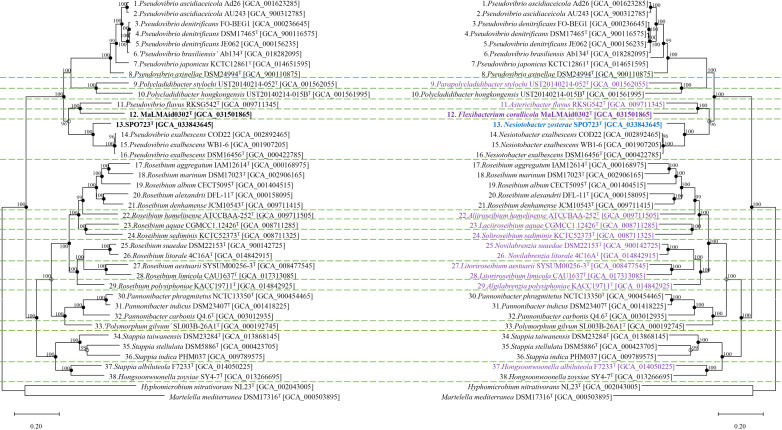
The mirror image of the phylogenomic tree of *Stappiaceae* members. The left tree represents the previous classification, while the right tree shows the revised classification. The tree was generated using PhyloPhlAn, with groups divided based on AAI values ranging from 71.92 to 72.88. In the right tree, species highlighted in purple represent those reclassified into new genera, while species highlighted in blue indicate newly classified species. Nodes recovered by both distance clustering trees (ANI and AAI scores) are marked with ●, while those recovered by a distance clustering tree are marked with ○.

The phylogenomic analysis conducted using the acquired genomes revealed the potential for dividing the data into at least 17 distinct groups (Fig 2). This grouping was further supported by the clustering tree based on distance matrices (S4 Fig). Notably, the clustering of *Stappia albiluteola* with *Hongsoonwoonella zoysiae*, rather than with other species of the genus *Stappia*, highlighted the necessity for reclassification and further investigation. Additionally, *Pannonibacter* and *Polymorphum* showed close proximity on the phylogenomic tree. To ascertain their accurate classification, a thorough comparison of index values was imperative. If *Polymorphum* and *Pannonibacter* are indeed determined to be distinct genera through further analysis, it could potentially result in the reorganization of numerous species within *Roseibium* and *Pseudovibrio* into new genera, as species within *Roseibium* and *Pseudovibrio* exhibited greater phylogenetic distances between each other than those observed between *Polymorphum* and *Pannonibacter*. Consequently, this reorganization could profoundly impact our understanding of diversity and relationships within the family *Stappiaceae*. Furthermore, under the current classification system, the genera *Pseudovibrio* and *Polycladidibacter* were intermixed, with strain MaLMAid0302^T^ forming a deep branch. Since these strains form distinct clusters, further in-depth analyses were necessary. Moreover, regarding the strains MaLMAid0302^T^ and SPO723^T^, while they appeared to belong to the existing *Pseudovibrio* group, a more in-depth examination was required due to the classification of the genus *Pseudovibrio* itself into at least four distinct clusters. If the classification is conducted based on the phylogenomic results, Group 1 exhibits a genome size ranging from 4.96 to 6.18 Mb, with a G + C content of 50.3 to 52.7% and 4,551–5,769 CDSs. In contrast, Group 2 displays a genome size of 3.68 Mb, a G + C content of 47.0%, and 3,349 CDSs, while Group 3 possesses a genome size of 3.75 Mb, a G + C content of 53.3%, and 3,534 CDSs. These distinct characteristics are consistently observed across all 17 groups delineated by phylogenomic analysis, demonstrating clear trends in genome features.

### Comparative analysis of genomic indices

ANI and *d*DDH comparisons among *Pseudovibrio ascidiaceicola* strains Ad26 and AU243 revealed that, despite belonging to the same species, their ANI values did not exceed the species delineation threshold of 95–96%, nor did their *d*DDH values surpass 70% (S4 and S5 Tables). Similarly, *Pseudovibrio denitrificans* strains FO-BEG1, DSM17465^T^, and JE062 also did not exceed these species classification thresholds. In contrast, the three strains of *Pseudovibrio exalbescens* (strains CDO22, WB1–6, and DSM16456^T^) exceeded the species-level criteria, with ANI values close to 100% and *d*DDH values above 80%, confirming that they belong to the same species. The ANI comparison of strain MaLMAid0302^T^ with *Pseudovibrio flavus* RKSG542^T^ revealed an ANI value of 71.31, markedly lower than the standard value for species classification. Similarly, the strain SPO723^T^ showed its highest ANI value with *Pseudovibrio exalbescens* strains, with a value of 76.13 ± 0.08, which is also much lower than the species delineation criteria. Having confirmed the novelty of strains MaLMAid0302^T^ and SPO723^T^ at the species level through the comparison of ANI and *d*DDH values, we further investigated whether they exhibited marked differences at the genus level by comparing AAI and POCP values (S6 and S7 Tables). To establish the optimal threshold for AAI, we performed repeated calculations based on the AAI results from Park *et al.,* 2022. The possible thresholds obtained were as follows: [60.68–60.78], [71.92–72.88], [75.90–76.69], [76.70–77.32], and [78.42–79.99]. Applying the lowest value [60.68–60.78] led to the grouping of 22 species from *Roseibium aggregatum* to *Hongsoonwoonella zoysiae* into one genus. However, this criterion encompassed five genera, including *Roseibium, Pannonibacter, Polymorphum, Stappia*, and *Hongsoonwoonella*, rendering it incorrect. On the other hand, applying the threshold [71.92–72.88] resulted in the division of a total of 17 groups. Interestingly, these 17 groups exactly matched the groups delineated in the previous phylogenomic analysis (Fig 2). Notably, *Stappia albiluteola* and *Hongsoonwoonella zoysiae* formed one group, indicating a need for internal reorganization within the genera *Stappia* and *Hongsoonwoonella*. The remaining species of the genus *Stappia* belonged to Group 16, those of the genus *Pannonibacter* to Group 14, and *Polymorphum gilvum* to Group 15, demonstrating results consistent with the existing classification system. Group 7 included some species, including the type species of *Roseibium*. *Roseibium hamelinense* was located in Group 8, *Roseibium aquae* in Group 9, and *Roseibium sediminis* in Group 10. These species formed a slightly distant cluster from the one formed by *Roseibium*, the close species of the type species, further supporting their reasonable division based on the phylogenomic tree. Members of the genus *Pseudovibrio* and *Polycladidibacter*, including strains MaLMAid0302^T^ and SPO723^T^, were classified into six distinct groups. *Pseudovibrio flavus* was assigned to Group 4, while strain MaLMAid0302^T^ formed Group 5. *Pseudovibrio ascidiaceicola*, *Pseudovibrio denitrificans*, *Pseudovibrio brasiliensis*, *Pseudovibrio japonicus*, and *Pseudovibrio axinellae* clustered together in another group. Meanwhile, strain SPO723^T^ grouped with *Pseudovibrio exalbescens*, and *Polycladidibacter stylochi* and *Polycladidibacter hongkongensis* were placed in separate clusters. Given that AAI thresholds higher than [71.92–72.88] resulted in excessive single-member groups in *Stappiaceae*, this threshold was considered optimal as it aligned with the results of phylogenomic analysis and provided a reasonable subdivision. We extended our investigation to assess whether the patterns observed in AAI comparisons were consistent when using POCP as another indicator for genus delineation. POCP employs a clear genus classification standard value of 50. However, we found that in most cases, the comparative values exceeded 50, and the individual genera were not distinctly separated. This inconsistency and lack of clear differentiation made POCP unsuitable as a reliable classification standard index for our study. This trend has also been observed in other studies, suggesting that setting thresholds using AAI could be the optimal standard for genus delineation. Ultimately, we established [71.92–72.88]% AAI as the genus classification threshold and reorganized *Stappiaceae* into 17 genera (*Pseudovibrio, Parapolycladidibacter, Polycladidibacter, Astericibacter, Flexibacterium, Nesiotobacter, Roseibium, Aliiroseibium, Laciiroseibium, Soliroseibium, Novilabrenzia, Litoriroseibium, Algilabrenzia, Pannonibacter, Polymorphum, Stappia,* and *Hongsoonwoonella*). The characteristics of these newly organized genera are described in Table 1.

**Table 1:** General characteristics of the genera in the family *Stappiaceae.*

Characteristic	1	2	3	4	5	6	7	8	9
Type species	*Pseudovibrio denitrificans*	*Parapolycladidibacter stylochi*	*Polycladidibacter hongkongensis*	*Astericibacter flavus*	*Flexibacterium corallicola*	*Nesiotobacter exalbescens*	*Roseibium denhamense*	*Aliiroseibium hamelinense*	*Laciiroseibium aquae*
Isolation source	Seawater, sponge, squirts	Flatworm	Flatworm	Sponge	Coral	Hypersaline lagoon, Deep sea, Zostera marina	Seawater, Sediments, marine organisms	Sands	Saline lake
Cell type	(Irregular) Rods	Rods	Long Rods	Rods, star-shaped	Straight to curved rods	Rods	Rods, aggregated	Rods	Rods
Colony color on Marine Agar	Beige to brownish green	Brownish white	Brownish white	Beige to light yellow	Beige to cream white	Beige to white	White to slightly pink	Pink, pale yellow	Pale pink
Motility	+	+	+	+	+	+	+	+	+
Respiration mode	Facultative anaerobic	Strictly aerobic	Facultative anaerobic	Facultative anaerobic	Strictly aerobic	Facultative anaerobic	Strictly aerobic, Facultative anaerobic	Strictly aerobic	Strictly aerobic
NO_3_- > NO_2_/NO_2_- > N_2_	v/v-	-/+	+/-	+/-	+/-	v/v	v+	+	+
Growth range									
Temperature (°C)^†^	10-35 (24-30)	15-40(25-28)	15-40(25-28)	22-37(22-30)	10-30(15-20)	4-45(30-33)	13-45 (25-30)	15-45(27-30)	20-40(35)
pH^†^	5-9(7-8)	6-9(7)	6-9(7)	7-9(7)	7-11(7)	6-9.5(7.5)	6-10(7-8)	7-10(7.5-8)	6.5-10.5(7.5-8)
NaCl (%, w/v)^†^	1-6 (3)	1-5(3)	1-5(3)	0.5-5(2)	1-6(3)	0.5-17.5(3-3.5)	0.5-11(3)	0-11.5	0-8(1-2)
Major Cell components									
Fatty acids (>5%)	SF8, cyclo C_19:0_ω8c, (SF3, C_16:0_, C_18:0_)	SF8, C_16:0,_ SF3	SF8, C_16:0,_ SF3	SF8, cyclo C_19:0_ω8c, C_18:0_	SF8, cyclo C_19:0_*ω*8c	SF8, (cyclo C_19:0_ω8c, C_18:0_)	SF8, C_20:1_ ω7c, C_18:0_, (C_18 : 1_ω7c 11-methyl)	SF8, C_20:1_ ω7c, C_18:0_, C_18 : 1_ω7c 11-methyl	SF8, C_18 : 1_ω7c 11-methyl, C_18:0_, C_20:1_ ω7c
Polar lipids	PE, PG, DPG, ALs, PLs, Ls	nd	nd	PE, PG, DPG, ALs, PLs, L	PE, PG, DPG, AL, PL, Ls	PE, PG. DPG, PLs, APL, (AL, L)	PG, DPG, PMME, PC, SQDG, AL, (PE, PL, L)	PG, DPG, PE, PMME, PC, SQDG, AL	DPG, PG, PE, PMME, SQDG, ALs, Ls
G + C content (%)	50.3-52.7	47	53.3	52.6	49.4	55.1-55.5	56.4-60.3	56.4	61
References	[6,48–51]	[52]	[53]	[54]	This study	This study, [55]	[7,8,56–58]	[7,59]	[60]
**Characteristic**	**10**	**11**	**12**	**13**	**14**	**15**	**16**	**17**	
Type species	*Soliroseibium sediminis*	*Novilabrenzia suaedae*	*Litoriroseibium aestuarii*	*Algilavrenzia polysiphoniae*	*Pannonibacter phragmitetus*	*‘Polymorphum gilvum’*	*Stappia stellulata*	*Hongsoonwoonella zoysiae*	
Isolation source	sediment	Halophyte, sediments	Freshwater, seawater, mud	Red algae	Reed, coal mine, hot spring sediments	Oil-contaminated saline soil	Coastal hot spring, marine mud, seawater	coastal sediments including rhizosphere	
Cell type	Rods	(short) Rods	Rods	Rods	Rods	Rods, star-shaped	short rods, star-shaped	(Short) rods	
Colony color on Marine Agar	Pale yellow	White to light brown	Cream to pale yellow	Light cream	Beige to Cream	Pale yellow	Beige	Pale beige to light brown	
Motility	+	–	+		+	+	+	v+	
Respiration mode	Facultative anaerobic	Strictly aerobic, Facultative anaerobic	Strictly aerobic	Aerobic	Facultative anaerobic, Strictly aerobic	Facultative anaerobic	Strictly aerobic, Facultative anaerobic	Facultative anaerobic, Strictly aerobic	
NO_3_- > NO_2_/NO_2_- > N_2_	+	v/-	–	–	v/+	-/-	v/+	v/v	
Growth range of:									
Temperature (°C)^†^	10-45(32)	10-40(28-30)	14-45(28-30)	7-39(28-30)	4-55(22-28/30-35)	4-50(37)	4-45(25-30/37)	4-42(28-30_	
pH^†^	5-8(6)	5-10(6-7/7-8)	4-10(6-8)	5.5-10(6.5-7.5)	3.5-11(6.5-8/9-10)	5-9(6)	6.5-11.5(7)	5.5-8.5(6.5-7)	
NaCl (%, w/v)^†^	1-7(4)	0-9(0-5)	0-8(2-3)	1-8(3-4)	0-7(0-2)	0-6(1)	0.5-11(1-5)/0.3-6(2-3)	0-1-6-9(1-3)	
Major Cell components									
Fatty acids (>5%)	SF8, SF2	SF8, (SF2, C_18:0_)	SF8, C_18 :1_ω7c 11-methyl, (C_17:0_, C_18:0_)	SF8, C_18 :1_ω7c 11-methyl, C_18:0_	SF8, (C_18 : 1_ω7c 11-methyl, C_18:0_,)	SF8, C_16:0_	SF8, C_18 :1_ω7c 11-methyl, (cyclo C_19:0_ω8c)	SF8, (SF3, C_18 :1_ω7c 11-methyl, C_16:0_, C_18:0,_ cyclo C_19:0_ω8c)	
Polar lipids	PC, PMME, PG, PE, AL, L	DPG, PE, PG, PC, PMME, (AL, PL, Ls)	PE, PG, PC, PMME, APL, PL	PE, PG, PC, PMME, APL, PL	DPG, PG, PE, PC, AL, (PMME, PLs, L)	DPG, PMME, PG, PC, AL, PLs, SQDG	DPG, PC, PG, PE, AL, APL, (GL, PL)	PC, PE, PG, APL, (DPG, AL, L)	
G + C content (%)	57	59.8-60.2	58.4-64.8	58.1	63.1-63.6	65.6	64.7-67.3	60.8-63.3	
References	[61]	[62,63]	[59,64]	[65]	[4,66,67]	[68]	[8,69,70]	[71,72]	

Genera are as follows; 1. *Pseudovibrio* (n = 5), 2. *Parapolycladidibacter* (n = 1), 3. *Polycladidibacter* (n = 1), 4. *Asteriscibacter* (n = 1), 5. *Flexibacterium* (n = 1), 6. *Nesiotobacter* (n = 2), 7. *Roseibium* (n = 5), 8. *Aliiroseibium* (n = 1), 9. *Laciiroseibium* (n = 1), 10. *Soliroseibium* (n = 1), 11. *Novilabrenzia* (n = 2), 12. *Litoriroseibium* (n = 2), 13. *Algilabrenzia* (n = 1), 14. *Pannonibacter* (n = 3), 15. *Polymorphum* (n = 1), 16. *Stappia* (n = 3), 17. *Hongsoonwoonella* (n = 2). Cells marked with the † symbol indicate the growth range, while the numbers in parentheses represent the optimum growth range. In the fatty acids data, values in parentheses indicate that some species within the genus exhibit amounts exceeding 5%, but this is not a universally shared characteristic among all species. Similarly, in the polar lipids data, values in parentheses indicate that certain species within the genus possess the feature, but it is not common to all species. Characters are scored as follows: (-) negative; (+) positive; (v) variable.

### Genome contents and comparative genomics of novel taxa

**Fig 3:**
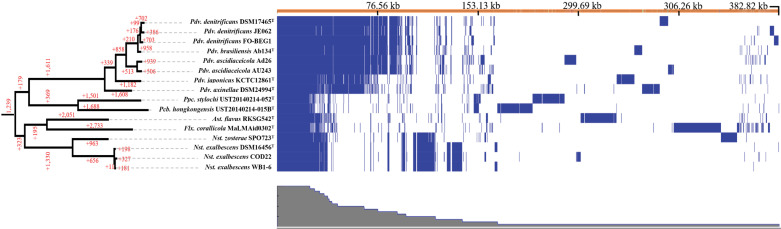
Distribution of CDS in genomes of strain MaLMAid0302^T^, strain SPO723^T^, and 14 related type strains. The top panel is the frequency of CDS among 16 genomes; the left panel is an AAI-based genomic tree with the number of CDS common in each branch; the bottom panel is the presence of CDS in each genome.

To conduct an in-depth analysis of the two novel microbial species we isolated, a pangenome analysis was performed using genomes from 16 closely related strains, belonging to the genus *Pseudovibrio, Parapolycladidibacter, Polycladidibacter, Astericibacter, Flexibacterium,* and *Nesiotobacter*. The purpose of this analysis is to determine whether the newly restructured taxa exhibit any distinct gene content patterns (Fig 3). A total of 19,139 coding domain sequence (CDS) clusters were identified through Roary analysis, using a clustering criterion of AAI > 71.92%. Among these, 1,239 clusters were shared across all 16 genomes, while the majority (12,083 clusters) consisted of singletons unique to individual genomes of the type strains. Additionally, 5,817 CDS clusters were variably present or absent depending on the taxa. The genus *Pseudovibrio* appeared to be taxonomically well-defined as the largest number of CDS clusters were conserved among its species-level type stains. The genus *Nesiotobacter* also showed a substantial number of genus-specific clusters. In addition, type strain genomes of the novel genera *Asteriscibacter* and *Flexibacterium* hardly shared CDS among themselves and with other genera. The two flat-worm dweller strains of *Polycaldidibacter* and *Parapolycladidibacter* also showed relatively fewer CDS clusters shared with other genera. The four strains belonging to *Asteriscibacter, Flexibacterium, Polycaldidibacter*, and *Parapolycladidibacter* carried more than 1,500 CDS clusters not found among their phylogenetic relatives and exhibited a long stretch of clusters unique only in the specific genomes (Fig 3). To identify genes specific for each of the novel taxa, sets of CDS clusters associated with the formation of terminal branches corresponding to *Asteriscibacter flavus* gen. nov., *Flevibacterium corallicola* gen. nov., sp. nov., *Nesiotobacter zosterae* sp. nov. were further annotated with KEGG modules (Table 2). Only a small fraction of more than 1,500 clusters could be identified as KEGG-annotated proteins. While genes related to carbohydrate and amino acid metabolism were notable for the *Asteriscibacter* genome, genes unique to the strain MaLMAid0302^T^ of *Flexibacterium* were diverse as encompassing nitrate metabolism (both assimilatory and dissimilatory), histidine degradation, and virulence factor (dTDP-L-rhamnose and aerobactin) biosynthesis. In the case of the strain SPO723^T^, which is *Nesiotobacter zosterae* sp. nov*.,* capacities of producing a virulence factor and coenzyme F420 were noted. The taxon-specific or genome-specific diversification of those taxa can be interpreted as a result of natural selection specific to their habitats.

**Table 2:** KEGG gene modules annotated among CDS appearing specifically in the genomes of novel taxa.

Taxa	KEGG Module	KEGG orthologs		
**Entry**	**E.C.**	**Name**
***Astericibacter flavus* gen. nov.**				
	M00631 D-Galacturonate degradation	K00041	1.1.1.58	tagaturonate reductase
		K00874	2.7.1.45	2-dehydro-3-deoxygluconokinase
		K01625	4.1.2.14	2-dehydro-3-deoxyphosphogluconate aldolase
		K01685	4.2.1.7	altronate hydrolase
		K01812	5.3.1.12	glucuronate isomerase
		K16850	4.2.1.7	altronate dehydratase large subunit
	M00061 D-Glucuronate degradation	K00040	1.1.1.57	fructuronate reductase
		K00874	2.7.1.45	2-dehydro-3-deoxygluconokinase
		K01625	4.1.2.14	2-dehydro-3-deoxyphosphogluconate aldolase
		K01686	4.2.1.8	mannonate dehydratase
		K01812	5.3.1.12	glucuronate isomerase
	M00948 Hydroxyproline degradation	K12658	5.1.1.8	4-hydroxyproline epimerase
		K13877	1.2.1.26	2,5-dioxopentanoate dehydrogenase
		K21062	3.5.4.22	1-pyrroline-4-hydroxy-2-carboxylate deaminase
***Flexibacterium corallicola* gen. nov., sp. nov.**				
	M00531 Assimilatory NO_3_^-^ reduction	K00372	1.7.99.-	assimilatory nitrate reductase catalytic subunit
		K26138	1.7.1.4	nitrite reductase [NAD(P)H] small subunit
		K26139	1.7.1.4	nitrite reductase [NAD(P)H] large subunit
	M00530 Dissimilatory NO_3_^-^ → NH_3_	K02567	1.9.6.1	nitrate reductase (cytochrome)
		K02568	–	nitrate reductase (cytochrome) electron transfer subunit
	M00529 Denitrification NO_3_^-^ → N_2_	K00376	1.7.2.4	nitrous-oxide reductase
		K02305	–	nitric oxide reductase subunit C
		K02567	1.9.6.1	nitrate reductase (cytochrome)
		K02568	–	nitrate reductase (cytochrome), electron transfer
		K04561	1.7.2.5	nitric oxide reductase subunit B
		K15864	1.7.2.1	nitrite reductase (NO-forming)
	M00615 NO_3_^-^ assimilation	K15576	–	nitrate/nitrite transport system substrate-binding
		K15577	–	nitrate/nitrite transport system permease protein
	M00045 Histidine degradation	K01458	3.5.1.68	N-formylglutamate deformylase
		K01468	3.5.2.7	Imidazolonepropionase
		K01712	4.2.1.49	urocanate hydratase
		K01745	4.3.1.3	histidine ammonia-lyase
		K05603	3.5.3.13	formimidoylglutamate deiminase
	M00793 dTDP-L-rhamnose biosynthesis	K00067	1.1.1.133	dTDP-4-dehydrorhamnose reductase
		K00973	2.7.7.24	glucose-1-phosphate thymidylyltransferase
		K01710	4.2.1.46	dTDP-glucose 4,6-dehydratase
		K01790	5.1.3.13	dTDP-4-dehydrorhamnose 3,5-epimerase
	M00918 Aerobactin biosynthesis	K03895	6.3.2.39	aerobactin synthase
		K03896	2.3.1.102	acetyl CoA:N6-hydroxylysine acetyl transferase
		K03897	1.14.13.59	lysine N6-hydroxylase
***Nesiotobacter zosterae* sp. nov.**				
	M00793 dTDP-L-rhamnose biosynthesis	K00067	1.1.1.133	dTDP-4-dehydrorhamnose reductase
		K00973	2.7.7.24	glucose-1-phosphate thymidylyltransferase
		K01710	4.2.1.46	dTDP-glucose 4,6-dehydratase
		K01790	5.1.3.13	dTDP-4-dehydrorhamnose 3,5-epimerase
	M00378 F420 biosynthesis archaea	K11212	2.7.8.28	LPPG:FO 2-phospho-L-lactate transferase
		K12234	6.3.2.31	coenzyme F420-0:L-glutamate ligase
		K14941	2.7.7.105	phosphoenolpyruvate guanylyltransferase

### Genome variability and habitat-driven functional analysis

**Fig 4:**
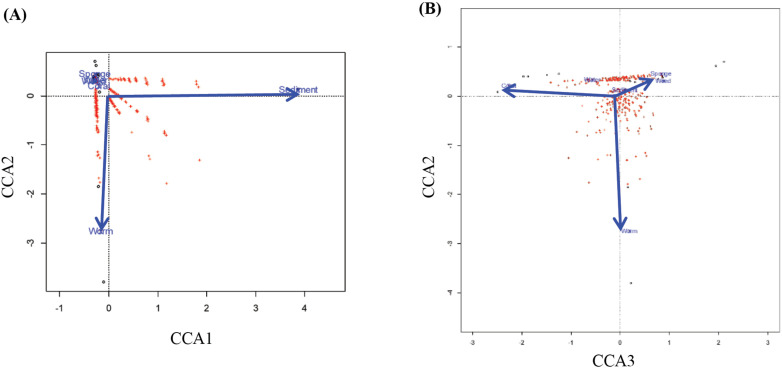
Canonical correspondence analysis (CCA) ordination diagram showing correlation among genomes, CDS, and habitat types. Genoms (○), CDS (+), and habitat types (arrows; weed = eelgrass, worm = flatworm, water = seawater, sediment = deep-sea sediment, coral = coral).

Notably, the majority of the family *Stappiaceae* originated from marine environments. The 38 strains derived from marine environments encompass sediment, water, and a diverse range of hosts, such as corals, marine sponges, flatworms, oysters, dinoflagellates, unicellular protists, and halophytes. Given the potential influence of habitat identified in the previous gene content analysis, we conducted canonical correspondence analysis (CCA) of the presence/absence of genes in the pangenome of the 16 genomes of closely related species, including strains MaLMAid0302^T^ and SPO723^T^. During this stage, we classified genes distinctively make their presence in a habitat-specific manner, (i.e., the presence of a gene in response to environmental forcing). The isolation sources of the 16 genomes could be classified into 6 kinds: sponge, coral, flatworm, seawater, sea sediment, and seagrass (Table 3). With CCA analysis, five axes were identified to explain 36% of the variability in the presence/absence of CDS at each of the six habitat types. This estimate implies that 64% of genome contents in the 16 bacteria were determined by their phylogeny, while 36% of genome contents were present due to environmental forcing. The first CCA axis was the strongest environmental forcing and explained 15% of the total CDS variability, and it separated the deep-sea sediment of the Indian Ocean from other habitats (Fig 4A). The second axis separated the flatworm habitat, which was represented by the two strains from marine flatworms in Hong Kong coastal waters, from the other five kinds of habitats, and it explains 8% of genome variance. (Fig 4A and B). Since genomes of the two flatworm strains diverged enough to form separate genera, genes present in both strains imply supra-generic phylogenetic forcing or the environmental forcing from the flatworm habitat and the geographical location of the Hong Kong Sea. Therefore, environmental forcing from deep-sea sediment of the Indian Ocean and flatworms in the Hongkong Sea appeared to determine 23% of CDS presence/absence. CDS differentially distributed between coral strain genomes and sponge strain genomes constituted the third axis factor in CCA, which explained 5.3% of total CDS variability. It should be noted that the estimate also includes the contribution of genome contents of the eelgrass strain *Nst. zosterae* SPO723^T^, which behaved the same way as those of sponge strains (Fig 4B). The genome contents of strains from the eelgrass habitat and sponge habitat were similar in spite that they are affiliated with 3 different genera or 6 different species. On the contrary, the genome contents of the two coral strains, which belong to two different genera, included some common features not found in those of eelgrass and sponge strains. A possible explanation for the similarity in genome contents of the eelgrass strains to those of sponge strains is that many sponges tend to have algal symbionts in their bodies [73–75]. While coral provides an animal host biosphere, the sponges might provide an algal host biosphere by which environmental forcing from plant physiology might contribute to the genomes of their bacterial commensals.

**Table 3:** Characteristics of strains whose genome contents were analyzed for environmental forcing.

Strain name	Accession No.	Location	Habitat type	Habitat description
*Astericibacter flavus* RKSG542^T^	GCA_009711345	Bahamas	sponge	*Verongula gigantea*
*Pseudovibrio denitrificans* JE062	GCA_000156235	Florida	sponge	*Mycale laxissima*
*Pseudovibrio brasiliensis* Ab134	GCA_018282095	Brazil	sponge	*Arenosclera brasiliensis*
*Pseudovibrio axinellae* DSM24994^T^	GCA_900110875	Ireland	sponge	*Axinella dissimilis*
*Pseudovibrio ascidiaceicola* Ad26	GCA_001623285	Ireland	sponge	*Axinella dissimilis*
*Pseudovibrio ascidiaceicola* AU243	GCA_900312785	Australia	sponge	*Cymbastela concentrica*
*Pseudovibrio denitrificans* FO-BEG1	GCA_000236645	Florida	coral	scleractinian coral, *Beggiatoa* symbiont
*Flexibacterium corallicola* MaLMAid0302^T^	GCA_031501865	Korea	coral	*Scleronephthya gracillimum*
*Parapolycladidibacter stylochi* UST20140214-052^T^	GCA_001562055	Hongkong	flatworm	*Stylochus* sp.
*Polycladidibacter hongkongensis* UST20140214-015B^T^	GCA_001561995	Hongkong	flatworm	*Stylochus* sp.
*Nesiotobacter zosterae* SPO723^T^	GCA_033843645	Micronesia	eelgrass	*Zostera marina*
*Pseudovibrio denitrificans* DSM17465^T^	GCA_900116575	Taiwan	seawater	shallow coastal water
*Pseudovibrio japonicus* KCTC12861^T^	GCA_014651595	Japan	seawater	coastal surface water
*Nesiotobacter exalbescens* DSM 16456 ^T^	GCA_000422785	Hawaii	seawater	hypersaline lagoon, thermophilic
*Nesiotobacter exalbescens* WB1–6	GCA_001907205	China	seawater	Yellow Sea
*Nesiotobacter exalbescens* COD22	GCA_002892465	India	sediment	2,100-m depth sediment

To identify CDS (coding sequences) specific to a habitat, CCA (Canonical Correspondence Analysis) scores for each CDS were analyzed. CDS positively correlated with the CCA1 axis (r = 0.99) were considered specific to the sediment habitat, while those negatively correlated with the CCA2 axis (r = -0.99) were deemed specific to the flatworm habitat. Interestingly, the CCA3 axis separated strains from corals from sponges. CDS negatively correlated with the CCA3 axis (r = -0.33) were treated as specific to the coral habitat, whereas positive values on the CCA3 (r = 0.65 to sponge, r = 0.25 to eelgrass) axis indicated specificity to the eelgrass-sponge habitat. Since CCA scores close to zero suggest neutrality to each axis, we selected CDS with deviations greater than 0.15 from zero for both positive and negative directions on each axis. CDS related to the central dogma, glycolysis, TCA cycles, and amino acid biosynthesis remained in the ± 0.15 score range for all axes. Among the collected CDS for each axis, fewer than 60% were annotated as known genes, with the remainder predicted to encode putative hypothetical proteins of unknown function. As summarized in the CCA columns of Table 4, which is based on the results shown in S8 Table, enrichment analysis identified metabolic pathways specific to sediment, flatworm, and sponge (cofounded with eelgrass) habitats. For sediment-specific CDS, pathways related to the degradation of typical substrates such as pectin and aromatic compounds were identified, along with anaerobic respiration pathways, including nitrogen reduction. In flatworm-specific collections, genes associated with nitrogen reduction and galactose metabolism were found, suggesting collaborative metabolism between the animal host and bacterial commensals under anaerobic conditions. For sponge-eelgrass CDS, degradation of aromatic compounds and galactose metabolism were noted, with flagella production emerging as a distinct genetic feature.

**Table 4:** Presence (○or Δ) of CDS clusters specific in bacterial genomes isolated from five kinds of habitats.

Function	Sediment		Flatworm		Sponge		Coral		Eelgrass	
	CCA	Taxa^*a*^	CCA	Taxa^*b*^	CCA	Taxa^*c*^	CCA	Taxa^*d*^	CCA	Taxa^*e*^
Pectin lysis	○									
Aromatic (styrene) degradation	○									
Aromatic (atrazine) degradation					○					
Nitrogen reduction	○		○							
Galactose metabolism			○		○					
Flagella production					○	Δ				
Sulfur metabolism								○		
Aerobactin biosynthesis (siderophore+virulence)						Δ				
Drug resistance (pump)						Δ				
GABA shunt (oxygen stress)						Δ				
CMP-Neu5Ac biosynthesis (Virulence)						Δ				
dTDP-L-rhamnose biosynthesis (virulence)										○

^a^ Specific to *Nst. exalbescense* COD22.

^b^ Common to *Parapolycladidibacter stylochi* UST20140214-052^T^ and *Polycladidibacter hongkongensis* UST20140214-015B^T^ but absent in strains from other habitats.

^c^ Found in one of the six genomes of bacteria isolated from sponges (*Pdv. denitrificans* JE062, *Pdv. brasiliensis* Ab134^T^, *Pdv. ascidiaceicola* Ad26, *Pdv. ascidiaceicola* AU243, *Pdv. axinellae* DSM24994^T^, and *Astericibacter flavus* RKSG542^T^) but absent in strains from other habitats.

^d^ Common to *Pdv. denitrificans* FO-BEG1 and *Flexibacterium corallicola* MaLMAid0302^T^ but absent in strains from other habitats.

^e^ Found only *Nesiotobacter zosterae* SPO723^T^.

In addition to CCA, an alternative approach to identifying habitat-specific genes involved isolating CDS unique to bacteria from a given habitat. We applied this method by collecting CDS present exclusively in isolates from a specific habitat and absent in bacteria from other habitats, subsequently annotating the CDS into KEGG orthologs (Table 4 – Taxa columns). Among the habitats of deep-sea sediment, flatworm, sponge, coral, and eelgrass, only two yielded annotated CDS sets using this method. Coral strains carried genes linked to sulfur metabolism, indicating potential reliance on bacterial sulfur metabolism for inorganic material processing. Notably, a single eelgrass strain possessed genes for synthesizing dTDP-L-rhamnose, potentially enhancing biofilm formation and adhesion to eelgrass surfaces. For sponge isolates, no genes were uniquely common across all strains and absent in others. Previous studies suggest that sponge symbionts are influenced more by host species-specific factors than biogeographical variability. The absence of sponge-specific genes may result from the immense diversity of sponge strains, given their isolation from different sponge species across various global locations [76–78]. Therefore, we identified genes present in any sponge strain but absent in all other habitats (Table 4 – triangles). This approach revealed five metabolic capacities unique to sponge strains, including flagella production, drug resistance, oxygen stress response, and protein glycosylation. These findings suggest that sponge strains possess host-specific genetic adaptations.

## Conclusion and discussion

The two strains, MaLMAid0302^T^ and SPO723^T^, obtained from coral and eelgrass, were distinguished from the type species of the family *Stappiaceae* by their phenotypic, biochemical, phylogenetic, and chemotaxonomic characteristics. Based on their novelty, they should be reported as new species or genera. However, due to unresolved taxonomic issues within the existing *Stappiaceae* classifications, determining the taxonomic positions of these strains was challenging. Specifically, although their closest relatives, *Pseudovibrio flavus* and *Pseudovibrio exalbescens*, are classified within the genus *Pseudovibrio*, phylogenetic analyses revealed that *Polycladidibacter* species cluster within the same clade as *Pseudovibrio* species. This suggests that their closest relatives may also require reclassification into a new taxonomic group rather than remaining within the genus *Pseudovibrio*. The 16S rRNA gene has played a crucial role in microbial identification and remains a widely used marker in laboratories due to its convenience and high accuracy in species differentiation. However, its effectiveness is limited by intragenomic variation and reduced accuracy at higher taxonomic ranks, beyond the species level, highlighting the theory of ribosomal constraint [79–81]. To overcome these limitations, the rapid expansion of genomic databases, driven by advances in next-generation sequencing (NGS), has facilitated the widespread adoption of taxogenomics, which utilizes whole-genome data for classification. This is because genomes contain extensive genetic and evolutionary information that cannot be captured within the short length of the 16S rRNA gene. As a result, taxogenomics has helped resolve taxonomic challenges that could not be addressed solely through 16S rRNA-based phylogenetic analysis [82–84]. Therefore, we attempted to reclassify the *Stappiaceae* using taxogenomics and a method for deriving optimal AAI thresholds through repetitive clustering and evaluation.

Notably, strains of *Pseudovibrio ascidiaceicola* (strains Ad26 and AU243) did not meet the ANI and *d*DDH thresholds for classification as the same species. Their values fell below the species demarcation criteria, though they remained close to the threshold, leading to some ambiguity in their classification. Similarly, strains of *Pseudovibrio denitrificans* (FO-BEG1, DSM17465, and JE062) also did not exceed these species classification thresholds, resulting in a comparable classification challenge. While these strains could potentially be recognized as separate species, further taxonomic resolution was beyond the scope of this study. This limitation primarily arises from the requirement under the International Code of Nomenclature of Prokaryotes (ICNP) that new species must be deposited in at least two recognized culture collections. Some strains that could potentially be recognized as distinct species have not been deposited in multiple culture collections, and thus, species-level reclassification was not addressed in this study. Based on the results obtained from both AAI and POCP comparisons, it is evident that AAI offers more robust and meaningful insights for the delineation of genera. Although POCP is an important reference index for genus classification, recent studies have highlighted its limitations in providing clear reference values for certain taxa [84]. As a result, we have prioritized AAI as the primary indicator to establish genus-level distinctions for the strains MaLMAid0302^T^ and SPO723^T^ within the family *Stappiaceae*. The AAI index value for genus delineation ranges from 60% to 80%, resulting in ambiguity. However, through iterative calculation methods and considering the results of phylogenomic analysis, this paper proposes the range of [71.92–72.88] as the optimal threshold for classifying the genus within the family *Stappiaceae*. It is important to note that although this study utilized 38 genomes, the specificity of the AAI threshold is expected to improve with the inclusion of more genomes belonging to the family *Stappiaceae* in future research. As a result of the division into 17 groups according to the proposed optimal AAI threshold, substantial reclassification and modification of existing genera and species within the family *Stappiaceae* were observed. *Stappia albiluteola* was reclassified as *Hongsoonwoonella albiluteola*. Moreover, based on this classification, several new genera were proposed, including *Aliiroseibium*, *Laciiroseibium*, *Soliroseibium*, *Litoriroseibium*, *Algilabrenzia*, and *Novilabrenzia*, for species belonging to the genus *Roseibium*. Within the genus *Pseudovibrio, Polycladidibacter*, several species requiring reorganization were modified to form new genera, such as *Nesiotobacter*, *Astericibacter*, and *Parapolycladidibacter*. This extensive reclassification did not violate the monophyly rule for almost all species within the 16S rRNA-based ML tree (S5 Fig). However, *Roseibium denhamense* clustered with *Soliroseibium sediminis*, which conflicts with the results from phylogenomic analysis. When the 16S rRNA of *Roseibium denhamense* JCM10543^T^ was mapped to the NCBI nr database, *Aliiroseibium hamelinense* and *Laciiroseibium aquae* were identified as its closest relatives. However, when considering the ANI and AAI values from whole-genome comparisons, *Roseibium denhamense* is genetically closest to other *Roseibium* species. Such cases, where 16S rRNA does not accurately reflect evolutionary distance, are frequently observed in other studies [85]. Additionally, the bootstrap values from the 1,000 iterations performed for each cluster in the ML tree were very low compared to the robust bootstrap values observed in phylogenomic trees. This further underscores the need for reclassification across prokaryotic taxa using taxogenomics in the future. Furthermore, based on the findings of this study, strain MaLMAid0302^T^ was proposed as a type species of *Flexibacterium* gen. nov. The genome data used for this analysis were collected in late 2023. During the course of the analysis and manuscript preparation, three validly published species (*Roseibium algae*, *Roseibium algicola*, *Roseibium porphyridii*) and three not validly published species (*‘Pannonibacter anstelovis’, ‘Pannonibacter tanglangensis’, ‘Roseibium sediminicola’*) were proposed. Although their reclassification was not addressed in this study, a comparison of their AAI values with those of closely related species (S9 Table) indicated that most species exceeded the AAI threshold for the family *Stappiaceae*, suggesting that they could be classified within the same genus as their closest relatives. However, *Roseibium algae* exhibited an AAI value of 77.67 with its closest relative, *Algilabrenzia polysiphoniae*, implying a potential reclassification as *Algilabrenzia algae*.

Not only did we achieve successful reclassification, but we also expanded our focus to include the phylogenetic and environmental forcing impacts on our two strains, MaLMAid0302^T^ and SPO723^T^, which originated from marine hosts, and their closely related strains, which also originated from marine environments and marine organisms. The results from applying Canonical Correspondence Analysis (CCA) showed that 64% of the genetic composition could be explained by phylogenetic forcing, while the remaining 36% could be explained by environmental forcing. This suggests that considerable portions of the genetic composition of *Pseudovibrio* and its related strains within the family *Stappiaceae* are determined by their habitat. By identifying habitat-specific CDSs and conducting enrichment analysis, we found metabolic pathways of the strains, isolated from sediments, are the degradation of substrates abundant in sediments, such as pectin and aromatic compounds, and anaerobic respiratory pathways associated with nitrogen reduction. For strains derived from flatworms, common findings included nitrogen reduction and galactose metabolism, suggesting that the symbiotic microorganisms in flatworms may engage in cooperative metabolism under anaerobic conditions to utilize substrates. In the case of strains derived from coral, sulfur metabolism genes were specifically found, supporting the evidence, which is the symbiotic relationship between corals and sulfate-reducing bacteria [86]. Our findings suggest that strains inhabiting corals within the family *Stappiaceae* may acquire genes and engage in sulfur metabolism via environmental forcing, thereby contributing to the host’s health. The strain derived from eelgrass displayed an interesting result, as it possesses the ability to synthesize dTDP-L-rhamnose, which may enable the production of a biofilm rich in dTDP-L-rhamnose. This biofilm could enhance adhesion to the surface of the seaweed, allowing the strain to thrive in environments with strong currents and various environmental stresses [87].

It should be noted that the genome-to-genome differences observed due to environmental forcing were not solely a result of differences between habitats but also stemmed from various factors, including the geographical location of the samples, which ranged from the coast of Ireland to the Indian Ocean, a lagoon in Micronesia, and sponges from the Bahamas and Florida. In our study, three kinds of habitats (deep-sea sediment, flatworm, and eelgrass) were represented by only one isolation source. Therefore, caution is required in interpreting gene presence as it may be confounded by both geographical differences and habitat types. These limitations will likely be addressed in future studies as additional *Stappiaceae* members belonging to the *Pseudovibrio*-*Parapolycladidibacter*-*Polycladidibacter*-*Astericibacter*-*Flexibacterium*-*Nesiotobacter* cluster are reported, and the analysis sample size increases.

### Taxonomic conclusion

#### Description of *Parapolycladidibacter* gen. nov.

*Parapolycladidibacter* (Pa.ra.po.ly.cla.di.di.bac’ter. Gr. prep. *para*, next to; N.L. masc. n. *Polycladidibacter*, a bacterial genus; N.L. masc. n. *Parapolycladidibacter*, a genus adjacent to the genus *Polycladidibacter*)

The cells are Gram-stain positive, non-motile rod-shaped, chemo-organotrophic, and obligate anaerobic. Type species growing optimally at 37 °C and tolerate up to 6.5% NaCl. Major fermentation products of PYG (peptone-yeast extract-glucose) medium are acetic acid, isobutyric acid, isovaleric acid, and isocarpoic acid. DNA G + C ratio of strains is around 38%. The type species is *Parapolycladidibacter stylochi*.

#### Description of *Parapolycladidibacter stylochi* comb. nov.

*Parapolycladidibacter stylochi* (sty.lo’chi. N.L. gen. masc. n. *stylochi*, of the flatworm *Stylochus*)

Basonym: *Pseudovibrio stylochi* Zhang *et al.* 2016

#### Synonym: *Polycladidibacter stylochi* (Zhang *et al*. 2016) Hinger *et al*. 2024.

The description is the same as for *Polycladidibacter stylochi* (Zhang *et al.* 2016) Hinger *et al*., 2024.

The type strain UST20140214-052^T^ (=KCTC 42384^T^ = MCCC 1K00452^T^) was isolated from marine flatworm. The 16S rRNA rRNA gene and genome accession numbers are KP207600 and LLWE00000000, respectively.

#### Description of *Asteriscibacter* gen. nov.

*Asteriscibacter* (As.te.ris.ci.bac’ter. L. masc. n. asteriscus, small star or asterisk mark; N.L. masc. n. bacter, a rod; N.L. masc. n. *Asteriscibacter*, bacterium that forms star-shaped aggregates)

The cells are Gram-stain negative, motile rod-shaped, and sometimes cells make star-shaped aggregates. Mesophilic, neutrophilic, slightly halophilic and chemo-organotrophic. Cells are facultatively anaerobic and reduce nitrate to nitrite. Major fatty acids are C_18:0_, C_19:0_ ω8c cyclo, and summed feature 8 (C_18:1_ *ω*7c/C_18:1_ *ω*6c). Type species contain phosphatidyl ethanolamine, phosphatidyl glycerol, diphosphatidyl glycerol, unidentified amino-polar lipids, unidentified phospholipid and an unidentified polar lipid as cellular phospholipid. The DNA G + C content is around 52%. The type species is *Asteriscibacter flavus*.

#### Description of *Asteriscibacter flavus* comb. nov.

*Asteriscibacter flavus* (fla’vus. L. masc. adj. *flavus*, pale yellow)

Basonym: *Pseudovibrio flavus* Goldberg *et al.* 2022

The description is the same as for *Pseudovibrio flavus* Goldberg *et al.* 2022.

The type strain TSD-76^T^ (=ATCC TSD-76^T^ = LMG 29867^T^) was isolated from the sea sponge *Verongula gigante*. The 16S rRNA and genome accession number are KX896701 and SMMA00000000, respectively.

#### Description of *Flexibacterium* gen. nov.

*Flexibacterium* (Fle.xi.bac.te’ri.um. L. masc. perf. part. *flexus*, bent, winding; from L. v. *flecto*, to bend, to curve; N.L. neut. n. bacterium, a rod; N.L. neut. n. *Flexibacterium*, a flexible rod).

This novel genus is suggested based on phylogenetic and phylogenomic data. Cells are aerobic, Gram-stain negative, motile, and straight to curved rods. Cells are chemoorganotrophic, mesophilic, neutrophilic, and slightly halophilic. The colony color is beige to creamy white after 2 days of cultivation on Marine Agar 2216. Oxidase and catalase activities are positive and nitrate is reduced to nitrite but not to nitrogen gas. Major cellular fatty acids are summed feature 8 (C_18:1_*ω*6*c* and/or C_18:1_*ω*7*c*) and cyclo C_19:0_*ω*8*c.* The DNA G + C ratio is around 49%. The type species is *Flexibacterium corallicola*.

#### Description of *Flexibacterium corallicola* sp. nov.

*Flexibacterium corallicola* (co.ral.li’co.la. L. neut. n. *corallum*, coral; L. masc. suff. *-cola*, inhabitant dweller; N.L. n. *corallicola*, coral-dweller)

The cells are Gram-stain negative, aerobic, straight to curved rod shape (0.8–1.4 μm width and 1.3–5.6 μm length). The ranges and optimal conditions of temperature, pH, and required NaCl concentration for the growth on Marine broth 2216 medium were 10–30 °C (optimum, 25 °C), pH 7.0–11.0 (optimum, pH 7.0), and 1–6.0% (optimum, 3.0%), respectively. Catalase and oxidase activities are positive. Nitrate is reduced to nitrite. Enzyme activities of acid- and alkaline-phosphatases, esterase, esterase lipase, leucine arylamidase, trypsin, naphthol-AS-BI phosphohydrolase, and alpha-glucosidase are present when assayed with API ZYM system. Cellular fatty acids (>1%) are C_16:0_, C_18:0_, C_18:0_ 3-OH, cyclo C_19:0_ ω8c, and summed feature 8 (C_18:1_ *ω*7c/C_18:1_ *ω*6c). The DNA G + C contents of the type strain is 49.4%.

The type strain MaLMAid0302^T^ (=KCTC 92348^T^ = JCM 35474^T ^= M1P-2–3 ^T^) was isolated from substratum part of a coral species *Scleronephthya gracillimum* taken from south coast of Jeju Island, Republic of Korea (33^O^13’40” N, 126 ^O^34’07” E). Accession numbers of DDBJ/EMBL/NCBI for the 16S rRNA gene and whole genome sequences are OP606461 and JARRCW00000000.1, respectively.

#### Emended description of *Nesiotobacter.*

*Nesiotobacter* (Ne.si’o.to.bac’ter. Gr. masc. adj. *nêsiôtikos*, of an island, insular; N.L. masc. n. *bacter*, rod; from Gr. neut. dim. n. *baktêrion*, rod; N.L. masc. n. *Nesiotobacter*, rod from an island).

The cells are Gram-stain negative, motile rod-shaped, chemo-organotrophic, and facultatively anaerobic. Oxidase and catalase activities are present. Nitrate reduction is variable. Cells are mesophilic, neutrophilic, and slightly halophilic. The type species tolerates up to 17.5% NaCl. Common cellular fatty acids (>1%) are C_16:0_, C_18:0_, C_18:1_ ω7c 11-methyl, C_19:0_ ω8c cyclo, C_18:0_ 3-OH, Summed feature2 (C_14:0_ 3-OH and/or iso-C_16:1_) and summed feature 8 (C_18:1_ *ω*7c/C_18:1_ *ω*6c). Type species contain phosphatidyl ethanolamine, phosphatidyl glycerol, diphosphatidyl glycerol, an unidentified amino-polar lipid, and some unidentified polar lipids as major cellular phospholipid components. The DNA G + C content is around 55%. The type species is *Nesiotobacter exsalbescens*.

#### Emended description of *Nesiotobacter exsalbescens.*

*Nesiotobacter exsalbescens* (ex.al.bes’cens. L. part. adj. *exalbescens*, becoming white, growing white, referring to the fading color of maturing colonies; from L. v. *exalbesco*).

#### Synonym: *Pseudovibrio exalbescens* (Donachie *et al.* 2006) Hördt *et al*. 2020.

The description is the same as for *Pseudovibrio exalbescens* (Donachie *et al.* 2006) Hördt *et al.* 2020.

The type strain LA33B^T^ (=ATCC BAA-994^T^ = CIP 108449^T^ = DSM 16456^T^) was isolated from a hypersaline lagoon on Laysan Atoll in the Northwestern Hawaiian Islands. The 16S rRNA rRNA gene and genome accession numbers are AF513441 and AUGS00000000, respectively.

#### Description of *Nesiotobacter zosterae* sp. nov.

*Nesiotobacter zosterae* (zos′te.rae. N.L. gen. n. *zosterae* denoting that the bacterium was isolated from the seaweed *Zostera marina*).

Cells are facultative anaerobic, motile, Gram-stain negative rods (1.2–2.2 ㎛ × 0.4–0.5 ㎛). They lack flagella and occur either singly or in chains. Colonies are circular, convex, opaque, and butyrous with entire edges, 1–2 mm in diameter on marine agar after cultivation for 2–3 days. Growth was observed at temperatures between 15 and 45 °C (optimum 33 °C), pH between 6 and 9.5 (optimum 7.5), and presence of 0.5–10% (w/v, optimum 3–3.5%) NaCl. Produces oxidase and catalase. When assayed with the API ZYM system alkaline phosphatase, esterase (C4), esterase lipase (C8), lipase (C14), leucine arylamidase, Trypsin, α-chymotrypsin, α-glucosidase, β-galactosidase, and N-acetyl-β-glucosaminidase are present and valine arylamidase activity is weakly positive. But cystine arylamidase, acid phosphatase, naphthol-AS-BI-phosphohydrolase, α-galactosidase, β-glucuronidase, β-glucosidase, α-mannosidase and α-fucosidase are absent. Degrades N-acetyl-D-glucosamine, cellobiose, α-D-glucose, D-mannose, β-methyl-D-glucoside, methyl pyruvate, mono-methyl succinate, D-gluconic acid, α-keto glutaric acid, D, L-lactic acid, propionic acid, L-alanine, L-alanyl-glycine, L-glutamic acid, L-proline, uridine and thymidine and weekly utilize α-D-lactose, L-asparagine and L-serine on Microlog GN2 plates. Major fatty acids (>1%) are C_16:0_, C_17:0,_ C_18:0_, C_18:1_ ω7c 11-methyl, C_19:0_ ω8c cyclo, C_18:0_ 3-OH, Summed feature2 (C_14:0_ 3-OH and/or iso-C_16:1_) and summed feature 8 (C_18:1_ *ω*7c/C_18:1_ *ω*6c). The major respiratory quinone is Q-10. The DNA G + C content of DNA is 55.5%.

The type strain is SPO723^T^ (=KCCM42324 ^T^ = JCM 14066^T^), isolated from a leaf of *Zostera marina* near Weno Island, Chuuk State, Federated State of Micronesia. The 16S rRNA rRNA gene and genome accession numbers are DQ660386 and JAWXVZ000000000.1, respectively.

#### Description of *Aliiroseibium* gen. nov.

*Aliiroseibium* (A.li.i.ro.se.i.bi.um. L. pronoun *alius* other, another; N.L. neut. n. *Roseibium*, a bacterial genus name; N.L. masc. n. *Aliiroseibium*, another *Roseibium*)

The cells are Gram-stain negative, motile rod, and strictly aerobic. Cells are mesophilic, slightly alkalophilic, halotolerant, and chemo-organotrophic. Reduces nitrate to nitrogen gas. Major fatty acids (>5%) are C_18:0_, C_19:0_ ω8c cyclo, and summed feature 8 (C_18:1_ *ω*7c/C_18:1_ *ω*6c). The type species contained phosphatidyl ethanolamine, phosphatidyl glycerol, diphosphatidyl glycerol, phosphatidyl monomethyl ethanolamine, phosphatidylcholine, sulphoquinovosyldiacylglyceride and an unidentified amino-polar lipid as cellular phospholipid. The DNA G + C content is around 56%. The type species is *Aliiroseibium hameliense*.

#### Description of *Aliiroseibium hameliense* comb. nov.

*Aliiroseibium hameliense* (ha.me.li’nen.se. N.L. neut. adj. *hamelinense*, referring to Hamelin Pool, Australia, the source of the type strain).

#### Basonym: *Roseibium hamelinense* Suzuki *et al.* 2000.

The description is the same as for *Roseibium hamelinense* Suzuki *et al.* 2000, emend. Biebl *et al*. 2007.

The type strain OCh368^T^ (=ATCC BAA-252 ^T^ = CIP 107048^T^ = DSM 15090^T^ = IFO 16783^T^ = JCM 10544^T^ = NBRC 16783^T^) was isolated from coastal water. The 16S rRNA and genome accession numbers are D85836 and VLLF00000000, respectively.

#### Description of *Laciiroseibium* gen. nov.

*Laciiroseibium* (La.ci.ro.se.i’bi.um. L. masc. n. lacus, lake; N.L. neut. n. *Roseibium*, a bacterial genus name; N.L. neut. n. *Laciroseibium*, a relative of *Roseibium* from a lake)

The cells are Gram-stain negative, motile rod, and aerobic. Cells are mesophilic, neutrophilic, halotolerant, and chemo-organotrophic. Able to reduce nitrate. Cellular fatty acids (>5%) are C_18:0_, C_18:1_ ω7c 11-methyl, C_208:1_ ω7c and summed feature 8 (C_18:1_ *ω*7c/C_18:1_ *ω*6c). Type species contained phosphatidyl ethanolamine, phosphatidyl choline, phosphatidyl glycerol, diphosphatidyl glycerol, phosphatidyl monomethyl ethanolamine, sulphoquinovosyldiacylglyceride, 2 unidentified amino lipids and three unidentified pospholipids as polar lipids. The DNA G + C content is around 61%. The type species is *Laciiroseibium aquae*.

#### Description of *Laciiroseibium aquae* comb. nov.

*Laciiroseibium aquae* (a’quae. L. gen. fem. n. *aquae*, of water)

#### Basonym: *Roseibium aquae* Zhong *et al.* 2014.

The description is the same as for *Roseibium aquae* Zhong *et al.* 2014.

The type strain DSG-S4-2^T^ (=CGMSS 1.12426^T^ = JCM 19310^T^) was isolated from a saline lake. The 16S rRNA gene sequence and genome accession number are KC762314 and BMFA00000000, respectively.

#### Description of *Soliroseibium* gen. nov.

*Soliroseibium* (So.li.ro.se.i’bi.um. L. neut. n. *solum*, soil; L. masc. adj. *roseus*, rose, pink; Gr. masc. n. bios, life; N.L. neut. n. *Soliroseibium*, pink life in soil)

The cells are Gram-stain negative, motile rod, and facultatively anaerobic. Cells are mesophilic, slightly acidophilic, slightly halophilic, and chemo-organotrophic. Able to reduce nitrate to nitrogen gas. Major fatty acids (>5%) are summed feature 2 (C_14:0_ 3-OH and/or iso-C_16:1_ I) and summed feature 8 (C_18:1_ *ω*7c/C_18:1_ *ω*6c). The type species contained phosphatidyl ethanolamine, phosphatidyl glycerol, phosphatidyl monomethyl ethanolamine, phosphatidylcholine, an unidentified amino lipid, and an unidentified polar lipid as cellular phospholipids. The DNA G + C content is around 57%. The type species is *Soliroseibium sediminis*.

#### Description of *Soliroseibium sediminis* comb. nov.

*Soliroseibium sediminis* (se.di’mi.nis. L. gen. neut. n. *sediminis*, of sediment).

#### Basonym: *Roseibium sediminis* Liu *et al.* 2017.

The description is the same as for *Roseibium sediminis* Liu *et al.* 2017.

The type strain BSS09^T^ (=KCTC 52373^T^ = MCCC 1K3201^T^) was isolated from sea surface sediment. The 16S rRNA gene sequence and genome accession number are KU321206 and VXDB00000000, respectively.

#### Description of *Novilabrenzia* gen. nov.

*Novilabrenzia* (No.vi.la.bren’zi.a. L. masc. adj. *novus*, new; N.L. fem. n. *Labrenzia*, a genus name from the name Labrenz, honouring Dr Matthias Labrenz, a German marine microbiologist; N.L. fem. n. *Novilabrenzia*, a new *Labrenzia*).

The cells are Gram-stain negative, motile rod, and aerobic to facultative anaerobic. Cells are mesophilic, neutrophilic, halotolerant, and chemo-organotrophic. Could not reduce nitrate. Common cellular fatty acid (>5%) is summed feature 8 (C_18:1_ *ω*7c/C_18:1_ *ω*6c), and summed feature 2 (C14:0 3-OH and/or iso-C16:1 I) or C18:0 showed a high proportion depends on species. Cells contained phosphatidyl ethanolamine, phosphatidyl choline, phosphatidyl glycerol, diphosphatidyl glycerol, and phosphatidyl monomethyl ethanolamine as common components. The DNA G + C content is around 60%. The type species is *Novilabrenzia suaedae*.

#### Description of *Novilabrenzia suaedae* comb. nov.

*Novilabrenzia suaeda* (su.ae’dae. N.L. gen. n. *suaedae*, of Suaeda, referring to the isolation of the type strain from roots of *Suaeda maritima*)

#### Basonym: Labrenzia suaedae Bibi et al. 2014.

Synonym: Roseibium suaedae (Bibi et al. 2014) Hördt et al. 2020.

The description is the same as for Roseibium suaedae (Bibi et al. 2014) Hördt et al. 2020.

The type strain YC6927^T^ (=KACC 13772^T^ = DSM 22153^T^) was isolated from a halophyte. The 16S rRNA gene sequence and genome accession number are GU322907 and FRBW00000000, respectively.

#### Description of *Novilabrenzia litoralis* comb. nov.

*Novilabrenzia* *litoralis* ((li.to.ra’lis. L. fem. adj. *litoralis*, of or belonging to the seashore) Basonym: *Roseibium litorale* Liu *et al.* 2021

The description is the same as for *Roseibium litorale* Liu *et al.* 2021.

The type strain 4C16A^T^ (=KACC 22078^T^ = GDMCC 1.1932^T^) was isolated from tidal flat sediment. The 16S rRNA gene sequence and genome accession number are MW045200 and JACYXI00000000, respectively.

#### Description of *Litoriroseibium* gen. nov.

*Litoriroseibium* (Li.to.ri.ro.se.i’bi.um. L. neut. n. litus, shore; N.L. neut. n. *Roseibium*, a bacterial genus name; N.L. neut. n. *Litoriroseibium*, a relative of *Roseibium* from the shore)

The cells are Gram-stain negative, motile rod, and aerobic. Cells are mesophilic, neutrophilic, halotolerant, and chemo-organotrophic. Could not reduce nitrate. Common fatty acids (>5%) are C_18:1_ ω7c 11-methyl and summed feature 8 (C_18:1_ *ω*7c/C_18:1_ *ω*6c). Contained phosphatidyl ethanolamine, phosphatidyl glycerol, phosphatidyl monomethyl ethanolamine, phosphatidylcholine, an unidentified amino lipid, and an unidentified pospholipid as polar lipids. The DNA G + C content is ranged 58–65%. The type species is *Litoriroseibium aestuarii*.

#### Description of *Litoriroseibium aestuarii* comb. nov.

*Litoriroseibium aestuarii* (aes.tu.a’ri.i. L. gen. neut. n. *aestuarii*, of the estuary, the environment from which the type strain was isolated)

#### Basonym: *Roseibium aestuarii* Duan *et al.* 2020.

The description is the same as for *Roseibium aestuarii* Duan *et al.* 2020.

The type strain SYSU M00256-3^T^ (=CGMXX 1.16156^T^ = NBRC 112946^T^) was isolated from estuarine sediment. The 16S rRNA gene sequence and genome accession number are MN513348 and VORA00000000, respectively.

#### Description of *Litoriroseibium limicola* comb. nov.

*Litoriroseibium limicola* (li.mi’co.la. L. masc. n. *limus*, mud; L. suff. –*cola* from L. masc. or fem. n. *incola* dweller; N.L. fem. n. *limicola* mud-dweller)

#### Basonym: *Roseibium limicola* Weerawongwiwat *et al.* 2021.

The description is the same as for *Roseibium limicola* Weerawongwiwat *et al.* 2021.

The type strain CAU 1637^T^ (=KCTC 82429^T^ = MCCC 1K06080^T^) was isolated from tidal mudflat. The 16S rRNA gene sequence and genome accession number are MW131340 and JAFLNF00000000, respectively.

#### Description of *Algilabrenzia* gen. nov.

*Algilabrenzia* (Al.gi.la.bren’zi.a. L. fem. n. *alga*, a seaweed; N.L. fem. n. *Labrenzia*, a genus name honouring Dr Matthias Labrenz, a German marine microbiologist; N.L. fem. n. *Algilabrenzia*, a relative of *Labrenzia* from an alga)

The cells are Gram-stain negative, motile rod, and aerobic. Cells are mesophilic, slightly acidophilic, slightly halophilic, and chemo-organotrophic. Could not reduce nitrate. Cellular fatty acids (>5%) are C_18:0_, C_18:1_ ω7c 11-methyl and summed feature 8 (C_18:1_ *ω*7c/C_18:1_ *ω*6c). Type species contained phosphatidyl ethanolamine, phosphatidyl choline, phosphatidyl glycerol, phosphatidyl monomethyl ethanolamine, an unidentified amino lipid, and an unidentified phospholipid as polar lipids. The DNA G + C content is around 58%. The type species is *Algilabrenzia polysiphoniae*.

#### Description of *Algilabrenzia polysiphoniae* comb. nov.

*Algilabrenzia polysiphoniae* (po.ly.si.pho’ni.ae. N.L. gen. n. polysiphoniae, of the red alga *Polysiphoni*)

#### Basonym: Labrenzia polysiphoniae Romanenko et al. 2019.

Synonym: *Roseibium polysiphoniae* (Romanenko *et al*. 2019) Liu *et al*. 2021.

The description is the same as for Roseibium polysiphoniae (Romanenko et al. 2019) Liu et al. 2021.

The type strain rh46^T^ (=KCTC 97111^T^ = KMM 9699^T^) was isolated from red alga *Polysiphonia* sp. The 16S rRNA gene sequence and genome accession number are LC381921 and JACYXJ00000000, respectively.

#### Description of *Hongsoonwoonella albiluteola* comb. nov.

*Hongsoonwoonella albiluteolla* (al.bi.lu.te.o’la. L. masc. adj. *albus*, white; L. masc. adj. *luteolus*, yellowish; N.L. fem. adj. *albiluteola*, yellowish-white coloured, referring to the colour of colonies of the type strain)

#### Basonym: *Stappia albiluteola* Jiang *et al.* 2021.

The description is the same as for *Stappia albiluteola* Jiang *et al.* 2021.

The type strain F7233^T^ (=KCTC 72859^T^ = MCCC 1H00419^T^) was isolated from marine sediment. The 16S rRNA gene sequence and genome accession number are MT742667 and JACFXV00000000, respectively.

## Supporting information

S1 Fig Graphical abstract. (TIF)

S2 Fig Transmission electron micrograph of strain MaLMAid0302^T^ (A), and strain SPO723^T^ (B). Both are cultivated on MA medium at 30°C for 48 hrs.
(TIF)

S3 Fig Two-dimensional TLC after staining with molybdatophosphoric acid showing the total polar lipid profiles of strain MaLMAid0302^T^ (A), and strain SPO723^T^ (B). Both are cultivated on MA medium at 30°C for 48 hrs. PG, phosphatidylglycerol; DPG, diphosphatidylglycerol; PE; phosphatidylethanolamine; L, unidentified polar lipid. AL, unidentified amino lipid; PL, unidentified phospholipid.
(TIF)

S4 Fig Clustering tree based on distance matrices: (A) ANI-score-based clustering tree, (B) AAI-score-based clustering tree. (TIF)

S5 Fig 16S rRNA-based ML tree including *Stappiaceae* species without genomic sequences. Bootstrap analysis was performed with 1,000 iterations.
(TIF)

S1 Table Differential chemotaxonomic characteristics of strains MaLMAid0302^T^, SPO723^T^, and related type strains of *Stappiaceae* (A) Substrate utilization profiles determined using the API NE system, (B) Substrate utilization profiles determined using the API 20E system, (C) Enzymatic activity profiles determined using the API ZYM system. (DOCX)

S2 Table Cellular fatty acid compositions (%) of the strains MaLMAid0302^T^, SPO723^T^, and the type strains of species of the genus *Pseudovibrio.* Strains: 1, the strain MaLMaid0302^T^; 2, the strain SPO723^T^; 3, *P. flavus* RKSG542^T^; 4, *P. stylochi* UST20140214-052^T^; 5, *P. hongkongensis* UST20140214-015B^T^; 6, *P. axinellae* Ad2^T^; 7, *P. japonicus* WSF2^T^; 8, *P. denitrificans* DN34^T^; 9, *P. ascidiaceicola* F423^T^; 10, *P. exalbescens* LA33B^T^. Fatty acids that represented <1% are not shown. -, not detected; tr, trace amount (<1.0%).
(DOCX)

S3 Table Isolation information and genome statistics of the studied bacterial strains. (DOCX)

S4 Table ANI values between genomes. (DOCX)

S5 Table dDDH values between genomes. (DOCX)

S6 Table AAI values between genomes. (DOCX)

S7 Table POCP values between genomes. (DOCX)

S8 Table Metabolic pathways associated with the major CCA axis, correlated with habitat types of strains. (DOCX)

S9 Table AAI values between newly reported *Stappiaceae* species, which were not included in this study, and closely related genus members. (DOCX)

## Abbreviations

ANI: average nucleotide identity
AAI: average amino acid identity
CFA: cellular fatty acids
