# Supplementary figures and images for: Genome-based reclassification of the family Stappiaceae and assessment of environmental forcing with the report of two novel taxa, Flexibacterium corallicola gen. nov., sp. nov., and Nesiotobacter zosterae sp. nov., isolated from coral and seagrass
Source: PLoS One. 2025 May 15;20(5):e0322500. doi: 10.1371/journal.pone.0322500 (PMC12080928; doi:10.1371/journal.pone.0322500)

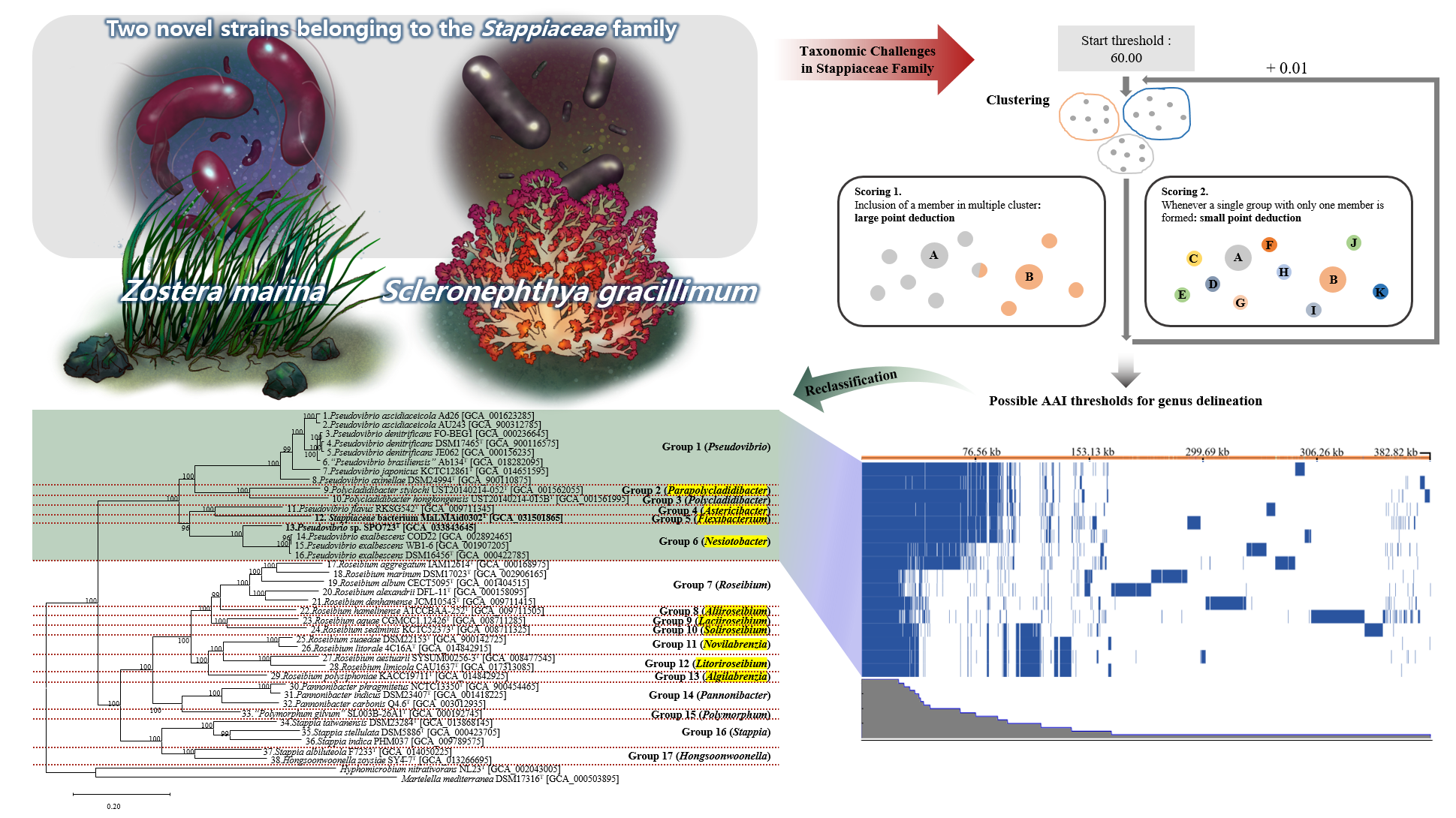

Supplement: S1 Fig — (TIF) [file pone.0322500.s001.tif]

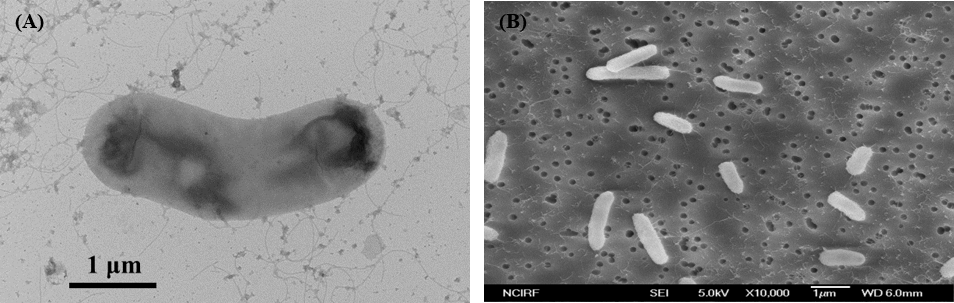

Supplement: S2 Fig — Both are cultivated on MA medium at 30°C for 48 hrs. (TIF) [file pone.0322500.s002.tif]

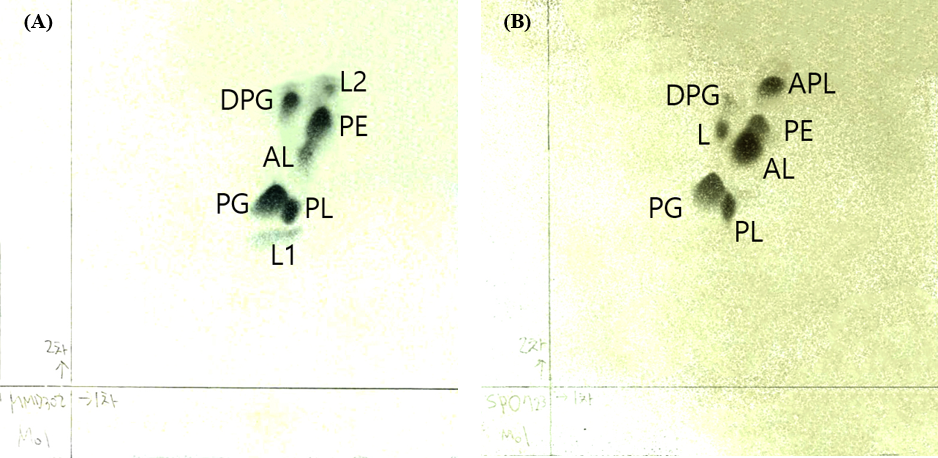

Supplement: S3 Fig — Both are cultivated on MA medium at 30°C for 48 hrs. PG, phosphatidylglycerol; DPG, diphosphatidylglycerol; PE; phosphatidylethanolamine; L, unidentified polar lipid. AL, unidentified amino lipid; PL, unidentified phospholipid. (TIF) [file pone.0322500.s003.tif]

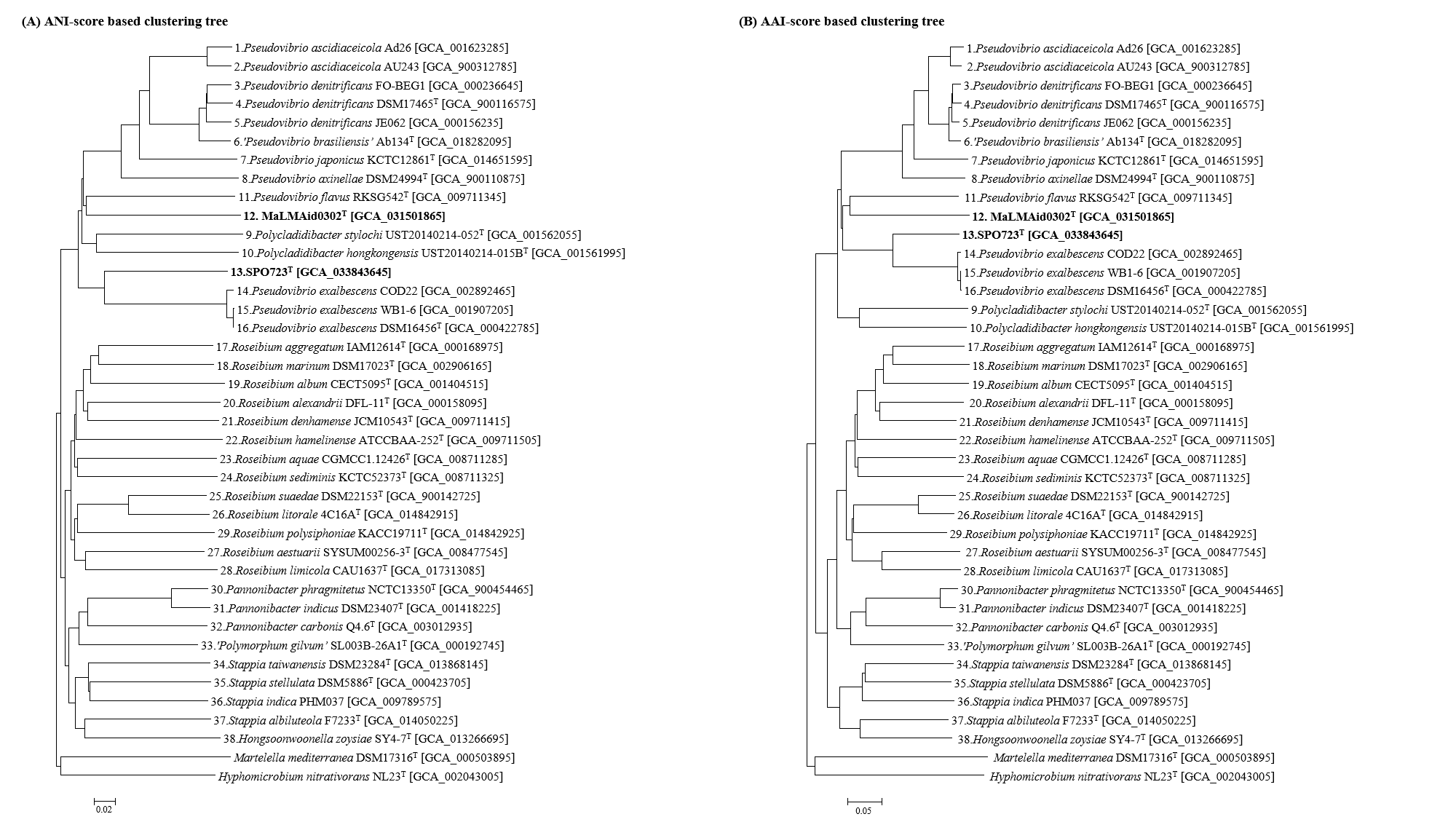

Supplement: S4 Fig — (TIF) [file pone.0322500.s004.tif]

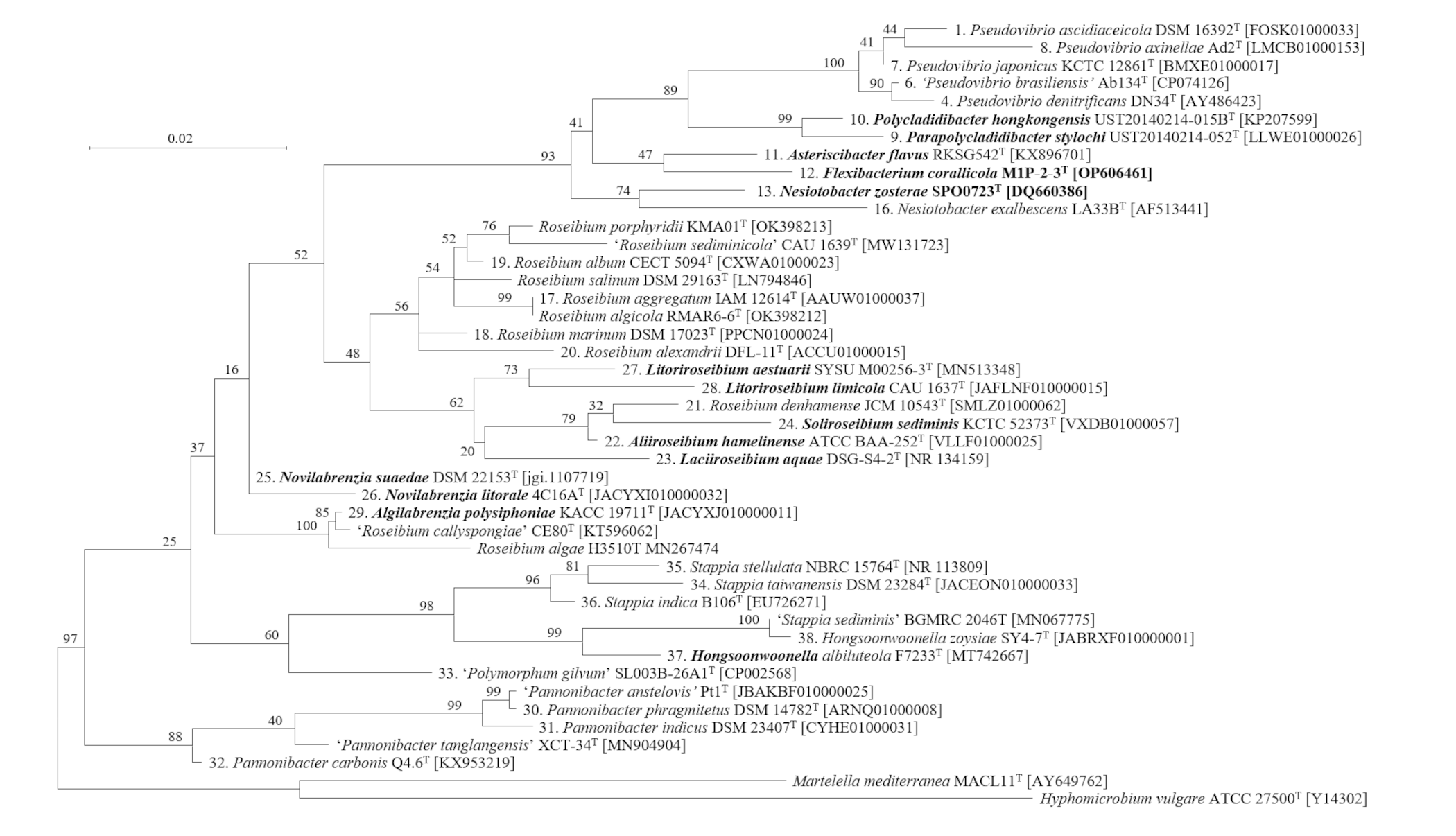

Supplement: S5 Fig — Bootstrap analysis was performed with 1,000 iterations. (TIF) [file pone.0322500.s005.tif]
