# Supplementary material for: Genome-based reclassification of the family Stappiaceae and assessment of environmental forcing with the report of two novel taxa, Flexibacterium corallicola gen. nov., sp. nov., and Nesiotobacter zosterae sp. nov., isolated from coral and seagrass
Source: PLoS One. 2025 May 15;20(5):e0322500. doi: 10.1371/journal.pone.0322500 (PMC12080928; doi:10.1371/journal.pone.0322500)
Supplement: S1 Table — (DOCX) [file pone.0322500.s006.docx]

**S1 Table. Differential chemotaxonomic characteristics of strain MaLMAid0302^T^, SPO723^T^, and related type strains of *Stappiaceae***

1. Substrate utilization tested using the API NE system

| API NE | | NO3 | TRP | GLU | ADH | URE | ESC | GEL | b-gal | GLU | ARA | MNE | MAN | NAG | MAL | GNT | CAP | ADI | MLT | CIT | PAC |
| --- | --- | --- | --- | --- | --- | --- | --- | --- | --- | --- | --- | --- | --- | --- | --- | --- | --- | --- | --- | --- | --- |
| *P. ascidiaceicola* | KCTC 12308^T^ | - | + | + | - | - | + | - | + | - | - | - | - | - | - | - | - | - | - | - | - |
| *P. denitrificans* | KCTC 62704^T^ | - | + | - | - | - | + | - | - | - | - | - | - | - | - | - | - | - | - | - | - |
| *P. japonicus* | KCTC 12861^T^ | - | + | - | - | - | + | - | + | - | - | - | - | - | - | - | - | - | - | - | - |
| *P. axinellae* | DSM 24994^T^ | + | + | - | - | - | + | - | - | - | - | - | - | - | - | - | - | - | - | - | - |
| *P. stylochi* | KCTC 42384^T^ | + | + | - | - | - | + | - | - | - | - | - | - | - | - | - | - | - | - | - | - |
| *P. hongkongensis* | KCTC 42383^T^ | + | - | - | - | - | + | - | - | - | - | - | - | - | - | - | - | - | - | - | - |
| *P. flavus* | ATCC TSD-76^T^ | + | + | - | - | - | + | - | - | - | - | - | - | - | - | - | - | - | - | - | - |
|  | MaLMAid0302 ^T^ | + | - | - | - | - | + | - | - | - | - | - | - | - | - | - | - | - | - | - | - |
|  | SPO723^T^ | - | + | + | + | - | + | + | + | + | - | + | - | + | + | w | - | - | + | - | w |
| *P. exalbescens* | DSM 16456^T^ | - | + | + | - | - | + | - | - | - | - | - | - | - | - | - | - | - | - | - | - |
|  |  | **potassium nitrate** | **L-tryptophane (INDole production)** | **D-glucose** | **L-arginine** | **Urea** | **esculin ferric citrate** | **gelatine** | **4-nitrophenyl-ßD-galactopyranoside** | **D-glucose** | **L-arabinose** | **D-mannose** | **D-mannitol** | **N-acetyl-glucosamine** | **D-maltose** | **potassium gluconate** | **capric acid** | **adipic acid** | **malic acid** | **trisodium citrate** | **phenylacetic acid** |

| API 20E | | b-gal | ADH | LDC | ODC | CIT | H_2_S | URE | TDA | IND | VP | GEL | GLU | MAN | INO | SOR | RHA | SAC | MEL | AMY | ARA | NO_2_ | N_2_ |
| --- | --- | --- | --- | --- | --- | --- | --- | --- | --- | --- | --- | --- | --- | --- | --- | --- | --- | --- | --- | --- | --- | --- | --- |
| *P. ascidiaceicola* | KCTC 12308^T^ | + | - | - | - | - | - | - | - | + | - | - | + | - | - | - | - | - | - | - | - | - | - |
| *P. denitrificans* | KCTC 62704^T^ | + | - | - | - | - | - | - | - | + | - | - | - | - | - | - | - | + | + | - | - | - | - |
| *P. japonicus* | KCTC 12861^T^ | - | - | - | - | - | - | - | - | + | - | - | + | - | - | - | - | - | - | - | - | - | - |
| *P. axinellae* | DSM 24994^T^ | - | - | - | - | - | - | - | - | + | - | - | - | - | - | - | - | - | - | - | - | - | - |
| *P. stylochi* | KCTC 42384^T^ | - | - | - | - | - | - | - | - | + | - | - | - | - | - | - | - | - | - | - | - | - | - |
| *P. hongkongensis* | KCTC 42383^T^ | - | - | - | - | - | - | - | - | - | - | - | - | - | - | - | - | - | - | - | - | - | - |
| *P. flavus* | ATCC TSD-76^T^ | - | - | - | - | - | - | - | - | + | - | - | - | - | - | - | - | - | - | - | - | + | - |
|  | MaLMAid0302 ^T^ | - | - | - | - | - | - | - | - | - | - | - | - | - | - | - | - | - | - | - | - | - | - |
|  | SPO723^T^ | + | + | - | - | - | - | - | - | + | + | + | + | - | - | - | - | - | - | - | - | - | - |
| *P. exalbescens* | DSM 16456^T^ | - | - | - | - | - | - | - | - | + | - | - | + | - | - | - | - | + | - | - | - | - | - |
|  |  | **2-nitrophenyl-ßDgalactopyranoside** | **L-arginine** | **L-lysine** | **L-ornithine** | **trisodium citrate** | **sodium thiosulfate** | **urea** | **L-tryptophane (Tryptophane DeAminase)** | **L-tryptophane (INDole production)** | **sodium pyruvate** | **Gelatin** | **D-glucose** | **D-mannitol** | **inositol** | **D-sorbitol** | **L-rhamnose** | **D-sucrose** | **D-melibiose** | **amygdalin** | **L-arabinose** | **potassium nitrate** | **potassium nitrate** |

1. Substrate utilization tested using the API 20E system
2. Enzymatic activity tested using the API ZYM system

| API ZYM | | C | AP | Es | EsLi | Li | Leu Ar | Val ar | Cys Ar | Tryp | a-chy | acP | AS-BI | a-gal | b-gal | glucuro | a-glc. | b-glc. | NAG | a-mann | a-fuc |
| --- | --- | --- | --- | --- | --- | --- | --- | --- | --- | --- | --- | --- | --- | --- | --- | --- | --- | --- | --- | --- | --- |
| *P. ascidiaceicola* | KCTC 12308^T^ | - | + | + | + | + | + | - | - | + | + | + | + | - | + | - | + | - | - | - | - |
| *P. denitrificans* | KCTC 62704^T^ | - | + | + | + | - | + | - | - | + | - | - | + | - | - | - | + | - | + | - | - |
| *P. japonicus* | KCTC 12861^T^ | - | + | + | + | - | + | + | - | + | + | + | + | - | - | - | - | + | - | - | - |
| *P. axinellae* | DSM 24994^T^ | - | + | + | + | - | + | + | - | + | - | + | + | - | - | - | - | - | - | - | - |
| *P. stylochi* | KCTC 42384^T^ | - | + | + | + | - | + | - | - | + | - | - | + | - | - | - | + | - | - | - | - |
| *P. hongkongensis* | KCTC 42383^T^ | - | + | + | + | - | + | - | - | + | - | + | + | - | - | - | + | - | + | - | - |
| *P. flavus* | ATCC TSD-76^T^ | - | + | + | - |  | + | - | - | - | - | - | + | - | - | - | + | - | + | - | - |
|  | MaLMAid0302 ^T^ | - | + | + | + | - | + | - | - | + | - | + | + | - | - | - | + | - | - | - | - |
|  | SPO723^T^ | - | + | + | + | + | + | + | w | + | + | + | + | - | + | - | + | - | + | - | - |
| *P. exalbescens* | DSM 16456^T^ | - | + | + | - | - | + | - | - | + | - | - | + | - | - | - | + | - | + | - | - |
|  |  | **Control** | **Alkaline phosphatase** | **Esterase (C4)** | **Esterase Lipase (C8)** | **Lipase (C14)** | **Leucine arylamidase** | **Valine arylamidase** | **Crystine arylamidase** | **Trypsin** | **α-chymotrypsin** | **Acid phospatase** | **Naphtol-AS-BI-phosphohydrolase** | **α-galactosidase** | **β-galactosidase** | **β-glucuronidase** | **α-glucosidase** | **β-glucosidase** | **N-Acetyl-β-glucosaminidase** | **α-Mannosidase** | **α-Fucosidase** |
