# Supplementary material for: Genome-based reclassification of the family Stappiaceae and assessment of environmental forcing with the report of two novel taxa, Flexibacterium corallicola gen. nov., sp. nov., and Nesiotobacter zosterae sp. nov., isolated from coral and seagrass
Source: PLoS One. 2025 May 15;20(5):e0322500. doi: 10.1371/journal.pone.0322500 (PMC12080928; doi:10.1371/journal.pone.0322500)
Supplement: S2 Table — Strains: 1, the strain MaLMaid0302T; 2, the strain SPO723T; 3, P. flavus RKSG542T; 4, P. stylochi UST20140214-052T; 5, P. hongkongensis UST20140214-015BT; 6, P. axinellae Ad2T; 7, P. japonicus WSF2T; 8, P. denitrificans DN34T; 9, P. ascidiaceicola F423T; 10, P. exalbescens LA33BT. Fatty acids that represented <1% are not shown. -, not detected; tr, trace amount (<1.0%). (DOCX) [file pone.0322500.s007.docx]

**S2 Table. Cellular fatty acid compositions (%) of the strains MaLMAid0302^T^, SPO723^T^, and the type strains of species of the genus *Pseudovibrio*.**

| **Fatty acid** | **1** | **2** | **3** | **4** | **5** | **6** | **7** | **8** | **9** | **10** |
| --- | --- | --- | --- | --- | --- | --- | --- | --- | --- | --- |
| C_14:0_ | - | - | 2.2 | tr | tr | tr | tr | - | - | - |
| C_16:0_ | 4.5 | 3.2 | 2.9 | 16.9 | 10.3 | 15.2 | 4.1 | 5.6 | 2.0 | 2.0 |
| C_17:0_ | tr | 3.6 | 2.8 | - | 1.8 | 1.9 | tr | 3.3 | - | tr |
| C_18:0_ | 4.5 | 7.3 | 6.5 | - | tr | 1.7 | 1.1 | 2.5 | 5.4 | 3.0 |
| C_20:1_ *ω*7*c* | tr | tr | tr | - | - | - | 1.6 | - | tr | tr |
| C_16:0_ 3-OH | - | - | - | 2.5 | 1.4 | 1.1 | 1.5 | tr | tr | - |
| C_18:0_ 3-OH | 2.4 | 3 | 2.1 | - | tr | 1.2 | tr | 1.7 | 4.0 | 3.0 |
| cyclo C_17:0_ | - | - | - | - | - | 2.5 | - | - | 0.2 | - |
| cyclo C_19:0_*ω*8*c* | 6.9 | 3.1 | 11.8 | 3.3 | 3.6 | 16.4 | 18.5 | 12.0 | 29.2 | 7.8 |
| 11-methyl C_18:1_*ω*7*c* | - | 2.4 | 1.9 | - | 4.3 | tr | tr | tr | 1.2 | 3.2 |
| Summed feature 2^*^ | 2.6 | 3 | 1.9 | tr | 1.7 | 2.2 | 2.5 | 2.0 | 2.7 | 2.6 |
| Summed feature 3^*^ | tr | tr | 1.7 | 9.3 | 5.6 | 21.7 | 2.3 | 1.7 | 2.8 | tr |
| Summed feature 7^*^ | - | tr | 1.3 | - | - | - | - | - | - | tr |
| Summed feature 8^*^ | 76.9 | 69.7 | 62.2 | 65.6 | 67.3 | 32.3 | 65.8 | 67.8 | 51.0 | 75.9 |

Strains: 1, MaLMaid0302^T^; 2, SPO723^T^; 3, *P. flavus* RKSG542^T^; 4, *P. stylochi* UST20140214-052^T^; 5, *P. hongkongensis* UST20140214-015B^T^; 6, *P. axinellae* Ad2^T^; 7, *P. japonicus* WSF2^T^; 8, *P. denitrificans* DN34^T^; 9, *P. ascidiaceicola* F423^T^; 10, *P. exalbescens* LA33B^T^. Fatty acids that represented <1 % are not shown. -, not detected; tr, trace amount(<1.0%).

*Summed feature 2 comprises the fatty acids C14:0 3-OH and/or iso-C16:1.

*Summed feature 3 comprises the fatty acids C16:1ω6c and/or C16:1ω7c.

*Summed feature 7 comprises the fatty acids unknown ECL 18.846 and/or C19:1ω6c.

*Summed feature 8 comprises the fatty acids C18:1ω6c and/or C18:1ω7c.
