# Supplementary material for: Genome-based reclassification of the family Stappiaceae and assessment of environmental forcing with the report of two novel taxa, Flexibacterium corallicola gen. nov., sp. nov., and Nesiotobacter zosterae sp. nov., isolated from coral and seagrass
Source: PLoS One. 2025 May 15;20(5):e0322500. doi: 10.1371/journal.pone.0322500 (PMC12080928; doi:10.1371/journal.pone.0322500)
Supplement: S3 Table — (DOCX) [file pone.0322500.s008.docx]

**S3 Table. Isolation information and genome statistics of the studied bacterial strains**

| No. | Genus | Species | Strain | Accession number | CheckM score (%) | | No. contigs | N50 | CDS | rRNA | tRNA | tm  RNA | misc  RNA | CRISPR repeat region | G+C content (%) | Size (Mb) |
| --- | --- | --- | --- | --- | --- | --- | --- | --- | --- | --- | --- | --- | --- | --- | --- | --- |
|  |  | /Subsp. |  |  | Completeness | Contamination |  | (kb) |  |  |  |  |  |  |  |  |
| 1 | *Pseudovibrio* | *ascidiaceicola* | Ad26 | GCA_001623285 | 99.58 | 0.82 | 159 | 293.31 | 5,769 | 5 | 61 | 1 | 44 | 0 | 51.2 | 6.18 |
| 2 | *Pseudovibrio* | *ascidiaceicola* | AU243 | GCA_900312785 | 100.00 | 0.32 | 23 | 512.36 | 5,330 | 9 | 62 | 1 | 37 | 0 | 51.3 | 5.75 |
| 3 | *Pseudovibrio* | *denitrificans* | FO-BEG1 | GCA_000236645 | 100.00 | 0.11 | 2 | 5,475.50 | 5,402 | 18 | 71 | 1 | 37 | 0 | 52.4 | 5.92 |
| 4 | *Pseudovibrio* | *denitrificans* | DSM17465^T^ | GCA_900116575 | 99.84 | 0.00 | 36 | 489.59 | 5,523 | 3 | 64 | 1 | 37 | 0 | 52.2 | 6.08 |
| 5 | *Pseudovibrio* | *denitrificans* | JE062 | GCA_000156235 | 99.21 | 0.00 | 19 | 509.30 | 5,192 | 35 | 86 | 1 | 34 | 0 | 52.5 | 5.73 |
| 6 | *Pseudovibrio* | *“brasiliensis”* | Ab134^T^ | GCA_018282095 | 100.00 | 0.00 | 6 | 4,836.77 | 5,394 | 18 | 75 | 1 | 37 | 0 | 52.1 | 6.03 |
| 7 | *Pseudovibrio* | *japonicus* | KCTC12861^T^ | GCA_014651595 | 100.00 | 0.35 | 25 | 642.97 | 4,551 | 4 | 63 | 1 | 26 | 0 | 52.7 | 4.96 |
| 8 | *Pseudovibrio* | *axinellae* | DSM24994^T^ | GCA_900110875 | 99.84 | 1.27 | 93 | 349.41 | 4,715 | 5 | 65 | 1 | 27 | 0 | 50.3 | 5.13 |
| 9 | *Polycladidibacter* | *stylochi* | UST20140214-052^T^ | GCA_001562055 | 97.45 | 0.32 | 43 | 294.22 | 3,349 | 3 | 64 | 1 | 25 | 1 | 47.0 | 3.68 |
| 10 | *Polycladidibacter* | *hongkongensis* | UST20140214-015B^T^ | GCA_001561995 | 98.10 | 0.00 | 39 | 340.15 | 3,534 | 4 | 62 | 1 | 24 | 0 | 53.3 | 3.75 |
| 11 | *Pseudovibrio* | *flavus* | RKSG542^T^ | GCA_009711345 | 99.37 | 0.63 | 66 | 264.09 | 3,815 | 3 | 54 | 1 | 24 | 5 | 52.6 | 4.29 |
| 12 |  |  | MaLMAid0302^T^ | GCA_031501865 | 98.28 | 0.95 | 8 | 4,644.43 | 4,550 | 21 | 72 | 1 | 25 | 0 | 49.4 | 4.96 |
| 13 |  |  | SPO723^T^ | GCA_033843645 | 99.58 | 0.19 | 29 | 389.82 | 3,867 | 8 | 60 | 1 | 25 | 1 | 55.5 | 4.29 |
| 14 | *Pseudovibrio* | *exalbescens* | COD22 | GCA_002892465 | 99.37 | 0.42 | 90 | 1,043.37 | 3,973 | 7 | 62 | 1 | 21 | 1 | 55.1 | 4.29 |
| 15 | *Pseudovibrio* | *exalbescens* | WB1-6 | GCA_001907205 | 99.05 | 0.03 | 52 | 389.80 | 3,810 | 7 | 62 | 1 | 21 | 0 | 55.1 | 4.17 |
| 16 | *Pseudovibrio* | *exalbescens* | DSM16456^T^ | GCA_000422785 | 99.05 | 0.03 | 36 | 389.44 | 3,815 | 3 | 55 | 1 | 21 | 0 | 55.1 | 4.16 |
| 17 | *Roseibium* | *aggregatum* | IAM12614^T^ | GCA_000168975 | 99.26 | 0.70 | 48 | 233.56 | 6,163 | 12 | 63 | 1 | 27 | 0 | 59.4 | 6.56 |
| 18 | *Roseibium* | *marinum* | DSM17023^T^ | GCA_002906165 | 99.68 | 1.64 | 29 | 366.71 | 5,500 | 3 | 51 | 1 | 26 | 0 | 60.3 | 6.06 |
| 19 | *Roseibium* | *album* | CECT5095^T^ | GCA_001404515 | 98.97 | 0.32 | 32 | 758.99 | 6,402 | 3 | 48 | 1 | 33 | 0 | 56.4 | 6.9 |
| 20 | *Roseibium* | *alexandrii* | DFL-11^T^ | GCA_000158095 | 99.29 | 1.32 | 3 | 5,300.19 | 5,152 | 9 | 52 | 1 | 27 | 0 | 56.4 | 5.46 |
| 21 | *Roseibium* | *denhamense* | JCM10543^T^ | GCA_009711415 | 99.68 | 1.11 | 116 | 200.37 | 4,489 | 3 | 44 | 1 | 26 | 0 | 57.3 | 4.81 |
| 22 | *Roseibium* | *hamelinense* | ATCCBAA-252^T^ | GCA_009711505 | 98.34 | 0.37 | 25 | 386.84 | 4,442 | 3 | 47 | 1 | 25 | 0 | 56.4 | 4.72 |
| 23 | *Roseibium* | *aquae* | CGMCC1.12426^T^ | GCA_008711285 | 99.29 | 0.21 | 37 | 344.32 | 4,134 | 5 | 45 | 2 | 24 | 1 | 61.0 | 4.41 |
| 24 | *Roseibium* | *sediminis* | KCTC52373^T^ | GCA_008711325 | 99.68 | 0.42 | 117 | 196.50 | 4,724 | 3 | 49 | 1 | 31 | 0 | 57.0 | 4.99 |
| 25 | *Roseibium* | *suaedae* | DSM22153^T^ | GCA_900142725 | 99.68 | 0.00 | 14 | 1,462.57 | 4,555 | 3 | 47 | 1 | 29 | 0 | 60.2 | 5.14 |
| 26 | *Roseibium* | *litorale* | 4C16A^T^ | GCA_014842915 | 99.37 | 0.00 | 35 | 272.20 | 4,560 | 3 | 47 | 1 | 28 | 0 | 59.8 | 4.94 |
| 27 | *Roseibium* | *aestuarii* | SYSUM00256-3^T^ | GCA_008477545 | 99.37 | 0.16 | 21 | 960.75 | 3,866 | 3 | 58 | 1 | 23 | 1 | 64.8 | 4.35 |
| 28 | *Roseibium* | *limicola* | CAU1637^T^ | GCA_017313085 | 99.26 | 0.16 | 17 | 581.05 | 3,993 | 3 | 51 | 1 | 26 | 2 | 58.4 | 4.51 |
| 29 | *Roseibium* | *polysiphoniae* | KACC19711^T^ | GCA_014842925 | 100.00 | 0.32 | 12 | 594.25 | 4,278 | 3 | 45 | 1 | 20 | 0 | 58.1 | 4.58 |
| 30 | *Pannonibacter* | *phragmitetus* | NCTC13350^T^ | GCA_900454465 | 98.31 | 0.16 | 2 | 4,621.34 | 4,301 | 9 | 55 | 1 | 39 | 4 | 63.1 | 4.84 |
| 31 | *Pannonibacter* | *indicus* | DSM23407^T^ | GCA_001418225 | 99.47 | 0.16 | 36 | 216.08 | 3,863 | 5 | 56 | 1 | 33 | 4 | 63.5 | 4.17 |
| 32 | *Pannonibacter* | *carbonis* | Q4.6^T^ | GCA_003012935 | 100.00 | 1.01 | 33 | 267.53 | 4,394 | 4 | 54 | 1 | 33 | 2 | 63.6 | 4.78 |
| 33 | *“Polymorphum”* | *“gilvum”* | SL003B-26A1^T^ | GCA_000192745 | 99.81 | 0.00 | 2 | 4,649.22 | 4,477 | 6 | 57 | 1 | 24 | 1 | 67.1 | 4.72 |
| 34 | *Stappia* | *taiwanensis* | DSM23284^T^ | GCA_013868145 | 99.89 | 0.16 | 38 | 175.15 | 3,944 | 4 | 48 | 1 | 25 | 4 | 65.4 | 4.43 |
| 35 | *Stappia* | *stellulata* | DSM5886^T^ | GCA_000423705 | 98.95 | 0.95 | 12 | 994.44 | 4,260 | 12 | 55 | 1 | 25 | 0 | 64.7 | 4.62 |
| 36 | *Stappia* | *indica* | PHM037 | GCA_009789575 | 99.89 | 0.47 | 1 | 5,116.76 | 4,588 | 6 | 53 | 1 | 23 | 2 | 67.3 | 5.12 |
| 37 | *Stappia* | *albiluteola* | F7233^T^ | GCA_014050225 | 99.68 | 0.32 | 70 | 209.37 | 4,081 | 9 | 52 | 1 | 23 | 1 | 63.3 | 4.37 |
| 38 | *Hongsoonwoonella* | *zoysiae* | SY4-7^T^ | GCA_013266695 | 99.37 | 0.32 | 6 | 2,434.45 | 4,002 | 6 | 50 | 1 | 22 | 0 | 60.8 | 4.36 |
