# Supplementary material for: Genome-based reclassification of the family Stappiaceae and assessment of environmental forcing with the report of two novel taxa, Flexibacterium corallicola gen. nov., sp. nov., and Nesiotobacter zosterae sp. nov., isolated from coral and seagrass
Source: PLoS One. 2025 May 15;20(5):e0322500. doi: 10.1371/journal.pone.0322500 (PMC12080928; doi:10.1371/journal.pone.0322500)
Supplement: S4 Table — (DOCX) [file pone.0322500.s009.docx]

**S4 Table. ANI values between genomes.**

| ANI | 1 | 2 | 3 | 4 | 5 | 6 | 7 | 8 | 9 | 10 | 11 | 12 | 13 | 14 | 15 | 16 | 17 | 18 | 19 | 20 | 21 | 22 | 23 | 24 | 25 | 26 | 27 | 28 | 29 | 30 | 31 | 32 | 33 | 34 | 35 | 36 | 37 | 38 |
| --- | --- | --- | --- | --- | --- | --- | --- | --- | --- | --- | --- | --- | --- | --- | --- | --- | --- | --- | --- | --- | --- | --- | --- | --- | --- | --- | --- | --- | --- | --- | --- | --- | --- | --- | --- | --- | --- | --- |
| 1 |  | 95.37 | 84.41 | 84.53 | 84.57 | 84.47 | 82.07 | 78.71 | 70.28 | 70.71 | 71.39 | **70.57** | **71.35** | 70.42 | 70.31 | 70.15 | 69.05 | 68.81 | 68.48 | 68.93 | 68.36 | 68.53 | 68.33 | 68.83 | 69.24 | 69.31 | 69.29 | 68.94 | 68.97 | 69.27 | 69.24 | 68.83 | 68.83 | 68.97 | 68.55 | 68.69 | 68.59 | 68.37 |
| 2 | 95.37 |  | 84.41 | 84.58 | 84.61 | 84.70 | 81.98 | 78.83 | 70.58 | 70.66 | 71.54 | **70.57** | **71.19** | 70.60 | 70.40 | 70.42 | 69.27 | 68.94 | 68.48 | 68.79 | 68.49 | 68.41 | 68.39 | 68.98 | 69.53 | 69.01 | 69.56 | 69.24 | 69.04 | 69.20 | 69.34 | 68.99 | 68.93 | 68.98 | 68.52 | 68.73 | 68.85 | 68.54 |
| 3 | 84.41 | 84.41 |  | 95.34 | 95.36 | 93.80 | 82.05 | 78.42 | 70.15 | 70.80 | 71.86 | **70.74** | **71.74** | 70.70 | 70.83 | 70.76 | 69.50 | 69.23 | 68.88 | 69.08 | 68.92 | 68.58 | 68.91 | 69.36 | 69.71 | 69.65 | 70.05 | 69.61 | 69.38 | 69.66 | 69.55 | 69.59 | 69.45 | 69.26 | 69.11 | 69.36 | 69.06 | 68.66 |
| 4 | 84.53 | 84.58 | 95.34 |  | 95.08 | 93.96 | 81.91 | 78.49 | 70.32 | 70.89 | 72.00 | **70.55** | **71.61** | 70.92 | 70.75 | 70.48 | 69.68 | 69.41 | 68.74 | 69.09 | 68.96 | 68.55 | 68.74 | 69.38 | 69.81 | 69.69 | 69.87 | 69.73 | 69.24 | 69.79 | 69.52 | 69.54 | 69.47 | 69.26 | 69.12 | 69.22 | 69.29 | 68.76 |
| 5 | 84.57 | 84.61 | 95.36 | 95.08 |  | 93.97 | 81.99 | 78.53 | 70.54 | 70.61 | 71.88 | **70.95** | **71.92** | 70.82 | 70.61 | 70.38 | 69.58 | 69.35 | 68.85 | 69.14 | 68.75 | 68.59 | 68.93 | 69.33 | 69.66 | 69.68 | 70.02 | 69.59 | 69.38 | 70.06 | 69.60 | 69.27 | 69.36 | 69.46 | 69.05 | 69.40 | 69.15 | 68.58 |
| 6 | 84.47 | 84.70 | 93.80 | 93.96 | 93.97 |  | 82.19 | 78.42 | 70.36 | 70.80 | 71.99 | **71.00** | **71.77** | 70.94 | 70.65 | 70.61 | 69.65 | 69.41 | 69.06 | 69.02 | 68.72 | 68.57 | 68.84 | 69.18 | 69.64 | 69.66 | 69.62 | 69.45 | 69.33 | 70.11 | 69.66 | 69.56 | 69.55 | 69.17 | 69.00 | 69.46 | 69.37 | 68.56 |
| 7 | 82.07 | 81.98 | 82.05 | 81.91 | 81.99 | 82.19 |  | 77.61 | 70.09 | 70.82 | 71.28 | **70.53** | **71.46** | 70.44 | 70.63 | 70.61 | 69.67 | 69.26 | 68.80 | 69.09 | 69.02 | 68.67 | 68.62 | 69.37 | 69.92 | 69.55 | 69.87 | 69.48 | 69.35 | 69.78 | 69.71 | 69.36 | 69.27 | 69.05 | 68.90 | 69.18 | 69.16 | 68.79 |
| 8 | 78.71 | 78.83 | 78.42 | 78.49 | 78.53 | 78.42 | 77.61 |  | 70.16 | 70.17 | 70.83 | **70.60** | **70.71** | 69.99 | 70.03 | 70.06 | 68.71 | 68.57 | 68.39 | 68.39 | 68.08 | 68.15 | 68.01 | 68.64 | 68.84 | 68.76 | 68.70 | 68.93 | 68.49 | 68.82 | 68.55 | 68.62 | 68.33 | 68.45 | 68.23 | 68.32 | 68.26 | 67.77 |
| 9 | 70.28 | 70.58 | 70.15 | 70.32 | 70.54 | 70.36 | 70.09 | 70.16 |  | 72.73 | 70.02 | **69.79** | **70.24** | 69.59 | 69.51 | 69.51 | 68.77 | 68.25 | 68.15 | 68.25 | 67.91 | 68.12 | 68.03 | 68.63 | 68.50 | 68.36 | 68.98 | 68.63 | 68.35 | 68.67 | 68.75 | 68.22 | 68.50 | 68.65 | 68.16 | 68.27 | 68.47 | 67.94 |
| 10 | 70.71 | 70.66 | 70.80 | 70.89 | 70.61 | 70.80 | 70.82 | 70.17 | 72.73 |  | 70.31 | **70.04** | **70.66** | 70.20 | 70.10 | 70.21 | 69.23 | 68.94 | 68.33 | 68.93 | 68.71 | 68.50 | 68.73 | 69.05 | 69.28 | 69.25 | 69.31 | 69.12 | 68.85 | 69.33 | 69.46 | 69.34 | 69.45 | 69.47 | 68.99 | 69.18 | 69.05 | 68.64 |
| 11 | 71.39 | 71.54 | 71.86 | 72.00 | 71.88 | 71.99 | 71.28 | 70.83 | 70.02 | 70.31 |  | **71.31** | **71.45** | 70.57 | 70.46 | 70.47 | 69.56 | 69.06 | 68.96 | 69.16 | 68.66 | 68.34 | 68.70 | 69.49 | 69.61 | 69.72 | 69.77 | 69.41 | 69.10 | 69.63 | 69.59 | 69.43 | 69.09 | 69.22 | 68.81 | 69.21 | 69.26 | 69.09 |
| 12 | **70.57** | **70.57** | **70.74** | **70.55** | **70.95** | **71.00** | **70.53** | **70.60** | **69.79** | **70.04** | **71.31** |  | **70.48** | **69.95** | **70.13** | **70.03** | **69.29** | **68.79** | **68.24** | **68.59** | **68.50** | **68.16** | **68.46** | **68.62** | **69.47** | **69.10** | **69.52** | **68.80** | **68.74** | **69.08** | **68.65** | **68.71** | **68.91** | **68.55** | **68.72** | **68.47** | **68.71** | **68.00** |
| 13 | **71.35** | **71.19** | **71.74** | **71.61** | **71.92** | **71.77** | **71.46** | **70.71** | **70.24** | **70.66** | **71.45** | **70.48** |  | **76.12** | **76.05** | **76.24** | **70.16** | **69.91** | **69.07** | **69.68** | **69.14** | **69.46** | **69.55** | **69.95** | **70.27** | **70.01** | **70.48** | **69.78** | **69.62** | **70.19** | **70.60** | **70.03** | **70.33** | **70.19** | **69.65** | **70.01** | **69.57** | **69.45** |
| 14 | 70.42 | 70.60 | 70.70 | 70.92 | 70.82 | 70.94 | 70.44 | 69.99 | 69.59 | 70.20 | 70.57 | **69.95** | **76.12** |  | 98.77 | 98.77 | 69.85 | 69.64 | 69.16 | 69.06 | 69.06 | 68.86 | 69.35 | 69.44 | 69.66 | 69.69 | 69.80 | 69.53 | 69.13 | 69.94 | 69.85 | 69.66 | 69.95 | 69.57 | 69.45 | 69.67 | 69.07 | 69.28 |
| 15 | 70.31 | 70.40 | 70.83 | 70.75 | 70.61 | 70.65 | 70.63 | 70.03 | 69.51 | 70.10 | 70.46 | **70.13** | **76.05** | 98.77 |  | 99.98 | 69.58 | 69.52 | 68.79 | 69.00 | 69.21 | 68.73 | 69.04 | 69.45 | 69.69 | 69.74 | 69.86 | 69.68 | 68.93 | 69.83 | 69.87 | 69.43 | 69.63 | 69.55 | 69.70 | 69.87 | 69.31 | 68.94 |
| 16 | 70.15 | 70.42 | 70.76 | 70.48 | 70.38 | 70.61 | 70.61 | 70.06 | 69.51 | 70.21 | 70.47 | **70.03** | **76.24** | 98.77 | 99.98 |  | 69.48 | 69.37 | 68.80 | 68.95 | 69.15 | 68.37 | 68.85 | 69.14 | 69.58 | 69.61 | 69.83 | 69.36 | 69.02 | 69.87 | 69.58 | 68.95 | 69.76 | 69.43 | 69.23 | 69.60 | 69.14 | 68.92 |
| 17 | 69.05 | 69.27 | 69.50 | 69.68 | 69.58 | 69.65 | 69.67 | 68.71 | 68.77 | 69.23 | 69.56 | **69.29** | **70.16** | 69.85 | 69.58 | 69.48 |  | 78.22 | 75.54 | 74.97 | 74.85 | 72.98 | 73.54 | 73.81 | 74.52 | 74.40 | 74.49 | 73.50 | 73.59 | 73.49 | 73.30 | 73.36 | 74.07 | 72.64 | 72.16 | 72.80 | 72.21 | 71.66 |
| 18 | 68.81 | 68.94 | 69.23 | 69.41 | 69.35 | 69.41 | 69.26 | 68.57 | 68.25 | 68.94 | 69.06 | **68.79** | **69.91** | 69.64 | 69.52 | 69.37 | 78.22 |  | 75.70 | 74.55 | 74.62 | 72.62 | 73.37 | 73.29 | 74.04 | 74.04 | 74.34 | 73.11 | 73.44 | 73.14 | 73.48 | 73.52 | 74.30 | 72.63 | 72.24 | 72.96 | 72.48 | 71.97 |
| 19 | 68.48 | 68.48 | 68.88 | 68.74 | 68.85 | 69.06 | 68.80 | 68.39 | 68.15 | 68.33 | 68.96 | **68.24** | **69.07** | 69.16 | 68.79 | 68.80 | 75.54 | 75.70 |  | 73.75 | 73.70 | 71.73 | 72.49 | 72.31 | 72.49 | 72.45 | 72.66 | 71.83 | 72.35 | 71.65 | 71.77 | 71.51 | 72.36 | 70.96 | 70.66 | 71.06 | 70.68 | 70.54 |
| 20 | 68.93 | 68.79 | 69.08 | 69.09 | 69.14 | 69.02 | 69.09 | 68.39 | 68.25 | 68.93 | 69.16 | **68.59** | **69.68** | 69.06 | 69.00 | 68.95 | 74.97 | 74.55 | 73.75 |  | 75.11 | 72.46 | 72.43 | 72.48 | 72.66 | 72.37 | 72.63 | 72.16 | 72.76 | 71.69 | 71.68 | 71.45 | 72.06 | 70.82 | 70.73 | 70.80 | 70.58 | 70.51 |
| 21 | 68.36 | 68.49 | 68.92 | 68.96 | 68.75 | 68.72 | 69.02 | 68.08 | 67.91 | 68.71 | 68.66 | **68.50** | **69.14** | 69.06 | 69.21 | 69.15 | 74.85 | 74.62 | 73.70 | 75.11 |  | 73.05 | 72.56 | 72.61 | 72.71 | 72.51 | 72.62 | 72.08 | 72.36 | 71.79 | 71.74 | 71.65 | 72.24 | 71.11 | 70.63 | 71.22 | 70.58 | 70.34 |
| 22 | 68.53 | 68.41 | 68.58 | 68.55 | 68.59 | 68.57 | 68.67 | 68.15 | 68.12 | 68.50 | 68.34 | **68.16** | **69.46** | 68.86 | 68.73 | 68.37 | 72.98 | 72.62 | 71.73 | 72.46 | 73.05 |  | 72.88 | 72.32 | 72.38 | 72.27 | 72.49 | 71.70 | 71.99 | 71.59 | 71.47 | 71.29 | 72.20 | 70.83 | 70.81 | 70.93 | 70.72 | 70.29 |
| 23 | 68.33 | 68.39 | 68.91 | 68.74 | 68.93 | 68.84 | 68.62 | 68.01 | 68.03 | 68.73 | 68.70 | **68.46** | **69.55** | 69.35 | 69.04 | 68.85 | 73.54 | 73.37 | 72.49 | 72.43 | 72.56 | 72.88 |  | 73.59 | 72.94 | 72.90 | 72.85 | 71.99 | 72.59 | 72.46 | 72.32 | 72.38 | 73.25 | 71.58 | 71.19 | 71.87 | 71.66 | 70.79 |
| 24 | 68.83 | 68.98 | 69.36 | 69.38 | 69.33 | 69.18 | 69.37 | 68.64 | 68.63 | 69.05 | 69.49 | **68.62** | **69.95** | 69.44 | 69.45 | 69.14 | 73.81 | 73.29 | 72.31 | 72.48 | 72.61 | 72.32 | 73.59 |  | 73.44 | 73.39 | 73.35 | 72.48 | 73.08 | 72.85 | 72.55 | 72.25 | 72.63 | 71.27 | 71.28 | 71.49 | 71.21 | 70.85 |
| 25 | 69.24 | 69.53 | 69.71 | 69.81 | 69.66 | 69.64 | 69.92 | 68.84 | 68.50 | 69.28 | 69.61 | **69.47** | **70.27** | 69.66 | 69.69 | 69.58 | 74.52 | 74.04 | 72.49 | 72.66 | 72.71 | 72.38 | 72.94 | 73.44 |  | 85.01 | 75.55 | 73.86 | 74.74 | 74.21 | 74.11 | 74.06 | 74.43 | 72.41 | 72.11 | 72.63 | 72.05 | 71.46 |
| 26 | 69.31 | 69.01 | 69.65 | 69.69 | 69.68 | 69.66 | 69.55 | 68.76 | 68.36 | 69.25 | 69.72 | **69.10** | **70.01** | 69.69 | 69.74 | 69.61 | 74.40 | 74.04 | 72.45 | 72.37 | 72.51 | 72.27 | 72.90 | 73.39 | 85.01 |  | 75.17 | 73.42 | 74.40 | 73.92 | 74.22 | 73.67 | 74.15 | 72.53 | 72.06 | 72.69 | 72.05 | 71.48 |
| 27 | 69.29 | 69.56 | 70.05 | 69.87 | 70.02 | 69.62 | 69.87 | 68.70 | 68.98 | 69.31 | 69.77 | **69.52** | **70.48** | 69.80 | 69.86 | 69.83 | 74.49 | 74.34 | 72.66 | 72.63 | 72.62 | 72.49 | 72.85 | 73.35 | 75.55 | 75.17 |  | 76.17 | 74.19 | 74.55 | 74.38 | 74.33 | 75.26 | 73.54 | 72.80 | 73.79 | 73.13 | 72.14 |
| 28 | 68.94 | 69.24 | 69.61 | 69.73 | 69.59 | 69.45 | 69.48 | 68.93 | 68.63 | 69.12 | 69.41 | **68.80** | **69.78** | 69.53 | 69.68 | 69.36 | 73.50 | 73.11 | 71.83 | 72.16 | 72.08 | 71.70 | 71.99 | 72.48 | 73.86 | 73.42 | 76.17 |  | 73.26 | 72.38 | 72.35 | 72.31 | 73.01 | 71.80 | 71.34 | 71.81 | 71.24 | 70.71 |
| 29 | 68.97 | 69.04 | 69.38 | 69.24 | 69.38 | 69.33 | 69.35 | 68.49 | 68.35 | 68.85 | 69.10 | **68.74** | **69.62** | 69.13 | 68.93 | 69.02 | 73.59 | 73.44 | 72.35 | 72.76 | 72.36 | 71.99 | 72.59 | 73.08 | 74.74 | 74.40 | 74.19 | 73.26 |  | 72.78 | 72.61 | 72.60 | 73.36 | 71.58 | 71.73 | 71.67 | 71.34 | 70.83 |
| 30 | 69.27 | 69.20 | 69.66 | 69.79 | 70.06 | 70.11 | 69.78 | 68.82 | 68.67 | 69.33 | 69.63 | **69.08** | **70.19** | 69.94 | 69.83 | 69.87 | 73.49 | 73.14 | 71.65 | 71.69 | 71.79 | 71.59 | 72.46 | 72.85 | 74.21 | 73.92 | 74.55 | 72.38 | 72.78 |  | 92.92 | 77.38 | 76.04 | 73.42 | 72.98 | 73.64 | 73.17 | 72.31 |
| 31 | 69.24 | 69.34 | 69.55 | 69.52 | 69.60 | 69.66 | 69.71 | 68.55 | 68.75 | 69.46 | 69.59 | **68.65** | **70.60** | 69.85 | 69.87 | 69.58 | 73.30 | 73.48 | 71.77 | 71.68 | 71.74 | 71.47 | 72.32 | 72.55 | 74.11 | 74.22 | 74.38 | 72.35 | 72.61 | 92.92 |  | 76.84 | 76.43 | 73.30 | 72.74 | 73.82 | 73.01 | 72.14 |
| 32 | 68.83 | 68.99 | 69.59 | 69.54 | 69.27 | 69.56 | 69.36 | 68.62 | 68.22 | 69.34 | 69.43 | **68.71** | **70.03** | 69.66 | 69.43 | 68.95 | 73.36 | 73.52 | 71.51 | 71.45 | 71.65 | 71.29 | 72.38 | 72.25 | 74.06 | 73.67 | 74.33 | 72.31 | 72.60 | 77.38 | 76.84 |  | 76.62 | 73.23 | 72.99 | 73.97 | 73.07 | 72.09 |
| 33 | 68.83 | 68.93 | 69.45 | 69.47 | 69.36 | 69.55 | 69.27 | 68.33 | 68.50 | 69.45 | 69.09 | **68.91** | **70.33** | 69.95 | 69.63 | 69.76 | 74.07 | 74.30 | 72.36 | 72.06 | 72.24 | 72.20 | 73.25 | 72.63 | 74.43 | 74.15 | 75.26 | 73.01 | 73.36 | 76.04 | 76.43 | 76.62 |  | 74.65 | 74.32 | 75.45 | 74.81 | 73.32 |
| 34 | 68.97 | 68.98 | 69.26 | 69.26 | 69.46 | 69.17 | 69.05 | 68.45 | 68.65 | 69.47 | 69.22 | **68.55** | **70.19** | 69.57 | 69.55 | 69.43 | 72.64 | 72.63 | 70.96 | 70.82 | 71.11 | 70.83 | 71.58 | 71.27 | 72.41 | 72.53 | 73.54 | 71.80 | 71.58 | 73.42 | 73.30 | 73.23 | 74.65 |  | 76.91 | 77.01 | 74.34 | 73.43 |
| 35 | 68.55 | 68.52 | 69.11 | 69.12 | 69.05 | 69.00 | 68.90 | 68.23 | 68.16 | 68.99 | 68.81 | **68.72** | **69.65** | 69.45 | 69.70 | 69.23 | 72.16 | 72.24 | 70.66 | 70.73 | 70.63 | 70.81 | 71.19 | 71.28 | 72.11 | 72.06 | 72.80 | 71.34 | 71.73 | 72.98 | 72.74 | 72.99 | 74.32 | 76.91 |  | 77.31 | 74.27 | 73.33 |
| 36 | 68.69 | 68.73 | 69.36 | 69.22 | 69.40 | 69.46 | 69.18 | 68.32 | 68.27 | 69.18 | 69.21 | **68.47** | **70.01** | 69.67 | 69.87 | 69.60 | 72.80 | 72.96 | 71.06 | 70.80 | 71.22 | 70.93 | 71.87 | 71.49 | 72.63 | 72.69 | 73.79 | 71.81 | 71.67 | 73.64 | 73.82 | 73.97 | 75.45 | 77.01 | 77.31 |  | 74.80 | 73.51 |
| 37 | 68.59 | 68.85 | 69.06 | 69.29 | 69.15 | 69.37 | 69.16 | 68.26 | 68.47 | 69.05 | 69.26 | **68.71** | **69.57** | 69.07 | 69.31 | 69.14 | 72.21 | 72.48 | 70.68 | 70.58 | 70.58 | 70.72 | 71.66 | 71.21 | 72.05 | 72.05 | 73.13 | 71.24 | 71.34 | 73.17 | 73.01 | 73.07 | 74.81 | 74.34 | 74.27 | 74.80 |  | 75.22 |
| 38 | 68.37 | 68.54 | 68.66 | 68.76 | 68.58 | 68.56 | 68.79 | 67.77 | 67.94 | 68.64 | 69.09 | **68.00** | **69.45** | 69.28 | 68.94 | 68.92 | 71.66 | 71.97 | 70.54 | 70.51 | 70.34 | 70.29 | 70.79 | 70.85 | 71.46 | 71.48 | 72.14 | 70.71 | 70.83 | 72.31 | 72.14 | 72.09 | 73.32 | 73.43 | 73.33 | 73.51 | 75.22 |  |
