# Supplementary material for: Genome-based reclassification of the family Stappiaceae and assessment of environmental forcing with the report of two novel taxa, Flexibacterium corallicola gen. nov., sp. nov., and Nesiotobacter zosterae sp. nov., isolated from coral and seagrass
Source: PLoS One. 2025 May 15;20(5):e0322500. doi: 10.1371/journal.pone.0322500 (PMC12080928; doi:10.1371/journal.pone.0322500)
Supplement: S5 Table — (DOCX) [file pone.0322500.s010.docx]

**S5 Table. *d*DDH values between genomes.**

| dDDH | 1 | 2 | 3 | 4 | 5 | 6 | 7 | 8 | 9 | 10 | 11 | 12 | 13 | 14 | 15 | 16 | 17 | 18 | 19 | 20 | 21 | 22 | 23 | 24 | 25 | 26 | 27 | 28 | 29 | 30 | 31 | 32 | 33 | 34 | 35 | 36 | 37 | 38 |
| --- | --- | --- | --- | --- | --- | --- | --- | --- | --- | --- | --- | --- | --- | --- | --- | --- | --- | --- | --- | --- | --- | --- | --- | --- | --- | --- | --- | --- | --- | --- | --- | --- | --- | --- | --- | --- | --- | --- |
| 1 |  | 61.8 | 27.3 | 27.4 | 27.4 | 27.6 | 24.5 | 22.1 | 20.5 | 20.9 | 20.1 | **22.2** | **19.8** | 19.1 | 19.6 | 19.2 | 20.9 | 19.6 | 20.5 | 21.0 | 19.4 | 19.9 | 26.1 | 20.2 | 19.4 | 19.6 | 19.0 | 19.0 | 18.8 | 20.0 | 20.2 | 19.8 | 20.3 | 19.3 | 20.5 | 18.3 | 19.8 | 19.8 |
| 2 | 61.8 |  | 27.4 | 27.4 | 27.6 | 27.7 | 24.5 | 21.9 | 21.2 | 21.4 | 20.1 | **23.0** | **20.4** | 19.3 | 19.2 | 18.9 | 21.0 | 19.4 | 20.7 | 21.1 | 20.7 | 20.1 | 24.5 | 20.6 | 19.3 | 19.8 | 18.9 | 20.0 | 18.9 | 21.3 | 21.1 | 19.2 | 22.7 | 19.8 | 20.1 | 19.4 | 20.8 | 19.7 |
| 3 | 27.3 | 27.4 |  | 61.9 | 61.8 | 54.0 | 24.8 | 21.4 | 22.0 | 22.5 | 20.5 | **24.0** | **20.8** | 20.2 | 20.1 | 19.4 | 21.7 | 21.6 | 23.7 | 22.8 | 22.5 | 20.9 | 24.2 | 21.2 | 21.4 | 20.6 | 20.0 | 21.5 | 21.0 | 20.7 | 19.4 | 21.5 | 21.8 | 19.8 | 21.4 | 21.5 | 22.3 | 20.7 |
| 4 | 27.4 | 27.4 | 61.9 |  | 60.8 | 54.4 | 24.7 | 21.3 | 20.9 | 21.3 | 20.1 | **22.6** | **20.4** | 19.5 | 19.5 | 19.2 | 20.3 | 19.7 | 20.2 | 20.3 | 19.6 | 19.6 | 22.6 | 19.6 | 19.2 | 19.1 | 18.8 | 19.6 | 19.0 | 19.3 | 16.6 | 19.7 | 20.0 | 18.5 | 19.8 | 19.2 | 20.3 | 18.9 |
| 5 | 27.4 | 27.6 | 61.8 | 60.8 |  | 54.5 | 24.9 | 21.5 | 23.0 | 23.7 | 21.4 | **24.6** | **21.4** | 21.4 | 21.1 | 19.9 | 23.7 | 22.9 | 25.6 | 24.0 | 24.9 | 24.8 | 36.2 | 23.7 | 22.4 | 22.0 | 22.0 | 23.5 | 22.8 | 22.7 | 22.9 | 23.8 | 23.4 | 23.2 | 23.6 | 23.5 | 24.7 | 22.8 |
| 6 | 27.6 | 27.7 | 54.0 | 54.4 | 54.5 |  | 24.7 | 21.4 | 21.9 | 22.1 | 20.7 | **23.9** | **20.7** | 20.5 | 20.6 | 19.9 | 20.8 | 21.7 | 22.3 | 22.7 | 22.2 | 22.7 | 25.0 | 21.6 | 19.9 | 20.5 | 20.7 | 21.7 | 20.5 | 20.2 | 21.6 | 21.3 | 22.1 | 20.3 | 21.8 | 20.4 | 21.9 | 20.1 |
| 7 | 24.5 | 24.5 | 24.8 | 24.7 | 24.9 | 24.7 |  | 20.7 | 20.9 | 20.6 | 19.7 | **22.5** | **19.6** | 19.1 | 18.9 | 18.7 | 20.3 | 18.9 | 20.3 | 21.9 | 19.4 | 20.6 | 22.2 | 20.4 | 18.8 | 19.1 | 19.1 | 19.5 | 19.7 | 20.3 | 19.0 | 18.4 | 20.5 | 19.6 | 20.0 | 18.6 | 19.7 | 19.0 |
| 8 | 22.1 | 21.9 | 21.4 | 21.3 | 21.5 | 21.4 | 20.7 |  | 20.9 | 20.9 | 19.5 | **21.8** | **19.6** | 18.9 | 19.2 | 18.9 | 20.4 | 20.0 | 20.7 | 21.5 | 19.9 | 19.5 | 27.5 | 21.3 | 20.9 | 20.1 | 20.8 | 19.8 | 19.6 | 20.7 | 26.1 | 19.5 | 21.8 | 20.7 | 23.5 | 18.9 | 21.6 | 25.8 |
| 9 | 20.5 | 21.2 | 22.0 | 20.9 | 23.0 | 21.9 | 20.9 | 20.9 |  | 24.8 | 19.8 | **22.1** | **21.2** | 21.4 | 21.2 | 20.6 | 22.1 | 21.1 | 19.4 | 21.3 | 20.9 | 21.2 | 22.2 | 19.6 | 18.9 | 19.4 | 19.6 | 19.2 | 20.9 | 23.2 | 20.9 | 19.3 | 27.6 | 19.3 | 22.9 | 20.1 | 20.7 | 21.4 |
| 10 | 20.9 | 21.4 | 22.5 | 21.3 | 23.7 | 22.1 | 20.6 | 20.9 | 24.8 |  | 20.2 | **21.3** | **20.6** | 20.7 | 20.3 | 19.7 | 21.2 | 20.3 | 20.5 | 21.9 | 20.8 | 21.8 | 19.4 | 20.1 | 19.5 | 19.7 | 19.2 | 20.1 | 20.6 | 21.3 | 19.6 | 18.2 | 19.5 | 19.9 | 24.2 | 19.0 | 20.6 | 19.6 |
| 11 | 20.1 | 20.1 | 20.5 | 20.1 | 21.4 | 20.7 | 19.7 | 19.5 | 19.8 | 20.2 |  | **19.9** | **19.6** | 19.2 | 19.2 | 19 | 20.2 | 19.8 | 20.2 | 20.3 | 19.5 | 20.3 | 19.9 | 20 | 19.7 | 19.8 | 19.3 | 19.7 | 19.2 | 20.4 | 18.9 | 20.1 | 19.1 | 19.2 | 24.7 | 18.8 | 20.2 | 20.2 |
| 12 | **22.2** | **23.0** | **24.0** | **22.6** | **24.6** | **23.9** | **22.5** | **21.8** | **22.1** | **21.3** | **19.9** |  | **22.5** | **22.1** | **22.1** | **20.8** | **27.0** | **23.6** | **23.7** | **25.7** | **26.4** | **26.2** | **32.5** | **23.6** | **22.0** | **26.1** | **25.5** | **24.2** | **24.5** | **22.3** | **21.9** | **24.1** | **22.1** | **22.0** | **23.1** | **23.6** | **24.8** | **29.1** |
| 13 | **19.8** | **20.4** | **20.8** | **20.4** | **21.4** | **20.7** | **19.6** | **19.6** | **21.2** | **20.6** | **19.6** | **22.5** |  | **20.5** | **20** | **20** | **21** | **19.5** | **21** | **21.3** | **19.9** | **22.5** | **21.2** | **20.7** | **19.5** | **20.1** | **20.3** | **19** | **22.1** | **19.5** | **18.8** | **19.8** | **18.7** | **18.8** | **20.3** | **19.7** | **20.5** | **19.3** |
| 14 | 19.1 | 19.3 | 20.2 | 19.5 | 21.4 | 20.5 | 19.1 | 18.9 | 21.4 | 20.7 | 19.2 | **22.1** | **20.5** |  | 89.1 | 89.2 | 21.6 | 22.2 | 20.1 | 21.7 | 20.9 | 21.8 | 21.3 | 20.7 | 20.9 | 21.4 | 21.7 | 19.8 | 19.5 | 20.9 | 19.1 | 19.9 | 21.0 | 19.2 | 22.8 | 20.5 | 19.0 | 20.4 |
| 15 | 19.6 | 19.2 | 20.1 | 19.5 | 21.1 | 20.6 | 18.9 | 19.2 | 21.2 | 20.3 | 19.2 | **22.1** | **20** | 89.1 |  | 99.9 | 22.1 | 22.0 | 20.2 | 21.4 | 20.7 | 21.4 | 21.6 | 21.2 | 20.2 | 21.0 | 21.6 | 19.7 | 19.4 | 20.9 | 19.4 | 19.8 | 20.7 | 19.8 | 22.9 | 20.5 | 19.1 | 20.2 |
| 16 | 19.2 | 18.9 | 19.4 | 19.2 | 19.9 | 19.9 | 18.7 | 18.9 | 20.6 | 19.7 | 19 | **20.8** | **20** | 89.2 | 99.9 |  | 20.7 | 21.4 | 19.5 | 20.5 | 19.9 | 20.7 | 20.8 | 20.6 | 19.6 | 20.4 | 21.1 | 19.2 | 18.9 | 20.1 | 18.9 | 19.1 | 19.8 | 19.3 | 21.1 | 19.9 | 18.3 | 19.4 |
| 17 | 20.9 | 21.0 | 21.7 | 20.3 | 23.7 | 20.8 | 20.3 | 20.4 | 22.1 | 21.2 | 20.2 | **27.0** | **21** | 21.6 | 22.1 | 20.7 |  | 22.1 | 20.2 | 20.5 | 20.1 | 20.0 | 20.1 | 20.4 | 21.1 | 21.2 | 20.9 | 20.4 | 20.1 | 20.1 | 20.1 | 20.1 | 20.1 | 19.5 | 19.6 | 19.6 | 19.3 | 19.7 |
| 18 | 19.6 | 19.4 | 21.6 | 19.7 | 22.9 | 21.7 | 18.9 | 20.0 | 21.1 | 20.3 | 19.8 | **23.6** | **19.5** | 22.2 | 22.0 | 21.4 | 22.1 |  | 20.3 | 19.8 | 19.9 | 19.6 | 20.1 | 20.3 | 20.2 | 20.4 | 20.7 | 19.9 | 20.1 | 19.9 | 19.8 | 19.6 | 20.3 | 19.3 | 19.2 | 19.5 | 19.5 | 19.0 |
| 19 | 20.5 | 20.7 | 23.7 | 20.2 | 25.6 | 22.3 | 20.3 | 20.7 | 19.4 | 20.5 | 20.2 | **23.7** | **21** | 20.1 | 20.2 | 19.5 | 20.2 | 20.3 |  | 18.9 | 19.2 | 19.1 | 19.1 | 19.5 | 19.7 | 19.8 | 19.9 | 19.1 | 19.1 | 20.0 | 19.7 | 19.7 | 20.1 | 19.1 | 19.7 | 19.6 | 19.2 | 18.6 |
| 20 | 21.0 | 21.1 | 22.8 | 20.3 | 24.0 | 22.7 | 21.9 | 21.5 | 21.3 | 21.9 | 20.3 | **25.7** | **21.3** | 21.7 | 21.4 | 20.5 | 20.5 | 19.8 | 18.9 |  | 21.8 | 21.0 | 19.7 | 19.5 | 20.4 | 19.9 | 20.6 | 20.1 | 19.2 | 20.8 | 20.1 | 19.9 | 20.1 | 19.7 | 19.9 | 20.0 | 19.1 | 19.4 |
| 21 | 19.4 | 20.7 | 22.5 | 19.6 | 24.9 | 22.2 | 19.4 | 19.9 | 20.9 | 20.8 | 19.5 | **26.4** | **19.9** | 20.9 | 20.7 | 19.9 | 20.1 | 19.9 | 19.2 | 21.8 |  | 22.0 | 20.2 | 19.4 | 19.7 | 19.5 | 20.1 | 19.6 | 19.2 | 20.2 | 20.1 | 19.3 | 19.3 | 18.7 | 19.4 | 19.8 | 20.3 | 18.8 |
| 22 | 19.9 | 20.1 | 20.9 | 19.6 | 24.8 | 22.7 | 20.6 | 19.5 | 21.2 | 21.8 | 20.3 | **26.2** | **22.5** | 21.8 | 21.4 | 20.7 | 20.0 | 19.6 | 19.1 | 21.0 | 22.0 |  | 21.1 | 19.5 | 19.7 | 19.7 | 20.0 | 19.5 | 18.6 | 20.6 | 19.9 | 19.5 | 19.9 | 19.5 | 19.6 | 20.0 | 20.1 | 19.4 |
| 23 | 26.1 | 24.5 | 24.2 | 22.6 | 36.2 | 25.0 | 22.2 | 27.5 | 22.2 | 19.4 | 19.9 | **32.5** | **21.2** | 21.3 | 21.6 | 20.8 | 20.1 | 20.1 | 19.1 | 19.7 | 20.2 | 21.1 |  | 19.1 | 20.0 | 19.5 | 20.7 | 19.3 | 18.9 | 20.2 | 19.8 | 20.1 | 19.6 | 19.8 | 19.9 | 19.3 | 20.1 | 18.8 |
| 24 | 20.2 | 20.6 | 21.2 | 19.6 | 23.7 | 21.6 | 20.4 | 21.3 | 19.6 | 20.1 | 20 | **23.6** | **20.7** | 20.7 | 21.2 | 20.6 | 20.4 | 20.3 | 19.5 | 19.5 | 19.4 | 19.5 | 19.1 |  | 20.4 | 19.8 | 20.6 | 19.4 | 19.9 | 20.3 | 20.4 | 19.7 | 19.3 | 19.3 | 19.1 | 19.0 | 19.4 | 18.8 |
| 25 | 19.4 | 19.3 | 21.4 | 19.2 | 22.4 | 19.9 | 18.8 | 20.9 | 18.9 | 19.5 | 19.7 | **22.0** | **19.5** | 20.9 | 20.2 | 19.6 | 21.1 | 20.2 | 19.7 | 20.4 | 19.7 | 19.7 | 20.0 | 20.4 |  | 28.6 | 21.4 | 20.0 | 19.9 | 20.4 | 20.4 | 20.3 | 19.7 | 19.6 | 19.5 | 19.5 | 19.9 | 19.0 |
| 26 | 19.6 | 19.8 | 20.6 | 19.1 | 22.0 | 20.5 | 19.1 | 20.1 | 19.4 | 19.7 | 19.8 | **26.1** | **20.1** | 21.4 | 21.0 | 20.4 | 21.2 | 20.4 | 19.8 | 19.9 | 19.5 | 19.7 | 19.5 | 19.8 | 28.6 |  | 21.0 | 19.8 | 19.6 | 20.3 | 20.4 | 19.9 | 19.4 | 19.9 | 19.8 | 19.3 | 19.6 | 19.3 |
| 27 | 19.0 | 18.9 | 20.0 | 18.8 | 22.0 | 20.7 | 19.1 | 20.8 | 19.6 | 19.2 | 19.3 | **25.5** | **20.3** | 21.7 | 21.6 | 21.1 | 20.9 | 20.7 | 19.9 | 20.6 | 20.1 | 20.0 | 20.7 | 20.6 | 21.4 | 21.0 |  | 20.6 | 20.4 | 20.6 | 20.6 | 20.4 | 20.5 | 19.8 | 19.3 | 20.1 | 19.9 | 19.4 |
| 28 | 19.0 | 20.0 | 21.5 | 19.6 | 23.5 | 21.7 | 19.5 | 19.8 | 19.2 | 20.1 | 19.7 | **24.2** | **19** | 19.8 | 19.7 | 19.2 | 20.4 | 19.9 | 19.1 | 20.1 | 19.6 | 19.5 | 19.3 | 19.4 | 20.0 | 19.8 | 20.6 |  | 20.0 | 20.3 | 19.6 | 19.1 | 19.5 | 19.2 | 19.5 | 19.5 | 19.8 | 19.1 |
| 29 | 18.8 | 18.9 | 21.0 | 19.0 | 22.8 | 20.5 | 19.7 | 19.6 | 20.9 | 20.6 | 19.2 | **24.5** | **22.1** | 19.5 | 19.4 | 18.9 | 20.1 | 20.1 | 19.1 | 19.2 | 19.2 | 18.6 | 18.9 | 19.9 | 19.9 | 19.6 | 20.4 | 20.0 |  | 20.9 | 20.1 | 19.5 | 19.1 | 18.9 | 19.3 | 19.1 | 19.4 | 19.2 |
| 30 | 20.0 | 21.3 | 20.7 | 19.3 | 22.7 | 20.2 | 20.3 | 20.7 | 23.2 | 21.3 | 20.4 | **22.3** | **19.5** | 20.9 | 20.9 | 20.1 | 20.1 | 19.9 | 20.0 | 20.8 | 20.2 | 20.6 | 20.2 | 20.3 | 20.4 | 20.3 | 20.6 | 20.3 | 20.9 |  | 50.0 | 21.7 | 20.7 | 19.9 | 19.8 | 19.8 | 19.9 | 19.9 |
| 31 | 20.2 | 21.1 | 19.4 | 16.6 | 22.9 | 21.6 | 19.0 | 26.1 | 20.9 | 19.6 | 18.9 | **21.9** | **18.8** | 19.1 | 19.4 | 18.9 | 20.1 | 19.8 | 19.7 | 20.1 | 20.1 | 19.9 | 19.8 | 20.4 | 20.4 | 20.4 | 20.6 | 19.6 | 20.1 | 50.0 |  | 21.6 | 20.9 | 19.5 | 19.6 | 19.8 | 19.5 | 19.8 |
| 32 | 19.8 | 19.2 | 21.5 | 19.7 | 23.8 | 21.3 | 18.4 | 19.5 | 19.3 | 18.2 | 20.1 | **24.1** | **19.8** | 19.9 | 19.8 | 19.1 | 20.1 | 19.6 | 19.7 | 19.9 | 19.3 | 19.5 | 20.1 | 19.7 | 20.3 | 19.9 | 20.4 | 19.1 | 19.5 | 21.7 | 21.6 |  | 20.6 | 19.5 | 19.4 | 19.6 | 19.6 | 19.4 |
| 33 | 20.3 | 22.7 | 21.8 | 20.0 | 23.4 | 22.1 | 20.5 | 21.8 | 27.6 | 19.5 | 19.1 | **22.1** | **18.7** | 21.0 | 20.7 | 19.8 | 20.1 | 20.3 | 20.1 | 20.1 | 19.3 | 19.9 | 19.6 | 19.3 | 19.7 | 19.4 | 20.5 | 19.5 | 19.1 | 20.7 | 20.9 | 20.6 |  | 20.1 | 20.1 | 20.5 | 20.2 | 19.6 |
| 34 | 19.3 | 19.8 | 19.8 | 18.5 | 23.2 | 20.3 | 19.6 | 20.7 | 19.3 | 19.9 | 19.2 | **22.0** | **18.8** | 19.2 | 19.8 | 19.3 | 19.5 | 19.3 | 19.1 | 19.7 | 18.7 | 19.5 | 19.8 | 19.3 | 19.6 | 19.9 | 19.8 | 19.2 | 18.9 | 19.9 | 19.5 | 19.5 | 20.1 |  | 21.0 | 21.0 | 20.0 | 19.3 |
| 35 | 20.5 | 20.1 | 21.4 | 19.8 | 23.6 | 21.8 | 20.0 | 23.5 | 22.9 | 24.2 | 24.7 | **23.1** | **20.3** | 22.8 | 22.9 | 21.1 | 19.6 | 19.2 | 19.7 | 19.9 | 19.4 | 19.6 | 19.9 | 19.1 | 19.5 | 19.8 | 19.3 | 19.5 | 19.3 | 19.8 | 19.6 | 19.4 | 20.1 | 21.0 |  | 20.9 | 19.8 | 19.5 |
| 36 | 18.3 | 19.4 | 21.5 | 19.2 | 23.5 | 20.4 | 18.6 | 18.9 | 20.1 | 19.0 | 18.8 | **23.6** | **19.7** | 20.5 | 20.5 | 19.9 | 19.6 | 19.5 | 19.6 | 20.0 | 19.8 | 20.0 | 19.3 | 19.0 | 19.5 | 19.3 | 20.1 | 19.5 | 19.1 | 19.8 | 19.8 | 19.6 | 20.5 | 21.0 | 20.9 |  | 20.3 | 19.4 |
| 37 | 19.8 | 20.8 | 22.3 | 20.3 | 24.7 | 21.9 | 19.7 | 21.6 | 20.7 | 20.6 | 20.2 | **24.8** | **20.5** | 19.0 | 19.1 | 18.3 | 19.3 | 19.5 | 19.2 | 19.1 | 20.3 | 20.1 | 20.1 | 19.4 | 19.9 | 19.6 | 19.9 | 19.8 | 19.4 | 19.9 | 19.5 | 19.6 | 20.2 | 20.0 | 19.8 | 20.3 |  | 20.2 |
| 38 | 19.8 | 19.7 | 20.7 | 18.9 | 22.8 | 20.1 | 19.0 | 25.8 | 21.4 | 19.6 | 20.2 | **29.1** | **19.3** | 20.4 | 20.2 | 19.4 | 19.7 | 19.0 | 18.6 | 19.4 | 18.8 | 19.4 | 18.8 | 18.8 | 19.0 | 19.3 | 19.4 | 19.1 | 19.2 | 19.9 | 19.8 | 19.4 | 19.6 | 19.3 | 19.5 | 19.4 | 20.2 |  |
