# Supplementary material for: Genome-based reclassification of the family Stappiaceae and assessment of environmental forcing with the report of two novel taxa, Flexibacterium corallicola gen. nov., sp. nov., and Nesiotobacter zosterae sp. nov., isolated from coral and seagrass
Source: PLoS One. 2025 May 15;20(5):e0322500. doi: 10.1371/journal.pone.0322500 (PMC12080928; doi:10.1371/journal.pone.0322500)
Supplement: S6 Table — (DOCX) [file pone.0322500.s011.docx]

**S6 Table. AAI values between genomes.**

| AAI | 1 | 2 | 3 | 4 | 5 | 6 | 7 | 8 | 9 | 10 | 11 | 12 | 13 | 14 | 15 | 16 | 17 | 18 | 19 | 20 | 21 | 22 | 23 | 24 | 25 | 26 | 27 | 28 | 29 | 30 | 31 | 32 | 33 | 34 | 35 | 36 | 37 | 38 |
| --- | --- | --- | --- | --- | --- | --- | --- | --- | --- | --- | --- | --- | --- | --- | --- | --- | --- | --- | --- | --- | --- | --- | --- | --- | --- | --- | --- | --- | --- | --- | --- | --- | --- | --- | --- | --- | --- | --- |
| 1 |  | 96.58 | 90.26 | 90.11 | 90.42 | 90.21 | 85.01 | 82.59 | 62.50 | 62.67 | 64.68 | **63.23** | **64.82** | 64.25 | 64.25 | 64.28 | 56.59 | 56.79 | 56.98 | 57.04 | 57.64 | 57.20 | 57.73 | 57.60 | 57.34 | 57.44 | 57.96 | 57.53 | 57.90 | 57.59 | 58.21 | 57.27 | 58.18 | 58.73 | 57.79 | 57.65 | 58.68 | 58.59 |
| 2 | 96.58 |  | 90.57 | 90.55 | 90.65 | 90.72 | 85.39 | 82.72 | 62.65 | 62.75 | 64.58 | **63.36** | **64.88** | 64.34 | 64.46 | 64.51 | 57.12 | 56.98 | 57.15 | 57.19 | 57.70 | 57.36 | 57.95 | 57.66 | 57.49 | 57.66 | 57.87 | 57.83 | 57.96 | 57.95 | 58.30 | 57.76 | 58.37 | 58.54 | 57.76 | 57.85 | 58.78 | 58.80 |
| 3 | 90.26 | 90.57 |  | 97.59 | 97.65 | 96.60 | 85.50 | 82.64 | 62.86 | 63.17 | 65.02 | **63.43** | **65.46** | 64.86 | 64.95 | 64.99 | 57.11 | 57.14 | 57.29 | 57.42 | 57.80 | 57.56 | 58.14 | 57.83 | 57.53 | 57.82 | 58.20 | 57.81 | 58.24 | 58.11 | 58.38 | 57.91 | 58.75 | 58.95 | 57.95 | 58.02 | 59.15 | 58.92 |
| 4 | 90.11 | 90.55 | 97.59 |  | 97.52 | 96.65 | 85.64 | 82.74 | 62.76 | 63.05 | 64.83 | **63.37** | **65.41** | 64.69 | 64.87 | 64.85 | 57.09 | 57.23 | 57.21 | 57.33 | 57.76 | 57.57 | 58.19 | 57.91 | 57.54 | 57.86 | 58.14 | 57.85 | 58.45 | 58.04 | 58.31 | 57.97 | 58.62 | 59.01 | 57.99 | 57.97 | 59.16 | 58.91 |
| 5 | 90.42 | 90.65 | 97.65 | 97.52 |  | 96.87 | 85.78 | 83.06 | 62.92 | 63.52 | 64.84 | **63.82** | **65.68** | 64.79 | 64.90 | 64.99 | 57.38 | 57.30 | 57.35 | 57.58 | 57.84 | 57.60 | 58.27 | 57.94 | 57.67 | 57.92 | 58.29 | 57.94 | 58.23 | 58.07 | 58.47 | 58.05 | 58.99 | 59.11 | 58.29 | 58.09 | 59.44 | 59.23 |
| 6 | 90.21 | 90.72 | 96.60 | 96.65 | 96.87 |  | 85.39 | 82.61 | 62.72 | 62.92 | 64.85 | **63.43** | **65.03** | 64.58 | 64.73 | 64.77 | 57.02 | 57.04 | 57.26 | 57.15 | 57.76 | 57.36 | 58.02 | 57.73 | 57.38 | 57.67 | 58.02 | 57.69 | 58.12 | 57.75 | 58.19 | 57.90 | 58.38 | 58.73 | 57.82 | 57.75 | 59.19 | 58.94 |
| 7 | 85.01 | 85.39 | 85.50 | 85.64 | 85.78 | 85.39 |  | 80.70 | 62.86 | 62.74 | 64.71 | **63.48** | **64.95** | 64.23 | 64.38 | 64.37 | 57.26 | 57.57 | 57.37 | 57.34 | 57.74 | 57.71 | 58.30 | 57.83 | 57.92 | 58.01 | 58.28 | 58.11 | 58.36 | 58.03 | 58.12 | 57.80 | 58.63 | 58.92 | 58.14 | 57.96 | 59.38 | 59.11 |
| 8 | 82.59 | 82.72 | 82.64 | 82.74 | 83.06 | 82.61 | 80.70 |  | 62.58 | 62.68 | 64.51 | **64.13** | **64.72** | 64.03 | 64.25 | 64.22 | 57.12 | 56.95 | 56.87 | 57.00 | 57.51 | 57.50 | 58.05 | 57.54 | 57.61 | 58.13 | 57.75 | 57.85 | 58.20 | 57.75 | 58.18 | 57.48 | 58.27 | 58.76 | 58.02 | 57.78 | 59.00 | 58.77 |
| 9 | 62.50 | 62.65 | 62.86 | 62.76 | 62.92 | 62.72 | 62.86 | 62.58 |  | 68.50 | 63.08 | **61.90** | **63.17** | 62.98 | 63.08 | 63.11 | 56.81 | 56.89 | 56.73 | 56.63 | 57.05 | 56.73 | 57.00 | 56.97 | 57.11 | 57.40 | 57.47 | 56.86 | 57.61 | 56.99 | 57.45 | 56.91 | 57.67 | 57.73 | 57.02 | 57.25 | 58.17 | 57.81 |
| 10 | 62.67 | 62.75 | 63.17 | 63.05 | 63.52 | 62.92 | 62.74 | 62.68 | 68.50 |  | 63.23 | **61.87** | **63.24** | 63.04 | 63.35 | 63.35 | 57.36 | 57.37 | 57.63 | 57.28 | 58.19 | 57.81 | 57.94 | 57.74 | 57.86 | 57.78 | 58.32 | 57.87 | 58.37 | 57.88 | 58.25 | 57.79 | 58.51 | 58.62 | 57.94 | 57.91 | 58.67 | 58.73 |
| 11 | 64.68 | 64.58 | 65.02 | 64.83 | 64.84 | 64.85 | 64.71 | 64.51 | 63.08 | 63.23 |  | **66.82** | **66.33** | 66.22 | 66.31 | 66.30 | 58.42 | 58.25 | 58.08 | 58.44 | 58.46 | 58.44 | 58.99 | 58.78 | 58.77 | 59.00 | 58.96 | 58.67 | 59.05 | 58.63 | 59.03 | 58.77 | 59.65 | 59.52 | 58.64 | 58.73 | 60.09 | 59.79 |
| 12 | **63.23** | **63.36** | **63.43** | **63.37** | **63.82** | **63.43** | **63.48** | **64.13** | **61.90** | **61.87** | **66.82** |  | **64.26** | **63.85** | **64.09** | **63.97** | **57.39** | **57.07** | **56.77** | **57.31** | **57.39** | **57.52** | **57.81** | **57.40** | **57.35** | **57.69** | **57.95** | **57.49** | **57.88** | **57.75** | **57.98** | **57.60** | **57.99** | **58.12** | **57.73** | **57.69** | **58.59** | **58.42** |
| 13 | **64.82** | **64.88** | **65.46** | **65.41** | **65.68** | **65.03** | **64.95** | **64.72** | **63.17** | **63.24** | **66.33** | **64.26** |  | **80.54** | **80.33** | **80.34** | **59.22** | **59.17** | **58.74** | **59.28** | **59.44** | **59.75** | **59.65** | **59.06** | **59.57** | **59.57** | **59.54** | **59.20** | **60.23** | **59.41** | **59.72** | **59.44** | **60.43** | **60.05** | **59.38** | **59.65** | **60.51** | **60.42** |
| 14 | 64.25 | 64.34 | 64.86 | 64.69 | 64.79 | 64.58 | 64.23 | 64.03 | 62.98 | 63.04 | 66.22 | **63.85** | **80.54** |  | 99.02 | 98.99 | 59.11 | 58.96 | 58.34 | 58.79 | 59.33 | 59.31 | 59.34 | 58.93 | 59.17 | 59.23 | 59.26 | 58.95 | 59.80 | 59.13 | 59.44 | 59.25 | 60.28 | 60.19 | 59.43 | 59.40 | 60.22 | 60.35 |
| 15 | 64.25 | 64.46 | 64.95 | 64.87 | 64.90 | 64.73 | 64.38 | 64.25 | 63.08 | 63.35 | 66.31 | **64.09** | **80.33** | 99.02 |  | 99.95 | 59.21 | 59.12 | 58.55 | 58.98 | 59.35 | 59.51 | 59.52 | 59.19 | 59.36 | 59.36 | 59.38 | 59.32 | 60.08 | 59.15 | 59.52 | 59.59 | 60.67 | 60.30 | 59.59 | 59.48 | 60.54 | 60.55 |
| 16 | 64.28 | 64.51 | 64.99 | 64.85 | 64.99 | 64.77 | 64.37 | 64.22 | 63.11 | 63.35 | 66.30 | **63.97** | **80.34** | 98.99 | 99.95 |  | 59.21 | 59.09 | 58.54 | 58.92 | 59.36 | 59.46 | 59.45 | 59.12 | 59.34 | 59.38 | 59.35 | 59.30 | 60.05 | 59.07 | 59.49 | 59.52 | 60.63 | 60.26 | 59.60 | 59.47 | 60.49 | 60.51 |
| 17 | 56.59 | 57.12 | 57.11 | 57.09 | 57.38 | 57.02 | 57.26 | 57.12 | 56.81 | 57.36 | 58.42 | **57.39** | **59.22** | 59.11 | 59.21 | 59.21 |  | 78.42 | 75.90 | 73.96 | 75.42 | 69.50 | 68.79 | 68.71 | 67.94 | 68.34 | 67.43 | 67.84 | 69.78 | 64.41 | 65.11 | 64.70 | 66.35 | 62.55 | 61.76 | 62.16 | 62.58 | 62.82 |
| 18 | 56.79 | 56.98 | 57.14 | 57.23 | 57.30 | 57.04 | 57.57 | 56.95 | 56.89 | 57.37 | 58.25 | **57.07** | **59.17** | 58.96 | 59.12 | 59.09 | 78.42 |  | 75.03 | 73.21 | 74.61 | 69.16 | 69.00 | 67.58 | 67.71 | 68.40 | 67.22 | 67.74 | 69.73 | 63.97 | 64.80 | 64.82 | 66.83 | 62.49 | 61.74 | 61.77 | 62.81 | 62.58 |
| 19 | 56.98 | 57.15 | 57.29 | 57.21 | 57.35 | 57.26 | 57.37 | 56.87 | 56.73 | 57.63 | 58.08 | **56.77** | **58.74** | 58.34 | 58.55 | 58.54 | 75.90 | 75.03 |  | 73.76 | 74.67 | 69.04 | 68.27 | 68.23 | 65.34 | 65.90 | 65.52 | 65.77 | 68.81 | 63.26 | 63.70 | 63.60 | 64.96 | 61.45 | 61.29 | 60.78 | 61.85 | 62.12 |
| 20 | 57.04 | 57.19 | 57.42 | 57.33 | 57.58 | 57.15 | 57.34 | 57.00 | 56.63 | 57.28 | 58.44 | **57.31** | **59.28** | 58.79 | 58.98 | 58.92 | 73.96 | 73.21 | 73.76 |  | 76.70 | 70.56 | 69.45 | 68.17 | 66.38 | 66.46 | 65.93 | 66.05 | 69.55 | 63.46 | 64.01 | 63.98 | 65.62 | 61.61 | 61.80 | 61.10 | 62.29 | 62.20 |
| 21 | 57.64 | 57.70 | 57.80 | 57.76 | 57.84 | 57.76 | 57.74 | 57.51 | 57.05 | 58.19 | 58.46 | **57.39** | **59.44** | 59.33 | 59.35 | 59.36 | 75.42 | 74.61 | 74.67 | 76.70 |  | 71.79 | 70.03 | 68.51 | 67.50 | 67.42 | 66.45 | 66.48 | 69.87 | 64.38 | 64.78 | 64.73 | 66.42 | 62.14 | 62.28 | 61.71 | 62.75 | 62.80 |
| 22 | 57.20 | 57.36 | 57.56 | 57.57 | 57.60 | 57.36 | 57.71 | 57.50 | 56.73 | 57.81 | 58.44 | **57.52** | **59.75** | 59.31 | 59.51 | 59.46 | 69.50 | 69.16 | 69.04 | 70.56 | 71.79 |  | 71.48 | 69.23 | 67.60 | 67.61 | 66.60 | 67.01 | 70.41 | 64.42 | 64.94 | 65.28 | 66.77 | 62.61 | 62.79 | 62.15 | 63.27 | 63.16 |
| 23 | 57.73 | 57.95 | 58.14 | 58.19 | 58.27 | 58.02 | 58.30 | 58.05 | 57.00 | 57.94 | 58.99 | **57.81** | **59.65** | 59.34 | 59.52 | 59.45 | 68.79 | 69.00 | 68.27 | 69.45 | 70.03 | 71.48 |  | 70.42 | 67.66 | 67.94 | 67.01 | 66.88 | 69.75 | 64.98 | 65.40 | 65.89 | 67.27 | 63.19 | 62.66 | 62.78 | 63.42 | 63.25 |
| 24 | 57.60 | 57.66 | 57.83 | 57.91 | 57.94 | 57.73 | 57.83 | 57.54 | 56.97 | 57.74 | 58.78 | **57.40** | **59.06** | 58.93 | 59.19 | 59.12 | 68.71 | 67.58 | 68.23 | 68.17 | 68.51 | 69.23 | 70.42 |  | 66.95 | 66.91 | 66.40 | 65.91 | 69.17 | 64.00 | 64.35 | 64.30 | 65.75 | 62.52 | 62.11 | 61.79 | 62.58 | 62.99 |
| 25 | 57.34 | 57.49 | 57.53 | 57.54 | 57.67 | 57.38 | 57.92 | 57.61 | 57.11 | 57.86 | 58.77 | **57.35** | **59.57** | 59.17 | 59.36 | 59.34 | 67.94 | 67.71 | 65.34 | 66.38 | 67.50 | 67.60 | 67.66 | 66.95 |  | 89.41 | 69.60 | 69.12 | 71.57 | 66.68 | 66.96 | 65.67 | 67.25 | 62.55 | 61.60 | 62.58 | 63.43 | 62.50 |
| 26 | 57.44 | 57.66 | 57.82 | 57.86 | 57.92 | 57.67 | 58.01 | 58.13 | 57.40 | 57.78 | 59.00 | **57.69** | **59.57** | 59.23 | 59.36 | 59.38 | 68.34 | 68.40 | 65.90 | 66.46 | 67.42 | 67.61 | 67.94 | 66.91 | 89.41 |  | 69.66 | 69.28 | 71.92 | 66.72 | 67.27 | 65.81 | 66.99 | 62.67 | 61.69 | 62.62 | 63.41 | 62.64 |
| 27 | 57.96 | 57.87 | 58.20 | 58.14 | 58.29 | 58.02 | 58.28 | 57.75 | 57.47 | 58.32 | 58.96 | **57.95** | **59.54** | 59.26 | 59.38 | 59.35 | 67.43 | 67.22 | 65.52 | 65.93 | 66.45 | 66.60 | 67.01 | 66.40 | 69.60 | 69.66 |  | 77.33 | 69.96 | 65.52 | 65.97 | 65.55 | 67.76 | 62.95 | 62.14 | 62.66 | 63.09 | 62.56 |
| 28 | 57.53 | 57.83 | 57.81 | 57.85 | 57.94 | 57.69 | 58.11 | 57.85 | 56.86 | 57.87 | 58.67 | **57.49** | **59.20** | 58.95 | 59.32 | 59.30 | 67.84 | 67.74 | 65.77 | 66.05 | 66.48 | 67.01 | 66.88 | 65.91 | 69.12 | 69.28 | 77.33 |  | 70.75 | 64.13 | 64.48 | 64.45 | 66.23 | 61.67 | 61.41 | 61.84 | 62.46 | 62.26 |
| 29 | 57.90 | 57.96 | 58.24 | 58.45 | 58.23 | 58.12 | 58.36 | 58.20 | 57.61 | 58.37 | 59.05 | **57.88** | **60.23** | 59.80 | 60.08 | 60.05 | 69.78 | 69.73 | 68.81 | 69.55 | 69.87 | 70.41 | 69.75 | 69.17 | 71.57 | 71.92 | 69.96 | 70.75 |  | 65.81 | 66.02 | 66.37 | 68.97 | 63.04 | 62.91 | 62.81 | 63.89 | 63.75 |
| 30 | 57.59 | 57.95 | 58.11 | 58.04 | 58.07 | 57.75 | 58.03 | 57.75 | 56.99 | 57.88 | 58.63 | **57.75** | **59.41** | 59.13 | 59.15 | 59.07 | 64.41 | 63.97 | 63.26 | 63.46 | 64.38 | 64.42 | 64.98 | 64.00 | 66.68 | 66.72 | 65.52 | 64.13 | 65.81 |  | 95.14 | 74.14 | 70.14 | 63.12 | 62.24 | 63.39 | 63.70 | 62.76 |
| 31 | 58.21 | 58.30 | 58.38 | 58.31 | 58.47 | 58.19 | 58.12 | 58.18 | 57.45 | 58.25 | 59.03 | **57.98** | **59.72** | 59.44 | 59.52 | 59.49 | 65.11 | 64.80 | 63.70 | 64.01 | 64.78 | 64.94 | 65.40 | 64.35 | 66.96 | 67.27 | 65.97 | 64.48 | 66.02 | 95.14 |  | 74.07 | 71.19 | 63.51 | 62.84 | 63.71 | 64.05 | 63.30 |
| 32 | 57.27 | 57.76 | 57.91 | 57.97 | 58.05 | 57.90 | 57.80 | 57.48 | 56.91 | 57.79 | 58.77 | **57.60** | **59.44** | 59.25 | 59.59 | 59.52 | 64.70 | 64.82 | 63.60 | 63.98 | 64.73 | 65.28 | 65.89 | 64.30 | 65.67 | 65.81 | 65.55 | 64.45 | 66.37 | 74.14 | 74.07 |  | 71.81 | 63.61 | 63.06 | 63.32 | 63.91 | 63.28 |
| 33 | 58.18 | 58.37 | 58.75 | 58.62 | 58.99 | 58.38 | 58.63 | 58.27 | 57.67 | 58.51 | 59.65 | **57.99** | **60.43** | 60.28 | 60.67 | 60.63 | 66.35 | 66.83 | 64.96 | 65.62 | 66.42 | 66.77 | 67.27 | 65.75 | 67.25 | 66.99 | 67.76 | 66.23 | 68.97 | 70.14 | 71.19 | 71.81 |  | 65.61 | 64.93 | 65.63 | 66.01 | 65.46 |
| 34 | 58.73 | 58.54 | 58.95 | 59.01 | 59.11 | 58.73 | 58.92 | 58.76 | 57.73 | 58.62 | 59.52 | **58.12** | **60.05** | 60.19 | 60.30 | 60.26 | 62.55 | 62.49 | 61.45 | 61.61 | 62.14 | 62.61 | 63.19 | 62.52 | 62.55 | 62.67 | 62.95 | 61.67 | 63.04 | 63.12 | 63.51 | 63.61 | 65.61 |  | 74.76 | 72.89 | 68.57 | 67.72 |
| 35 | 57.79 | 57.76 | 57.95 | 57.99 | 58.29 | 57.82 | 58.14 | 58.02 | 57.02 | 57.94 | 58.64 | **57.73** | **59.38** | 59.43 | 59.59 | 59.60 | 61.76 | 61.74 | 61.29 | 61.80 | 62.28 | 62.79 | 62.66 | 62.11 | 61.60 | 61.69 | 62.14 | 61.41 | 62.91 | 62.24 | 62.84 | 63.06 | 64.93 | 74.76 |  | 74.07 | 67.64 | 67.53 |
| 36 | 57.65 | 57.85 | 58.02 | 57.97 | 58.09 | 57.75 | 57.96 | 57.78 | 57.25 | 57.91 | 58.73 | **57.69** | **59.65** | 59.40 | 59.48 | 59.47 | 62.16 | 61.77 | 60.78 | 61.10 | 61.71 | 62.15 | 62.78 | 61.79 | 62.58 | 62.62 | 62.66 | 61.84 | 62.81 | 63.39 | 63.71 | 63.32 | 65.63 | 72.89 | 74.07 |  | 67.56 | 66.86 |
| 37 | 58.68 | 58.78 | 59.15 | 59.16 | 59.44 | 59.19 | 59.38 | 59.00 | 58.17 | 58.67 | 60.09 | **58.59** | **60.51** | 60.22 | 60.54 | 60.49 | 62.58 | 62.81 | 61.85 | 62.29 | 62.75 | 63.27 | 63.42 | 62.58 | 63.43 | 63.41 | 63.09 | 62.46 | 63.89 | 63.70 | 64.05 | 63.91 | 66.01 | 68.57 | 67.64 | 67.56 |  | 73.88 |
| 38 | 58.59 | 58.80 | 58.92 | 58.91 | 59.23 | 58.94 | 59.11 | 58.77 | 57.81 | 58.73 | 59.79 | **58.42** | **60.42** | 60.35 | 60.55 | 60.51 | 62.82 | 62.58 | 62.12 | 62.20 | 62.80 | 63.16 | 63.25 | 62.99 | 62.50 | 62.64 | 62.56 | 62.26 | 63.75 | 62.76 | 63.30 | 63.28 | 65.46 | 67.72 | 67.53 | 66.86 | 73.88 |  |
