# Supplementary material for: Genome-based reclassification of the family Stappiaceae and assessment of environmental forcing with the report of two novel taxa, Flexibacterium corallicola gen. nov., sp. nov., and Nesiotobacter zosterae sp. nov., isolated from coral and seagrass
Source: PLoS One. 2025 May 15;20(5):e0322500. doi: 10.1371/journal.pone.0322500 (PMC12080928; doi:10.1371/journal.pone.0322500)
Supplement: S7 Table — (DOCX) [file pone.0322500.s012.docx]

**S7 Table. POCP values between genomes.**

| POCP | 1 | 2 | 3 | 4 | 5 | 6 | 7 | 8 | 9 | 10 | 11 | 12 | 13 | 14 | 15 | 16 | 17 | 18 | 19 | 20 | 21 | 22 | 23 | 24 | 25 | 26 | 27 | 28 | 29 | 30 | 31 | 32 | 33 | 34 | 35 | 36 | 37 | 38 |
| --- | --- | --- | --- | --- | --- | --- | --- | --- | --- | --- | --- | --- | --- | --- | --- | --- | --- | --- | --- | --- | --- | --- | --- | --- | --- | --- | --- | --- | --- | --- | --- | --- | --- | --- | --- | --- | --- | --- |
| 1 |  | 88.79 | 85.44 | 83.87 | 84.73 | 85.31 | 73.09 | 73.71 | 53.67 | 55.65 | 58.03 | **54.22** | **59.37** | 57.76 | 57.62 | 57.49 | 47.17 | 46.99 | 46.36 | 47.81 | 48.07 | 47.68 | 48.16 | 49.23 | 47.36 | 47.78 | 49.82 | 47.93 | 49.40 | 48.42 | 48.95 | 49.32 | 46.85 | 50.48 | 48.24 | 48.44 | 48.30 | 49.69 |
| 2 | 88.79 |  | 88.61 | 86.84 | 88.28 | 87.66 | 75.58 | 74.54 | 56.07 | 57.08 | 60.26 | **53.68** | **60.81** | 58.91 | 59.49 | 59.42 | 47.83 | 48.02 | 46.98 | 49.27 | 49.41 | 49.46 | 49.85 | 50.43 | 49.15 | 49.36 | 51.36 | 49.57 | 51.13 | 50.28 | 50.45 | 51.02 | 48.09 | 51.76 | 49.40 | 50.44 | 50.13 | 51.65 |
| 3 | 85.44 | 88.61 |  | 90.97 | 92.34 | 89.73 | 75.30 | 73.48 | 56.13 | 56.15 | 60.41 | **53.80** | **61.27** | 59.30 | 59.60 | 59.43 | 47.85 | 48.30 | 47.60 | 49.44 | 49.82 | 50.00 | 49.87 | 50.26 | 49.24 | 48.88 | 51.42 | 49.46 | 51.17 | 50.08 | 50.29 | 51.16 | 47.98 | 51.99 | 49.71 | 50.51 | 50.44 | 51.67 |
| 4 | 83.87 | 86.84 | 90.97 |  | 91.11 | 88.71 | 74.46 | 72.89 | 55.56 | 55.59 | 59.38 | **53.16** | **60.32** | 58.56 | 59.31 | 59.20 | 47.63 | 48.02 | 47.04 | 48.73 | 49.16 | 49.24 | 49.50 | 49.70 | 48.94 | 48.66 | 50.73 | 48.95 | 50.98 | 49.85 | 50.00 | 50.81 | 47.54 | 51.46 | 49.04 | 49.94 | 49.84 | 51.20 |
| 5 | 84.73 | 88.28 | 92.34 | 91.11 |  | 88.92 | 76.44 | 73.77 | 56.94 | 56.43 | 61.52 | **54.24** | **61.96** | 59.91 | 60.71 | 60.55 | 48.39 | 48.95 | 47.84 | 49.77 | 50.32 | 50.36 | 50.91 | 51.09 | 50.04 | 49.75 | 52.30 | 50.18 | 52.26 | 51.06 | 51.41 | 52.24 | 48.98 | 52.93 | 50.32 | 51.33 | 51.20 | 52.45 |
| 6 | 85.31 | 87.66 | 89.73 | 88.71 | 88.92 |  | 74.37 | 74.62 | 55.20 | 56.38 | 59.21 | **53.61** | **60.02** | 58.03 | 58.59 | 58.48 | 48.10 | 48.16 | 47.35 | 48.81 | 49.30 | 49.13 | 49.55 | 50.25 | 48.54 | 48.81 | 50.73 | 48.85 | 51.04 | 49.46 | 50.05 | 50.60 | 47.70 | 51.35 | 48.93 | 49.63 | 49.49 | 50.86 |
| 7 | 73.09 | 75.58 | 75.30 | 74.46 | 76.44 | 74.37 |  | 75.96 | 58.23 | 57.42 | 62.53 | **57.03** | **62.94** | 60.39 | 61.24 | 61.16 | 47.75 | 49.01 | 47.11 | 50.45 | 51.17 | 51.78 | 52.47 | 50.70 | 51.37 | 50.95 | 53.53 | 51.16 | 52.86 | 51.93 | 52.46 | 52.40 | 50.13 | 54.03 | 51.69 | 52.23 | 52.40 | 53.48 |
| 8 | 73.71 | 74.54 | 73.48 | 72.89 | 73.77 | 74.62 | 75.96 |  | 57.50 | 57.55 | 61.03 | **59.83** | **62.91** | 60.35 | 61.08 | 60.88 | 48.17 | 48.68 | 46.23 | 49.96 | 51.09 | 50.72 | 50.91 | 50.31 | 50.04 | 51.15 | 52.13 | 50.64 | 52.23 | 51.02 | 52.06 | 51.62 | 49.84 | 53.39 | 50.75 | 51.38 | 51.65 | 52.24 |
| 9 | 53.67 | 56.07 | 56.13 | 55.56 | 56.94 | 55.20 | 58.23 | 57.50 |  | 72.13 | 62.65 | **52.61** | **63.36** | 63.22 | 63.77 | 63.65 | 44.88 | 46.24 | 41.98 | 46.43 | 49.27 | 48.95 | 51.18 | 48.40 | 49.81 | 49.85 | 52.86 | 51.13 | 51.19 | 52.24 | 53.70 | 52.15 | 50.29 | 52.24 | 50.36 | 49.94 | 51.66 | 51.19 |
| 10 | 55.65 | 57.08 | 56.15 | 55.59 | 56.43 | 56.38 | 57.42 | 57.55 | 72.13 |  | 61.80 | **50.96** | **61.10** | 60.33 | 61.06 | 60.88 | 46.13 | 46.96 | 43.57 | 47.51 | 50.32 | 49.77 | 52.19 | 50.39 | 50.19 | 51.17 | 53.20 | 52.16 | 52.11 | 52.28 | 53.89 | 52.81 | 50.64 | 52.29 | 50.33 | 49.99 | 52.13 | 51.78 |
| 11 | 58.03 | 60.26 | 60.41 | 59.38 | 61.52 | 59.21 | 62.53 | 61.03 | 62.65 | 61.80 |  | **56.34** | **68.58** | 68.76 | 70.05 | 69.87 | 48.19 | 50.40 | 46.15 | 51.33 | 53.06 | 53.63 | 55.13 | 52.57 | 53.32 | 53.13 | 56.93 | 55.12 | 55.81 | 55.03 | 56.98 | 54.94 | 53.73 | 56.54 | 55.11 | 54.54 | 56.04 | 57.20 |
| 12 | **54.22** | **53.68** | **53.80** | **53.16** | **54.24** | **53.61** | **57.03** | **59.83** | **52.61** | **50.96** | **56.34** |  | **56.40** | **55.06** | **55.37** | **54.94** | **43.90** | **43.06** | **41.27** | **44.61** | **46.21** | **46.56** | **47.14** | **45.09** | **45.36** | **46.65** | **47.30** | **45.14** | **46.73** | **46.04** | **47.51** | **45.91** | **46.37** | **47.34** | **45.87** | **45.32** | **46.73** | **46.88** |
| 13 | **59.37** | **60.81** | **61.27** | **60.32** | **61.96** | **60.02** | **62.94** | **62.91** | **63.36** | **61.10** | **68.58** | **56.40** |  | **84.31** | **83.57** | **83.32** | **49.48** | **51.38** | **46.97** | **53.39** | **55.61** | **56.25** | **56.42** | **52.49** | **54.20** | **54.19** | **57.22** | **55.56** | **56.73** | **55.47** | **56.34** | **56.34** | **54.00** | **56.73** | **55.03** | **55.49** | **56.62** | **57.29** |
| 14 | 57.76 | 58.91 | 59.30 | 58.56 | 59.91 | 58.03 | 60.39 | 60.35 | 63.22 | 60.33 | 68.76 | **55.06** | **84.31** |  | 94.03 | 93.72 | 49.50 | 50.42 | 45.89 | 51.46 | 53.83 | 54.27 | 55.00 | 51.34 | 53.04 | 53.26 | 56.12 | 54.68 | 55.90 | 54.79 | 56.15 | 55.21 | 53.41 | 55.80 | 54.54 | 54.23 | 55.20 | 56.56 |
| 15 | 57.62 | 59.49 | 59.60 | 59.31 | 60.71 | 58.59 | 61.24 | 61.08 | 63.77 | 61.06 | 70.05 | **55.37** | **83.57** | 94.03 |  | 99.02 | 50.29 | 51.14 | 46.46 | 51.93 | 54.75 | 55.15 | 55.72 | 51.95 | 53.92 | 54.09 | 57.23 | 55.63 | 56.64 | 55.79 | 56.91 | 56.16 | 54.57 | 56.82 | 55.66 | 55.24 | 56.29 | 57.74 |
| 16 | 57.49 | 59.42 | 59.43 | 59.20 | 60.55 | 58.48 | 61.16 | 60.88 | 63.65 | 60.88 | 69.87 | **54.94** | **83.32** | 93.72 | 99.02 |  | 49.91 | 50.93 | 46.46 | 51.78 | 54.60 | 55.04 | 55.54 | 51.80 | 53.80 | 53.99 | 57.06 | 55.49 | 56.49 | 55.66 | 56.86 | 55.94 | 54.45 | 56.59 | 55.39 | 55.15 | 56.21 | 57.58 |
| 17 | 47.17 | 47.83 | 47.85 | 47.63 | 48.39 | 48.10 | 47.75 | 48.17 | 44.88 | 46.13 | 48.19 | **43.90** | **49.48** | 49.50 | 50.29 | 49.91 |  | 69.15 | 65.88 | 64.32 | 65.64 | 61.53 | 59.28 | 61.61 | 60.43 | 61.60 | 59.85 | 60.59 | 63.75 | 57.43 | 57.58 | 58.50 | 55.92 | 54.99 | 55.42 | 55.58 | 55.33 | 57.75 |
| 18 | 46.99 | 48.02 | 48.30 | 48.02 | 48.95 | 48.16 | 49.01 | 48.68 | 46.24 | 46.96 | 50.40 | **43.06** | **51.38** | 50.42 | 51.14 | 50.93 | 69.15 |  | 63.06 | 63.02 | 65.78 | 62.12 | 61.47 | 59.86 | 62.37 | 63.29 | 62.91 | 63.73 | 66.28 | 58.07 | 58.93 | 59.51 | 59.92 | 56.84 | 56.49 | 57.31 | 56.89 | 57.67 |
| 19 | 46.36 | 46.98 | 47.60 | 47.04 | 47.84 | 47.35 | 47.11 | 46.23 | 41.98 | 43.57 | 46.15 | **41.27** | **46.97** | 45.89 | 46.46 | 46.46 | 65.88 | 63.06 |  | 64.19 | 64.64 | 59.64 | 56.12 | 61.40 | 54.87 | 55.21 | 54.90 | 56.12 | 60.47 | 52.82 | 53.63 | 54.37 | 51.41 | 52.67 | 52.99 | 52.68 | 52.07 | 55.58 |
| 20 | 47.81 | 49.27 | 49.44 | 48.73 | 49.77 | 48.81 | 50.45 | 49.96 | 46.43 | 47.51 | 51.33 | **44.61** | **53.39** | 51.46 | 51.93 | 51.78 | 64.32 | 63.02 | 64.19 |  | 76.56 | 71.76 | 67.61 | 63.19 | 58.51 | 58.59 | 62.08 | 61.01 | 68.41 | 57.66 | 59.20 | 60.06 | 57.74 | 57.56 | 59.25 | 57.02 | 56.23 | 59.11 |
| 21 | 48.07 | 49.41 | 49.82 | 49.16 | 50.32 | 49.30 | 51.17 | 51.09 | 49.27 | 50.32 | 53.06 | **46.21** | **55.61** | 53.83 | 54.75 | 54.60 | 65.64 | 65.78 | 64.64 | 76.56 |  | 76.46 | 71.43 | 64.44 | 60.76 | 61.55 | 63.75 | 63.04 | 69.85 | 59.99 | 61.66 | 61.56 | 58.76 | 58.97 | 60.99 | 57.71 | 58.27 | 60.96 |
| 22 | 47.68 | 49.46 | 50.00 | 49.24 | 50.36 | 49.13 | 51.78 | 50.72 | 48.95 | 49.77 | 53.63 | **46.56** | **56.25** | 54.27 | 55.15 | 55.04 | 61.53 | 62.12 | 59.64 | 71.76 | 76.46 |  | 73.71 | 64.18 | 60.90 | 61.75 | 64.08 | 63.37 | 69.71 | 59.64 | 62.05 | 61.99 | 59.64 | 59.71 | 61.93 | 59.23 | 59.06 | 61.42 |
| 23 | 48.16 | 49.85 | 49.87 | 49.50 | 50.91 | 49.55 | 52.47 | 50.91 | 51.18 | 52.19 | 55.13 | **47.14** | **56.42** | 55.00 | 55.72 | 55.54 | 59.28 | 61.47 | 56.12 | 67.61 | 71.43 | 73.71 |  | 65.16 | 62.00 | 63.63 | 66.56 | 65.01 | 70.11 | 62.44 | 65.04 | 65.56 | 64.13 | 61.96 | 63.96 | 61.68 | 61.46 | 62.93 |
| 24 | 49.23 | 50.43 | 50.26 | 49.70 | 51.09 | 50.25 | 50.70 | 50.31 | 48.40 | 50.39 | 52.57 | **45.09** | **52.49** | 51.34 | 51.95 | 51.80 | 61.61 | 59.86 | 61.40 | 63.19 | 64.44 | 64.18 | 65.16 |  | 61.02 | 61.65 | 61.87 | 60.72 | 65.11 | 58.77 | 60.53 | 60.38 | 58.14 | 59.17 | 59.04 | 57.86 | 58.26 | 60.97 |
| 25 | 47.36 | 49.15 | 49.24 | 48.94 | 50.04 | 48.54 | 51.37 | 50.04 | 49.81 | 50.19 | 53.32 | **45.36** | **54.20** | 53.04 | 53.92 | 53.80 | 60.43 | 62.37 | 54.87 | 58.51 | 60.76 | 60.90 | 62.00 | 61.02 |  | 81.13 | 69.09 | 68.09 | 66.65 | 66.16 | 67.21 | 62.64 | 59.92 | 59.55 | 57.31 | 61.27 | 59.95 | 59.54 |
| 26 | 47.78 | 49.36 | 48.88 | 48.66 | 49.75 | 48.81 | 50.95 | 51.15 | 49.85 | 51.17 | 53.13 | **46.65** | **54.19** | 53.26 | 54.09 | 53.99 | 61.60 | 63.29 | 55.21 | 58.59 | 61.55 | 61.75 | 63.63 | 61.65 | 81.13 |  | 68.87 | 68.48 | 68.43 | 66.35 | 68.56 | 64.23 | 61.87 | 59.60 | 58.45 | 59.99 | 59.58 | 59.52 |
| 27 | 49.82 | 51.36 | 51.42 | 50.73 | 52.30 | 50.73 | 53.53 | 52.13 | 52.86 | 53.20 | 56.93 | **47.30** | **57.22** | 56.12 | 57.23 | 57.06 | 59.85 | 62.91 | 54.90 | 62.08 | 63.75 | 64.08 | 66.56 | 61.87 | 69.09 | 68.87 |  | 79.73 | 70.98 | 64.44 | 67.73 | 66.19 | 66.25 | 62.27 | 60.23 | 61.33 | 61.36 | 62.44 |
| 28 | 47.93 | 49.57 | 49.46 | 48.95 | 50.18 | 48.85 | 51.16 | 50.64 | 51.13 | 52.16 | 55.12 | **45.14** | **55.56** | 54.68 | 55.63 | 55.49 | 60.59 | 63.73 | 56.12 | 61.01 | 63.04 | 63.37 | 65.01 | 60.72 | 68.09 | 68.48 | 79.73 |  | 71.62 | 63.00 | 65.29 | 63.32 | 61.20 | 59.29 | 59.91 | 60.28 | 60.65 | 62.10 |
| 29 | 49.40 | 51.13 | 51.17 | 50.98 | 52.26 | 51.04 | 52.86 | 52.23 | 51.19 | 52.11 | 55.81 | **46.73** | **56.73** | 55.90 | 56.64 | 56.49 | 63.75 | 66.28 | 60.47 | 68.41 | 69.85 | 69.71 | 70.11 | 65.11 | 66.65 | 68.43 | 70.98 | 71.62 |  | 63.40 | 65.36 | 64.74 | 64.08 | 62.17 | 61.96 | 61.81 | 61.47 | 64.00 |
| 30 | 48.42 | 50.28 | 50.08 | 49.85 | 51.06 | 49.46 | 51.93 | 51.02 | 52.24 | 52.28 | 55.03 | **46.04** | **55.47** | 54.79 | 55.79 | 55.66 | 57.43 | 58.07 | 52.82 | 57.66 | 59.99 | 59.64 | 62.44 | 58.77 | 66.16 | 66.35 | 64.44 | 63.00 | 63.40 |  | 86.77 | 71.73 | 63.41 | 61.21 | 59.43 | 62.52 | 60.30 | 59.86 |
| 31 | 48.95 | 50.45 | 50.29 | 50.00 | 51.41 | 50.05 | 52.46 | 52.06 | 53.70 | 53.89 | 56.98 | **47.51** | **56.34** | 56.15 | 56.91 | 56.86 | 57.58 | 58.93 | 53.63 | 59.20 | 61.66 | 62.05 | 65.04 | 60.53 | 67.21 | 68.56 | 67.73 | 65.29 | 65.36 | 86.77 |  | 73.85 | 66.99 | 63.21 | 61.41 | 63.45 | 62.15 | 62.16 |
| 32 | 49.32 | 51.02 | 51.16 | 50.81 | 52.24 | 50.60 | 52.40 | 51.62 | 52.15 | 52.81 | 54.94 | **45.91** | **56.34** | 55.21 | 56.16 | 55.94 | 58.50 | 59.51 | 54.37 | 60.06 | 61.56 | 61.99 | 65.56 | 60.38 | 62.64 | 64.23 | 66.19 | 63.32 | 64.74 | 71.73 | 73.85 |  | 69.27 | 63.20 | 61.59 | 62.57 | 61.91 | 60.68 |
| 33 | 46.85 | 48.09 | 47.98 | 47.54 | 48.98 | 47.70 | 50.13 | 49.84 | 50.29 | 50.64 | 53.73 | **46.37** | **54.00** | 53.41 | 54.57 | 54.45 | 55.92 | 59.92 | 51.41 | 57.74 | 58.76 | 59.64 | 64.13 | 58.14 | 59.92 | 61.87 | 66.25 | 61.20 | 64.08 | 63.41 | 66.99 | 69.27 |  | 62.85 | 61.43 | 62.11 | 62.55 | 60.80 |
| 34 | 50.48 | 51.76 | 51.99 | 51.46 | 52.93 | 51.35 | 54.03 | 53.39 | 52.24 | 52.29 | 56.54 | **47.34** | **56.73** | 55.80 | 56.82 | 56.59 | 54.99 | 56.84 | 52.67 | 57.56 | 58.97 | 59.71 | 61.96 | 59.17 | 59.55 | 59.60 | 62.27 | 59.29 | 62.17 | 61.21 | 63.21 | 63.20 | 62.85 |  | 76.26 | 72.92 | 66.95 | 67.98 |
| 35 | 48.24 | 49.40 | 49.71 | 49.04 | 50.32 | 48.93 | 51.69 | 50.75 | 50.36 | 50.33 | 55.11 | **45.87** | **55.03** | 54.54 | 55.66 | 55.39 | 55.42 | 56.49 | 52.99 | 59.25 | 60.99 | 61.93 | 63.96 | 59.04 | 57.31 | 58.45 | 60.23 | 59.91 | 61.96 | 59.43 | 61.41 | 61.59 | 61.43 | 76.26 |  | 73.12 | 65.30 | 67.77 |
| 36 | 48.44 | 50.44 | 50.51 | 49.94 | 51.33 | 49.63 | 52.23 | 51.38 | 49.94 | 49.99 | 54.54 | **45.32** | **55.49** | 54.23 | 55.24 | 55.15 | 55.58 | 57.31 | 52.68 | 57.02 | 57.71 | 59.23 | 61.68 | 57.86 | 61.27 | 59.99 | 61.33 | 60.28 | 61.81 | 62.52 | 63.45 | 62.57 | 62.11 | 72.92 | 73.12 |  | 65.56 | 67.11 |
| 37 | 48.30 | 50.13 | 50.44 | 49.84 | 51.20 | 49.49 | 52.40 | 51.65 | 51.66 | 52.13 | 56.04 | **46.73** | **56.62** | 55.20 | 56.29 | 56.21 | 55.33 | 56.89 | 52.07 | 56.23 | 58.27 | 59.06 | 61.46 | 58.26 | 59.95 | 59.58 | 61.36 | 60.65 | 61.47 | 60.30 | 62.15 | 61.91 | 62.55 | 66.95 | 65.30 | 65.56 |  | 75.94 |
| 38 | 49.69 | 51.65 | 51.67 | 51.20 | 52.45 | 50.86 | 53.48 | 52.24 | 51.19 | 51.78 | 57.20 | **46.88** | **57.29** | 56.56 | 57.74 | 57.58 | 57.75 | 57.67 | 55.58 | 59.11 | 60.96 | 61.42 | 62.93 | 60.97 | 59.54 | 59.52 | 62.44 | 62.10 | 64.00 | 59.86 | 62.16 | 60.68 | 60.80 | 67.98 | 67.77 | 67.11 | 75.94 |  |
