# Supplementary material for: Genome-based reclassification of the family Stappiaceae and assessment of environmental forcing with the report of two novel taxa, Flexibacterium corallicola gen. nov., sp. nov., and Nesiotobacter zosterae sp. nov., isolated from coral and seagrass
Source: PLoS One. 2025 May 15;20(5):e0322500. doi: 10.1371/journal.pone.0322500 (PMC12080928; doi:10.1371/journal.pone.0322500)
Supplement: S8 Table — (DOCX) [file pone.0322500.s013.docx]

**S8 Table. Metabolic pathways associated with the major CCA axis, correlated with habitat types of strains (false discovery rate [FDR] was used as a significance threshold in the enrichment analysis).**

| **Collection** | **FDR** | **No. of genes detected** | **No. of genes in the pathway** | **Pathway description** |
| --- | --- | --- | --- | --- |
| Sediment | 1.5E-13 | 5 | 6 | Mixed, incl. *nosl*, and nitrous-oxide reductase |
| Sediment | 2.5E-11 | 5 | 14 | Mixed, incl. nitrogen metabolism, and peptidase u32 |
| Sediment | 8.2E-10 | 5 | 28 | Mixed, incl. cytochrome d1 heme domain, and siroheme decarboxylase , *asnc*-like ligand binding domain |
| Sediment | 4.1E-09 | 5 | 40 | Mixed, incl. cytochrome c-like domain, and cytochrome c oxidase subunit i |
| Sediment | 4.8E-08 | 5 | 67 | Mixed, incl. nitrogen metabolism, and heme |
| Sediment | 1.3E-07 | 5 | 85 | Mixed, incl. heme, and nitrogen metabolism |
| Sediment | 2.3E-02 | 1 | 3 | Cytochrome c oxidase subunit II-like C-terminal |
| Sediment | 2.3E-02 | 1 | 3 | Parallel beta-helix repeats |
| Sediment | 3.1E-02 | 1 | 5 | Styrene degradation, and 4-hydroxyphenylpyruvate dioxygenase |
| Sediment | 3.1E-02 | 1 | 5 | Pectin lyase fold |
| Sediment | 3.8E-02 | 1 | 8 | Cupredoxin |
| Sediment | 3.8E-02 | 1 | 8 | Pectin lyase fold/virulence factor |
| Sediment | 3.8E-02 | 1 | 7 | WD40/YVTN repeat-like-containing domain superfamily |
| Sediment | 4.5E-02 | 1 | 12 | Mixed, incl. styrene degradation, and 4-hydroxyphenylpyruvate dioxygenase |
| Sediment | 4.5E-02 | 1 | 11 | Glutathione S-transferase, C-terminal |
| Sediment | 4.5E-02 | 1 | 13 | Glutathione Transferase family |
| Sediment | 4.5E-02 | 1 | 13 | Glutathione S-transferase, N-terminal domain |
| Sediment | 4.5E-02 | 1 | 12 | Glutathione S-transferase, C-terminal domain |
| Sediment | 4.6E-02 | 1 | 14 | Mixed, incl. phosphonate and phosphinate metabolism, and ABC transporter, permease *phne*/*ptxc* |
| Sediment | 4.8E-02 | 1 | 17 | Glutathione S-transferase, C-terminal-like |
| Sediment | 4.9E-02 | 1 | 19 | AAA domain, putative AbiEii toxin, Type IV TA system |
| Flatworm | 1.5E-05 | 5 | 6 | Mixed, incl. *nosl*, and nitrous-oxide reductase |
| Flatworm | 1.4E-03 | 5 | 14 | Mixed, incl. nitrogen metabolism, and peptidase u32 |
| Flatworm | 1.4E-03 | 4 | 7 | Mixed, incl. dioxygenase, and coenzyme a transferase |
| Flatworm | 7.2E-03 | 3 | 5 | Mixed, incl. flagellar hook protein *flge*, and flagellar hook capping protein |
| Flatworm | 7.2E-03 | 8 | 67 | Mixed, incl. nitrogen metabolism, and heme |
| Flatworm | 7.2E-03 | 12 | 155 | Mixed, incl. bacterial regulatory proteins, *gntr* family, and galactose metabolism |
| Flatworm | 7.2E-03 | 5 | 21 | Pentose and glucuronate interconversions, and Pentose phosphate pathway |
| Flatworm | 7.2E-03 | 3 | 5 | KDPG/KHG aldolase, active site 2, and 6-phosphogluconate dehydratase |
| Flatworm | 7.5E-03 | 11 | 135 | Lyase |
| Flatworm | 7.5E-03 | 16 | 265 | Cytoplasm |
| Flatworm | 9.9E-03 | 4 | 15 | Degradation of aromatic compounds, and Coenzyme A transferase |
| Flatworm | 9.9E-03 | 4 | 15 | Carbohydrate metabolism |
| Flatworm | 9.9E-03 | 17 | 308 | Metal-binding |
| Flatworm | 1.0E-02 | 5 | 28 | Mixed, incl. cytochrome d1 heme domain, and siroheme decarboxylase , *asnc*-like ligand binding domain |
| Flatworm | 1.1E-02 | 4 | 16 | Mixed, incl. *nmt1*-like family, and *nnrs* |
| Flatworm | 1.4E-02 | 8 | 85 | Mixed, incl. heme, and nitrogen metabolism |
| Flatworm | 1.9E-02 | 4 | 19 | Thiamin diphosphate-binding fold |
| Flatworm | 2.4E-02 | 5 | 35 | Galactose metabolism, and Amino sugar and nucleotide sugar metabolism |
| Flatworm | 2.4E-02 | 7 | 73 | Mixed, incl. thiamine metabolism, and sulfur relay system |
| Flatworm | 2.6E-02 | 6 | 54 | Mixed, incl. pentose and glucuronate interconversions, and mandelate racemase-like |
| Flatworm | 3.0E-02 | 6 | 56 | Mixed, incl. *laci*-type *hth* domain, and galactose metabolism |
| Flatworm | 3.1E-02 | 3 | 11 | Pyruvate metabolism |
| Flatworm | 3.1E-02 | 3 | 11 | Thiamine pyrophosphate enzyme, C-terminal TPP-binding |
| Flatworm | 3.1E-02 | 8 | 101 | Isomerase |
| Flatworm | 3.1E-02 | 15 | 298 | Oxidoreductase |
| Flatworm | 3.1E-02 | 2 | 3 | Nickel insertion |
| Flatworm | 3.1E-02 | 5 | 40 | Mixed, incl. cytochrome c-like domain, and cytochrome c oxidase subunit i |
| Flatworm | 3.1E-02 | 8 | 105 | ABC transporter type 1, transmembrane domain MetI-like |
| Flatworm | 3.1E-02 | 8 | 105 | MetI-like superfamily |
| Flatworm | 3.1E-02 | 8 | 105 | Binding-protein-dependent transport system inner membrane component |
| Flatworm | 3.3E-02 | 23 | 577 | Transferase |
| Flatworm | 3.3E-02 | 3 | 12 | Thiamine pyrophosphate enzyme, C-terminal TPP binding domain |
| Flatworm | 4.1E-02 | 4 | 28 | Mixed, incl. *nmt1*-like family, and creatinine amidohydrolase |
| Flatworm | 4.1E-02 | 2 | 4 | KDPG/KHG aldolase |
| Flatworm | 4.1E-02 | 2 | 4 | Ribokinase/fructokinase |
| Flatworm | 4.1E-02 | 14 | 284 | P-loop containing nucleoside triphosphate hydrolase |
| Flatworm | 4.1E-02 | 8 | 114 | Iron |
| Flatworm | 4.1E-02 | 2 | 4 | Iron transport |
| Flatworm | 4.1E-02 | 2 | 4 | KDPG and KHG aldolase |
| Flatworm | 4.4E-02 | 5 | 47 | Mixed, incl. phosphonate and phosphinate metabolism, and phosphate transport |
| Sponge | 2.0E-04 | 6 | 20 | FecCD transport family, and Periplasmic binding protein |
| Sponge | 2.3E-04 | 9 | 73 | Mixed, incl. thiamine metabolism, and sulfur relay system |
| Sponge | 2.3E-04 | 6 | 26 | Mixed, incl. feccd transport family, and periplasmic binding protein |
| Sponge | 2.3E-04 | 20 | 386 | Nucleotide-binding |
| Sponge | 2.3E-04 | 16 | 265 | Cytoplasm |
| Sponge | 2.5E-04 | 11 | 127 | Mixed, incl. porphyrin and chlorophyll metabolism, and thiamine metabolism |
| Sponge | 2.5E-04 | 5 | 16 | Amino sugar and nucleotide sugar metabolism, and Ami_2 |
| Sponge | 6.3E-04 | 6 | 35 | Galactose metabolism, and Amino sugar and nucleotide sugar metabolism |
| Sponge | 6.3E-04 | 17 | 338 | ATP-binding |
| Sponge | 9.2E-04 | 7 | 56 | Mixed, incl. laci-type hth domain, and galactose metabolism |
| Sponge | 1.3E-03 | 3 | 5 | Mixed, incl. flagellar hook protein *flge*, and flagellar hook capping protein |
| Sponge | 1.3E-03 | 3 | 5 | Mixed, incl. *ami*_2, and beta-hexosaminidase |
| Sponge | 1.7E-03 | 9 | 111 | Kinase |
| Sponge | 2.0E-03 | 4 | 15 | Carbohydrate metabolism |
| Sponge | 3.1E-03 | 6 | 51 | Mixed, incl. nicotinate and nicotinamide metabolism, and terpenoid backbone biosynthesis |
| Sponge | 3.1E-03 | 3 | 7 | Nickel insertion, and Atrazine degradation |
| Sponge | 5.1E-03 | 5 | 36 | Mixed, incl. terpenoid backbone biosynthesis, and folate biosynthesis |
| Sponge | 5.5E-03 | 7 | 81 | Mixed, incl. laci-type hth domain, and oxidoreductase, n-terminal |
| Sponge | 7.1E-03 | 4 | 22 | Mixed, incl. molybdenum cofactor biosynthesis, and sulfur relay system |
| Sponge | 7.1E-03 | 6 | 61 | Mixed, incl. feccd transport family, and tonb-dependent receptor-like, beta-barrel domain superfamily |
| Sponge | 8.3E-03 | 20 | 577 | Transferase |
| Sponge | 1.4E-02 | 3 | 12 | Mixed, incl. nickel insertion, and atrazine degradation |
| Sponge | 1.4E-02 | 2 | 3 | Nickel insertion |
| Sponge | 1.5E-02 | 13 | 308 | Metal-binding |
| Sponge | 2.4E-02 | 12 | 284 | P-loop containing nucleoside triphosphate hydrolase |
| Sponge | 2.4E-02 | 3 | 15 | Molybdenum cofactor biosynthesis, and Sulfur relay system |
| Sponge | 2.4E-02 | 2 | 4 | Ribokinase/fructokinase |
| Sponge | 2.8E-02 | 3 | 16 | Mixed, incl. nmt1-like family, and nnrs |
| Sponge | 2.9E-02 | 7 | 114 | Mixed, incl. purine metabolism, and pyrimidine metabolism |
| Sponge | 3.1E-02 | 2 | 5 | Isoprene biosynthesis |
| Sponge | 3.1E-02 | 2 | 5 | Molybdenum, and Signal transduction histidine kinase, DctB (C4-dicarboxylate transport system regulator) |
| Sponge | 3.1E-02 | 2 | 5 | Mixed, incl. menaquinone biosynthesis, and nnrs |
| Sponge | 3.1E-02 | 2 | 5 | Mixed, incl. malf, n-terminal, and maltose/cyclodextrin abc transporter, substrate-binding protein |
| Sponge | 3.1E-02 | 2 | 5 | Siderophore-interacting protein, and FecCD transport family |
| Sponge | 3.1E-02 | 2 | 5 | ABC 3 transport family |
| Sponge | 3.4E-02 | 8 | 155 | Mixed, incl. bacterial regulatory proteins, gntr family, and galactose metabolism |
| Sponge | 3.7E-02 | 3 | 19 | Cilium, and Transglycosylase SLT domain |
| Sponge | 3.7E-02 | 2 | 6 | Lipopolysaccharide biosynthesis |
| Sponge | 3.7E-02 | 2 | 6 | Coenzyme A biosynthesis, and Pantothenate biosynthesis |
| Sponge | 3.7E-02 | 2 | 6 | ABC transporter periplasmic binding domain |
| Sponge | 3.7E-02 | 2 | 6 | Molybdenum cofactor biosynthesis |
| Sponge | 3.7E-02 | 2 | 6 | Periplasmic binding protein |
| Sponge | 3.7E-02 | 2 | 6 | Phosphomethylpyrimidine kinase |
| Sponge | 3.7E-02 | 6 | 96 | Ligase |
| Sponge | 4.6E-02 | 7 | 134 | ABC transporter |
| Sponge | 4.8E-02 | 6 | 102 | Magnesium |
