# Supplementary material for: Genome-based reclassification of the family Stappiaceae and assessment of environmental forcing with the report of two novel taxa, Flexibacterium corallicola gen. nov., sp. nov., and Nesiotobacter zosterae sp. nov., isolated from coral and seagrass
Source: PLoS One. 2025 May 15;20(5):e0322500. doi: 10.1371/journal.pone.0322500 (PMC12080928; doi:10.1371/journal.pone.0322500)
Supplement: S9 Table — (DOCX) [file pone.0322500.s014.docx]

| **(A) *Pannonibacter*** | | | | | |
| --- | --- | --- | --- | --- | --- |
|  | *‘Pannonibacter anstelovis’* Pt1^T^ | *Pannonibacter phragmietetus* NCTC13350^T^ | *Pannonibacter indicus* DSM23407^T^ | *Pannonibacter carbonis* Q4.6^T^ | *‘Pannonibacter tanglangensis’* XCT-53^T^ |
| *‘Pannonibacter anstelovis’*  Pt1^T^ |  | 94.89 | 93.75 | 73.78 | 73.69 |
| *Pannonibacter phragmietetus* NCTC13350^T^ | 94.89 |  | 95.14 | 74.14 | 74.10 |
| *Pannonibacter indicus* DSM23407^T^ | 93.75 | 95.14 |  | 74.07 | 74.38 |
| *Pannonibacter carbonis*  Q4.6^T^ | 73.78 | 74.14 | 74.07 |  | 86.11 |
| *‘Pannonibacter tanglangensis’* XCT-53^T^ | 73.69 | 74.10 | 74.38 | 86.11 |  |

| **(B) *Algilabrenzia*** | | |
| --- | --- | --- |
|  | *Roseibium algae* H3510^T^ | *Algilabrenzia polysiphoniae* KACC19711^T^ |
| *Roseibium algae* H3510^T^ |  | 77.67 |
| *Algilabrenzia polysiphoniae* KACC19711^T^ | 77.67 |  |

| **(C) *Roseibium*** | | | | | | | | |
| --- | --- | --- | --- | --- | --- | --- | --- | --- |
|  | *Roseibium algicola* RMAR6-6^T^ | *Roseibium porphyridii* KMA01^T^ | *‘Roseibium sediminicola’* CAU1639^T^ | *Roseibium aggregatum* IAM12614^T^ | *Roseibium marinum* DSM17023^T^ | *Roseibium album* CECT5095^T^ | *Roseibium alexandri*i DFL-11^T^ | *Roseibium denhamense* JCM10543^T^ |
| *Roseibium algicola* RMAR6-6^T^ |  | 77.47 | 81.81 | 92.18 | 78.63 | 75.96 | 74.51 | 75.48 |
| *Roseibium porphyridii* KMA01^T^ | 77.47 |  | 78.40 | 77.44 | 76.25 | 77.64 | 74.43 | 75.57 |
| *‘Roseibium sediminicola’* CAU1639^T^ | 81.81 | 78.40 |  | 81.60 | 78.31 | 76.66 | 74.99 | 75.86 |
| *Roseibium aggregatum* IAM12614^T^ | 92.18 | 77.44 | 81.60 |  | 78.42 | 75.90 | 73.96 | 75.42 |
| *Roseibium marinum* DSM17023^T^ | 78.63 | 76.25 | 78.31 | 78.42 |  | 75.03 | 73.21 | 74.61 |
| *Roseibium album* CECT5095^T^ | 75.96 | 77.64 | 76.66 | 75.90 | 75.03 |  | 73.76 | 74.67 |
| *Roseibium alexandri*i DFL-11^T^ | 74.51 | 74.43 | 74.99 | 73.96 | 73.21 | 73.76 |  | 76.70 |
| *Roseibium denhamense* JCM10543^T^ | 75.48 | 75.57 | 75.86 | 75.42 | 74.61 | 74.67 | 76.70 |  |
